# Quantification of protein group coherence and pathway assignment using functional association

**DOI:** 10.1186/1471-2105-12-373

**Published:** 2011-09-19

**Authors:** Meghana Chitale, Shriphani Palakodety, Daisuke Kihara

**Affiliations:** 1 Department of Computer Science, Purdue University, 305 N. University Street, West Lafayette, Indiana, 47907, USA; 2 Department of Biological Sciences, Purdue University, 915 W. State Street, West Lafayette, Indiana, 47907, USA; 3 Markey Center for Structural Biology, College of Science, Purdue University, 915 W. State Street, West Lafayette, Indiana, 47907, USA

## Abstract

**Background:**

Genomics and proteomics experiments produce a large amount of data that are awaiting functional elucidation. An important step in analyzing such data is to identify functional units, which consist of proteins that play coherent roles to carry out the function. Importantly, functional coherence is not identical with functional similarity. For example, proteins in the same pathway may not share the same Gene Ontology (GO) terms, but they work in a coordinated fashion so that the aimed function can be performed. Thus, simply applying existing functional similarity measures might not be the best solution to identify functional units in omics data.

**Results:**

We have designed two scores for quantifying the functional coherence by considering association of GO terms observed in two biological contexts, co-occurrences in protein annotations and co-mentions in literature in the PubMed database. The counted co-occurrences of GO terms were normalized in a similar fashion as the statistical amino acid contact potential is computed in the protein structure prediction field. We demonstrate that the developed scores can identify functionally coherent protein sets, *i.e*. proteins in the same pathways, co-localized proteins, and protein complexes, with statistically significant score values showing a better accuracy than existing functional similarity scores. The scores are also capable of detecting protein pairs that interact with each other. It is further shown that the functional coherence scores can accurately assign proteins to their respective pathways.

**Conclusion:**

We have developed two scores which quantify the functional coherence of sets of proteins. The scores reflect the actual associations of GO terms observed either in protein annotations or in literature. It has been shown that they have the ability to accurately distinguish biologically relevant groups of proteins from random ones as well as a good discriminative power for detecting interacting pairs of proteins. The scores were further successfully applied for assigning proteins to pathways.

## Background

Elucidating the role of proteins is a central problem in molecular biology. Computational methods play indispensable roles in various aspects of the functional elucidation of proteins, including database searches [1,2], capturing motifs and features in sequences [3-7], structures [8-10], and in experimental data [11], as well as clustering of proteins by functional similarity [12]. The importance and expectations of computational methods are further highlighted in the systems biology where a flood of sequenced genomes and various types of omics data are awaiting functional elucidation [13-18].

Realizing weaknesses of conventional homology search methods, *e.g*. limited coverage in genome annotations and the need for homologous proteins [17-20], various new approaches for function prediction have been developed in the past decade. Those include methods which use the sequence information in an elaborated fashion [21-27], those which compare the global and local tertiary structure information [8], and methods which use large-scale experimental data of proteins [11,28-35].

Besides function prediction, computational methods are also required for the interpretation of large-scale experimental data in the biological context [12]. Omics data, such as protein-protein interaction networks [36-40], microarray gene expression data [41,42], expression data by mass spectrometry [43] or by RNAseq [44,45], provide rich source of information for systems-level understanding of the protein interplay. Clustering genes by functional similarity is an indispensable step in finding the underlying biological principles behind the observed data.

To enable the above mentioned computational function analyses, it is necessary to establish a measure that quantifies functional associations between proteins. Controlled vocabularies of annotation terms, such as the Gene Ontology (GO) [46], provide a convenient platform for handling text description of the roles of gene products (RNA and protein). GO classifies annotation terms into three domains, Biological Process (BP), Molecular Function (MF), and Cellular Component (CC). Terms in each domain are organized in a hierarchical fashion as a Directed Acyclic Graph (DAG). The similarity between a pair of GO terms or, more generally between two sets of GO terms can be defined in several different ways. Most simply, two sets of GO terms can be compared by head to head matching where the similarity can be determined by the number of common annotations from both the sets [47]. Based on the GO hierarchy, the similarity of two GO terms can be defined as the minimum path length between them on the GO DAG [47,48]. A better alternative to the minimum path is to consider the Lowest Common Ancestor (LCA) for a pair of GO terms in the hierarchy, for which the Information Content (IC) is computed [49-51]. Schlicker *et al*. have developed a score named funsim, which combines the similarity of GO terms in BP and MF domains based on IC of LCA [52]. In the Methods section we discuss their scoring scheme in details.

The pairwise *functional similarity *may be suitable for certain purposes, *e.g*. for evaluating the accuracy of function prediction or for investigating functional similarity between a particular protein to others (*e.g*. homologous proteins). However, the situation can be different in omics data analyses, where many genes rather than a pair need to be handled to identify the set of gene products that are working in *functionally coherent *fashion. *Functional coherence *is exhibited in biologically relevant protein sets, for example, in the same biological pathways, subcellular localizations, the same protein complexes, proteins involved in the same stage of development, and disease. Importantly, proteins in a *functionally coherent *set may not necessarily have the same or similar GO terms in all the three GO domains, but their GO terms should be coherent with respect to each other so that the aimed function can be performed in a coordinated fashion. As an illustration, consider proteins in the same KEGG pathway. These proteins have different MF annotations because they carry out different enzymatic reactions. Moreover, interestingly, in general they also do not necessarily share a common BP annotation. For example, the *pyruvate metabolism *pathway (KEGG pathway ID: 00620) has 33 proteins, which are annotated with 48 unique BP domain terms. Among them there are only 8 proteins that are annotated with *pyruvate metabolic process (GO:0006090) *and each of the rest of the 47 GO BP terms are assigned to fewer number of proteins. The data for all the 101 KEGG pathways of yeast has been made available as Additional File 1. This can be caused by several reasons. One of the reasons is that the classification of the whole metabolic pathway into sub-pathways may differ from database to database. For example, the KEGG pathway database is not constructed by referring to the Gene Ontology annotations of genes. Another reason is that sometimes proteins are annotated with a BP term at a different specificity (child/parent terms). And of course the incompleteness of GO annotation could be another reason. Thus, even if all the BP domain annotations for the set of proteins are known, it would not be trivial to decide if the set is coherent by simply applying the existing pairwise functional similarity measures.

There are only a handful of previous works done for assessing the functional coherence. A type of related works consider GO terms that are enriched in a protein group [30,34,35,53,54]. However, it was discussed that statistically significant enrichment of certain GO terms evaluated using the hypergeometric distribution often does not indicate functional units in biological pathways [55]. Recently, Chagoyen *et al*. treated BP annotations of proteins as a vector of GO terms and computed pairwise protein similarity using the cosine distance [56]. They compute overall homogeneity of a set by averaging all the pairwise similarities between proteins in the set, and further assess the statistical significance of the coherence score. Pandey *et al*. have extended the concept of pairwise common ancestors of GO terms to the set of most specific common ancestors of the annotation sets of two proteins [57,58]. They have studied this functional coherence measure in the context of topological proximity of proteins in PPI and domain-domain interaction networks. Zheng *et al*. [55] performed text mining on research papers in the MEDLINE database [59] to represent the semantic content of a document in terms of presence of topics in the document. The documents are associated with proteins, which provide the protein-topic association as a graph. Then, closeness of proteins on this graph is used to determine the functional coherence of a group of proteins.

In this work, we propose two association scores for GO terms, which are aimed to evaluate the functional coherence of sets of proteins. The proposed scores quantify the associations of GO terms as the frequency of co-occurrence in two different biological contexts, in the GOA [60] protein sequence annotations and in the PubMed database literature [59]. The former score is named the Co-occurrence Association Score (CAS) while the latter is named the PubMed Association Score (PAS). We quantify the GO term associations by applying a method used for computing the knowledge-based statistical potentials for amino acid contacts [61,62], which is widely used in protein structure prediction. Unlike existing works which define similarity based on the GO hierarchy, our scores directly reflect how well terms are associated in the actual biological context. Since the associations are not restricted to the GO hierarchy, we can quantify association between terms across different GO domains. The novel and advantageous characteristic of our scores is that they quantify the *functional coherence *and not necessarily the *similarity*. Recently the GO database has newly introduced the relationships between Molecular Function (MF) and Biological Process (BP) domains to represent biological knowledge about the pathways and roles of genes [63]. Compared with their recent effort, our approach is more general, flexible, and automatic in the sense that the considered associations include knowledge from within the GO hierarchy as well as outside its structure. Resulting GO term associations reflect the current actual annotations in the databases.

We demonstrate that the developed association scores can identify functionally coherent protein sets, *i.e*. proteins in the same KEGG pathways, cellular locations, and protein complexes better than the above mentioned existing methods. In addition, we also show that these functional coherence score can accurately assign proteins to the KEGG pathways where the proteins belong. The current approach can be easily applied to other biological data sources to mine the associations and other ontologies as well, since it is not assuming any underlined structure in the ontology.

## Results

### CAS and PAS coherence scores

We have developed two function association scores, the CAS and the PAS. The CAS quantifies the frequency of GO terms that co-occur in the gene annotations, while the PAS takes into account co-occurrence of GO terms in the PubMed abstracts. The Gene Ontology database used in this study contains 17,316 Biological Process (BP), 2,534 Cellular Component (CC), and 9,428 Molecular Function (MF) domain terms, which result in a total of 29,278 terms. Among over 857,201,284 possible GO term pairs, 5,610,201 pairs (0.654%) obtained a non-zero value for the CAS while 3,320,265 pairs (0.387%) had a non-zero PAS.

**Figure 1:**
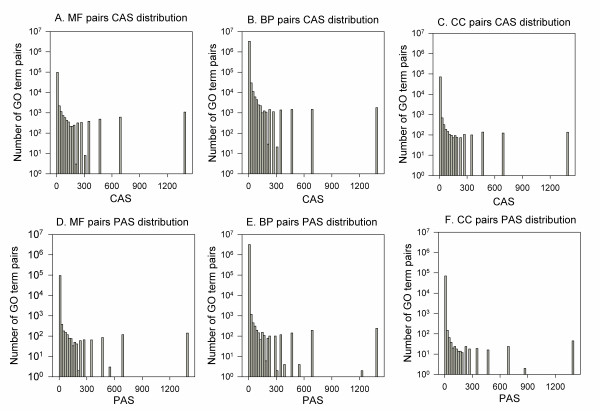
**CAS and PAS distribution for the same domain GO term pairs**. **A**, CAS distribution for GO term pairs in MF; **B**, CAS distribution for BP; **C**, CAS distribution in CC; **D**, PAS distribution for MF; **E**, PAS distribution for in BP; **F**, PAS distribution in CC. Out of 5,610,201 non-zero CAS values for GO term pairs, 107,673 (1.91%) were MF pairs, 3,430,135 (61.14%) were BP pairs, and 73,909 (1.31%) were CC pairs. Out of 3,320,265 non-zero PAS values for GO term pairs, 73,556 (2.21%) were MF pairs, 1,999,993 (60.23%) were BP pairs, and 51,816 (1.56%) were CC pairs.

**Figure 2:**
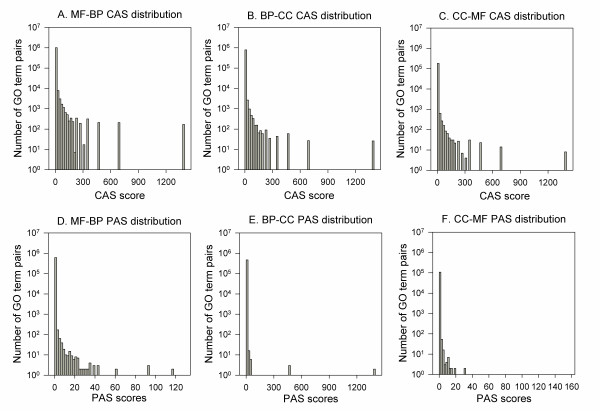
**CAS and PAS distribution for cross domain GO term pairs**. **A**, MF-BP CAS distribution; **B**, BP-CC CAS distribution; **C**, CC-MF CAS distribution; **D**, MF-BP PAS distribution; **E**, BP-CC PAS distribution; **F**, CC-MF PAS distribution. Out of 5,610,201 non-zero CAS values for GO term pairs, 1,026,484 (18.3%) were MF-BP pairs, 787,000 (14.0%) were BP-CC pairs and 183,001 (3.3%) were CC-MF pairs. Out of 3,320,265 non-zero PAS values for GO term pairs, 614,509 (18.5%) were MF-BP, 471,879 (14.2%) were BP-CC pairs, and 108,512 (3.3%) were CC-MF pairs.

A characteristic of the CAS and the PAS is that they capture the cross-domain associations between the GO terms. Out of 5,610,201 non-zero CAS, 1,996,485 (35.6%) are for cross-domain term pairs. As for PAS, which has in total of 3,320,265 GO terms pairs with non-zero scores, 1,194,900 (36.0%) are cross-domain terms. Distributions of GO term associations within the same domain (Figure 1) and across different domains (Figure 2) are compared. The CAS for the same domain (Figure 1ABC) and for the cross-domain (Figure 2ABC) shows similar distribution. On the other hand, a smaller number of high scoring cross-domain associations (Figure 2DEF) are observed for the PAS as compared with the same domain (Figure 1DEF). The peak observed at around 1400 in most of the histograms in Figures 1 and 2 are GO term pairs which only occur once in the GOA database or PubMed abstracts. Overall, the two figures show that a large number of cross-domain GO term associations were captured by the CAS and the PAS, which include pairs with significantly high scores.

**Figure 3:**
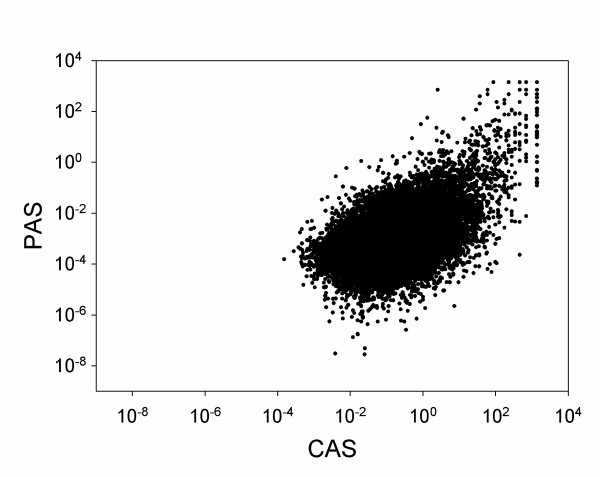
**Relationship between CAS and PAS for a sample set of GO terms pairs**. Out of the 5,610,201 GO term pairs for which CAS has been computed and 5,255,249 pairs for which PAS has been computed, we randomly sampled 50,000 pairs. Out of this 29,474 pairs were selected where both CAS and PAS are non zero values. The correlation coefficient of the two scores is 0.3084.

Figure 3 examines the correlations between the raw score values of the CAS and the PAS taken from 29,474 randomly sampled GO term pairs with positive scores for both CAS and PAS. The CAS and the PAS show a moderate correlation coefficient of 0.308. There are many GO term pairs like GO:0034087 *establishment of mitotic sister chromatid cohesion *and *GO:0030892 mitotic cohesin complex *where we observe higher CAS (19.3644) corresponding to higher PAS (4.2390). But for some cases, such as *GO:0000404 loop DNA binding *and *GO:0032139 dinucleotide insertion or deletion binding*, we obtained a higher CAS of 116.1866 and a lower PAS of 3.6299 × 10^-6^. Also for some cases like *GO:0019219 regulation of nucleobase, nucleoside, nucleotide and nucleic acid metabolic process *and *GO:0034404 nucleobase, nucleoside and nucleotide biosynthetic process *we find a lower CAS (0.00679) corresponding to a higher PAS (10.3277). Thus with the use of two scores that are based on different data sources we are able to capture much diverse relationships between terms.

**Figure 4:**
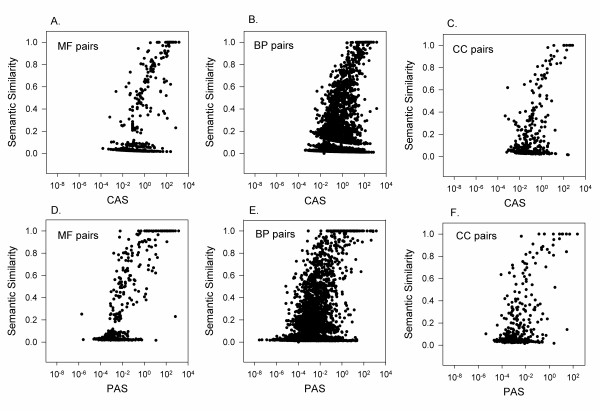
**Relationship of CAS and PAS with the semantic similarity scores**. **A**, CAS vs. semantic similarity for MF pairs (correlation coefficient: r = 0.5037); **B**, CAS vs. semantic similarity for BP pairs (r = 0.3450); **C**, CAS vs. semantic similarity for CC pairs (r = 0.4202); **D**, PAS vs. semantic similarity for MF pairs (r = 0.3023); **E**, PAS vs. semantic similarity for BP pairs (r = 0.1711); **F**, PAS vs. semantic similarity for CC pairs (r = 0.2613). The GO term pairs used in these plots are the same as the one used in Figure 3 (29,474 pairs). The semantic similarity has been computed using Eqn. 10. Since semantic similarity describes relationship between the terms of the same GO domain, the plots only include GO term pairs from the same domain (646 MF pairs, 17,731 BP pairs and 492 CC pairs).

We further compared the CAS and the PAS with the funsim score in Figure 4. Comparison was made separately for the three GO domains, because the funsim score is defined only for the GO term pairs in the same domain. Overall, the CAS and the PAS exhibit moderate correlation to the funsim score, with correlation coefficient values ranging from 0.504 (Figure 4A) to 0.171 (Figure 4E). However, there are interesting differences observed between the CAS and the PAS against the funsim score. There are GO pairs which are scored very low by the funsim score, close to zero, but have high CAS or PAS (right bottom corner of the plots). These examples include GO term pairs *GO:0051095 regulation of helicase activity *and *GO:0043570 maintenance of DNA repeat elements, GO:0000920 cytokinetic cell separation *and *GO:0034407 cell wall 1,3-beta-D-glucan metabolic process *in the BP domain by CAS (Figure 4B), and *GO:0009523 photosystem II *and *GO:0010287 plastoglobule *in the CC domain by PAS (Figure 4F). On the other hand, high scoring GO pairs by the funsim score are almost always scored high by the CAS and PAS. Thus, the CAS and the PAS do not lose the functional similarity that the funsim score captures and identify additional GO term pairs that are highly associated.

In Table 1 we show examples of the GO term pairs across different domains, which have a large score either by the CAS or the PAS. Because these are cross-domain associations, the funsim score is not defined. The first ten examples are cases with a large CAS and a small PAS. The first example is GO annotations assigned to luciferin 4-monooxygenase (UniProt Accession: *e.g*., Q27757 (*Photuris pennsylvanica *(Pennsylvania firefly), Q01158 (*Luciola lateralis *(firefly))), which emits florescent light. The second annotations are for chloroplastic ATP synthase gamma chain 1, (UniProt AC: Q01908). This protein is a component of the CF_0 _complex, which is embedded in thylakoid membrane and is important in regulating ATPase activity [64]. The next one is a DEAD box protein from the family of mitochondrial ATP-dependent RNA helicase (UniProt AC: P15424), which is an RNA chaperone and functions in mitochondrial group I and II intron splicing [65]. The fourth example is annotation for the ATP-binding cassette transporter sub-family G member 1 (UniProt AC P45844), which is a glycoprotein transporter responsible for negative regulation of cholesterol storage [66]. The fifth example with a high CAS is for lipopolysaccharide-binding protein (UniProt AC: P18428). It is involved in the acute-phase immunologic response to the gram-negative bacterial infections. Gram-negative bacteria contain a glycolipid, lipopolysaccharide (LPS), on their outer cell wall. Together with bactericidal permeability-increasing protein (BPI), the lipopolysaccharide-binding protein binds LPS and interacts with the cell surface pattern recognition receptor CD14 [67,68]. The Gene Ontology consortium has recently started providing links capturing the *part-of *relationship from Molecular Function (MF) term to the Biological Process (BP) term in which proteins plays the role [63]. The latter five examples in the upper half of the table provide links between MF terms and BP terms based on high CAS values, such that the same links are not currently present as *part-of *relationships in the latest version of GO database (2011-06). Thus the CAS and the PAS can be used to computationally obtain missing process-function links, for example, *GO:0019064 viral envelope fusion with host membrane *and *GO:0046812 host cell surface binding*, that can help increase the completeness of the relationships between the GO vocabulary terms.

**Table 1:** Examples of cross-domain GO term pairs which have a high CAS or PAS

GO ID 1	Description	Domain	GO ID 2	Description	Domain	CAS	PAS
GO:0047077	Photinus-luciferin 4-monooxygenase activity	MF	GO:0008218	Bioluminescence	BP	697.12	0.124
GO:0009544	Chloroplast ATP synthase complex	CC	GO:0009772	photosynthetic electron transport in photosystem II	BP	232.37	0.0572
GO:0033592	RNA strand annealing activity	MF	GO:0000373	Group II intron splicing	BP	116.19	0.0367
GO:0034437	Glycoprotein transporter activity	MF	GO:0010887	negative regulation of cholesterol storage	BP	232.37	0
GO:0051636	Gram-negative bacterial cell surface binding	MF	GO:0015920	lipopolysaccharide transport	BP	348.56	0.0195
GO:0047635	alanine-oxo-acid transaminase activity	MF	GO:0019481	L-alanine catabolic process, by transamination	BP	232.37	0.3489
GO:0046812	host cell surface binding	MF	GO:0019064	viral envelope fusion with host membrane	BP	116.18	0.0756
GO:0047558	3-cyanoalanine hydratase activity	MF	GO:0019499	cyanide metabolic process	BP	697.11	0.0653
GO:0047429	nucleoside-triphosphate diphosphatase activity	MF	GO:0009149	pyrimidine nucleoside triphosphate catabolic process	BP	116.18	0
GO:0033328	peroxisome membrane targeting sequence binding	MF	GO:0045046	protein import into peroxisome membrane	BP	199.17	2.690

GO:0031499	TRAMP complex	CC	GO:0034470	ncRNA processing	BP	1.316	4.317
GO:0070531	BRCA1-A complex	CC	GO:0016579	Protein deubiquitination	BP	4.497	5.164
GO:0031252	Cell leading edge	CC	GO:0070507	regulation of microtubule cytoskeleton organization	BP	0.0097	0.0115
GO:0030893	meiotic cohesin complex	CC	GO:0000819	sister chromatid segregation	BP	0.498	0.759
GO:0043566	structure-specific DNA binding	MF	GO:0033567	DNA replication, Okazaki fragment processing	BP	0.178	0.352
GO:0042781	3'-tRNA processing endoribonuclease activity	MF	GO:0034414	tRNA 3'-trailer cleavage, endonucleolytic	BP	232.37	116.186
GO:0000816	nicotinamide riboside kinase activity	MF	GO:0034356	NAD biosynthesis via nicotinamide riboside salvage pathway	BP	348.55	104.567
GO:0001735	prenylcysteine oxidase activity	MF	GO:0030328	prenylcysteine catabolic process	BP	232.37	22.487
GO:0004121	cystathionine beta-lyase activity	MF	GO:0019279	methionine biosynthetic process from L-homoserine via cystathionine	BP	139.42	15.154
GO:0070635	nicotinamide riboside hydrolase activity	MF	GO:0034356	NAD biosynthesis via nicotinamide riboside salvage pathway	BP	348.55	27.884

The table is divided into two halves with the first half containing examples of GO term pairs across different domains that have high CAS while the second half has examples with high PAS. For each of the term its description in the GO database and the domain (MF/BP/CC) where it belongs is provided. First five examples in each of the two halves are discussed in detail in the text whereas the latter five examples in each part provide missing process-function links that are detected by high CAS or PAS and are not currently linked using the *part-of *relationship in GO database.

The latter ten examples are cases where the PAS is higher than the CAS. Since the PAS ranges at lower values than the CAS (Figure 3), the substantial difference of the PAS and the CAS in these examples is more significant than they seem from the absolute score values. The first of these, TRAMP polyadenylation complex (e.g. UniProt AC: Q9P795), is involved in the post-transcriptional quality control mechanisms, including RNA surveillance and degradation of a wide range of nuclear RNAs including some of the non-protein coding RNA transcriptions (ncRNAs), by stimulating the 3' to 5' exonuclease activity of the exosome [69]. The second example is about BRCA1-A complex (e.g. UniProt AC: Q9NWV8), which binds to the k63 linked polyubiquitin chains present on the histone at the DNA damage sites and may facilitate the deubiquitinating activity of the deubiquitination enzyme BRCC36 [70]. The third GO pair is mined from the literature which reports the role of microtubules and actin filament networks in directed cell migration [71]. The cell leading edge refers to the area of a motile cell closest to the direction of motion which clearly requires actin microtubules for the movement. The next GO pair captures the information about sister chromatid cohesion during meiotic differentiation, which is mediated by a cohesion complex [72]. The fifth example is about the Calf 5' to 3' exo/endonuclease (the human counterpart of which is flap endonuclease-1) (e.g. UniProt AC: P39748) that is involved in the structure specific cleavage of DNA and processes Okazaki fragments during DNA replication [73]. The last five examples provide the missing links between MF and BP terms based on high PAS values, for example MF term *GO:0001735 prenylcysteine oxidase activity *is frequently mentioned in literature discussing a protein that plays a role in *GO:0030328 prenylcysteine catabolic process*.

In Table 2 and Table 3 we further provide examples of GO annotations that have high CAS or PAS for the certain GO terms. Table 2 lists five GO terms with highest CAS, thus, concurrent GO annotations of the query GO term which frequently co-annotate gene products. Table 3 is based on PAS, thus, the GO terms listed are frequently co-mentioned in the same PubMed abstracts. These concurrent GO annotations captured by the CAS and the PAS contribute to identification of proteins that are not necessarily annotated with the exact same highly related GO terms.

**Table 2:** Examples of concurrent GO terms based on CAS

GO ID	Description	Domain	Concurrent GO terms	Description	Domain	CAS
GO:0004359	glutaminase activity	MF	GO:0006543	glutamine catabolic process	BP	232.37
			GO:0006541	glutamine metabolic process	BP	32.051
			GO:0042819	vitamin B6 biosynthetic process	BP	19.364
			GO:0009065	glutamine family amino acid catabolic process	BP	15.754
			GO:0042816	vitamin B6 metabolic process	BP	11.618
GO:0004134	4-alpha-glucanotransferase activity	MF	GO:0000025	maltose catabolic process	BP	348.56
			GO:0010297	heteroglycan binding	MF	348.56
			GO:0004135	amylo-alpha-1,6-glucosidase activity	MF	348.56
			GO:0004133	glycogen debranching enzyme activity	MF	199.17
			GO:0000023	maltose metabolic process	BP	116.18
GO:0000719	photoreactive repair	BP	GO:0003904	deoxyribodipyrimidine photo-lyase activity	MF	278.84
			GO:0003913	DNA photolyase activity	MF	119.50
			GO:0006290	pyrimidine dimer repair	BP	73.381
			GO:0009650	UV protection	BP	7.536
			GO:0050660	FAD binding	MF	5.163
GO:0000733	DNA strand renaturation	BP	GO:0000405	bubble DNA binding	MF	139.42
			GO:0045002	double-strand break repair via single-strand annealing	BP	92.949
			GO:0000739	DNA strand annealing activity	MF	41.007
			GO:0043140	ATP-dependent 3'-5' DNA helicase activity	MF	36.690
			GO:0000217	DNA secondary structure binding	MF	20.503
GO:0000108	repairosome	CC	GO:0000111	nucleotide-excision repair factor 2 complex	CC	232.37
			GO:0000113	nucleotide-excision repair factor 4 complex	CC	174.28
			GO:0000715	nucleotide-excision repair, DNA damage recognition	BP	126.74
			GO:0031463	Cul3-RING ubiquitin ligase complex	CC	99.588
			GO:0000109	nucleotide-excision repair complex	CC	32.424

For the five GO terms in the left column, top five GO terms with the largest CAS score are listed.

**Table 3:** Examples of concurrent GO terms based on PAS

GO ID	Description	Domain	Concurrent GO terms	Description	Domain	PAS
GO:0004461	lactose synthase activity	MF	GO:0003945	N-acetyllactosamine synthase activity	MF	5.0101
			GO:0008378	galactosyltransferase activity	MF	0.7727
			GO:0003831	beta-N-acetylglucosaminylglycopeptide beta-1,4-galactosyltransferase activity	MF	0.7642
			GO:0005794	Golgi apparatus	CC	0.0082
			GO:0009312	oligosaccharide biosynthetic process	BP	0.0075
GO:0004842	ubiquitin-protein ligase activity	MF	GO:0019787	small conjugating protein ligase activity	MF	0.3263
			GO:0051438	regulation of ubiquitin-protein ligase activity	MF	0.3216
			GO:0016931	vasopressin activated calcium mobilizing receptor activity	MF	0.3212
			GO:0034450	ubiquitin-ubiquitin ligase activity	MF	0.3212
			GO:0042296	ISG15 ligase activity	MF	0.3212
GO:0034755	iron ion transmembrane transport	BP	GO:0034759	regulation of iron ion transmembrane transport	BP	13.0302
			GO:0005381	iron ion transmembrane transporter activity	MF	12.4638
			GO:0015087	cobalt ion transmembrane transporter activity	MF	3.2576
			GO:0070826	paraferritin complex	CC	2.6061
			GO:0070574	cadmium ion transmembrane transport	BP	1.3573
GO:0070637	pyridine nucleoside metabolic process	BP	GO:0070638	pyridine nucleoside catabolic process	BP	0.7536
			GO:0034356	NAD biosynthesis via nicotinamide riboside salvage pathway	BP	0.3015
			GO:0000816	nicotinamide riboside kinase activity	MF	0.2826
			GO:0006738	nicotinamide riboside catabolic process	BP	0.2029
			GO:0046495	nicotinamide riboside metabolic process	BP	0.1932
GO:0005833	hemoglobin complex	CC	GO:0031721	hemoglobin alpha binding	MF	0.0363
			GO:0031722	hemoglobin beta binding	MF	0.0308
			GO:0030492	hemoglobin binding	MF	0.0271
			GO:0020027	hemoglobin metabolic process	BP	0.0158
			GO:0020037	heme binding	MF	0.0083

For the five GO terms in the left column, top five GO terms with the largest PAS score are listed. PAS based concurrent terms are those which are associated frequently in the PubMed abstracts.

QuickGO [74], which is a recently built Gene Ontology browser, also provides functionality to browse co-occurring GO terms. This is similar to what the CAS captures but they have notable differences due to their diverse purposes. As the primary purpose of QuickGO is to browse the GO easily, it shows co-occurring GO terms for a specific query GO term. The score (named the S% score) used to sort the co-occurring terms for a specified GO term has direction (i.e. the score for A to B and B to A can be different). In contrast, the CAS is not directional as it is designed for identifying the biologically coherent protein groups by capturing the GO term association. Moreover, CAS also considers the associations of parental GO terms to capture more associations. And, of course, the PAS is totally different because it captures co-mentions in PubMed abstracts.

To summarize, the CAS and the PAS have moderate correlation with an existing score, funsim. The CAS and the PAS capture associations within the same domain as well relationship between cross-domain GO terms unlike funsim, which only defines the similarity between pair of GO terms from the same domain. Notably, CAS and PAS capture many biologically relevant cross-domain GO term associations (like MF-BP, BP-CC examples from Table 1) and thus can be used to obtain missing process-function links between GO terms as well as to find concurrent annotations across all the three GO domains.

### Coherence scores computed for biologically related protein sets

**Figure 5:**
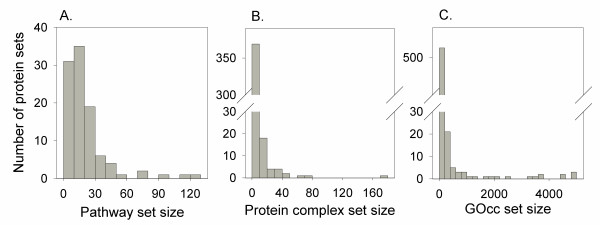
**The size of protein sets in the three datasets**. **A**, the KEGG pathway dataset; **B**, the protein complex dataset; **C**, the GOcc dataset.

Next, we examine how the CAS and the PAS scored, when accessing the functional coherence of biologically related protein sets. The functional coherence scores were developed using the CAS and the PAS (see Methods), which are aimed to capture the biologically related protein sets. Significance of the coherence scores is decided based on how well they are able to separate the biologically relevant protein sets from the randomly generated protein sets. This experiment is to show the proof of principle for the proposed functional coherence scores. We have used three datasets of functionally coherent protein sets in yeast, namely, KEGG pathways [75], proteins complexes [76], and protein groups annotated with the same GO Cellular Component terms (GOcc set) (Figure 5). There are 101 KEGG pathways with the number of proteins in each pathways ranging from 2 to 123 (Figure 5A). The number of protein complex sets is 400 (Figure 5B) [76]. The GOcc dataset includes 481 protein sets with the number of proteins in each set ranging from 2 to 100 (Figure 5C shows total number of sets). See Methods for more information.

In addition to the CAS and the PAS coherence scores developed here, we have also used three existing functional similarity scores, the modified funsim score [23], a score proposed by Chagoyen *et al*. [56] (termed the Chagoyen score), and a score by Pandey *et al*. [57,58] (the Pandey score). Briefly, the Chagoyen score computes the dot product of the information content of BP terms of proteins while the Pandey score considers the fraction of proteins in the database which are annotated by a common GO ancestor set of two proteins in question. An example of most specific pairwise common ancestor of terms *GO:0001948 glycoprotein binding *and *GO:0030492 hemoglobin binding *is their deepest shared GO ancestor term *GO:0005515 protein binding*. See Methods for derivation of the Chagoyen and the Pandey scores. For all the five scores, the coherence of a set of proteins is defined as the average of the scores for all the pairs of proteins.

Before analyzing the protein datasets in Figure 5, we have examined the dependence of the five scores to the size of the protein sets (Additional File 2: Figure S1). The verification was performed using 500 random yeast protein sets of sizes varying from 10 to 100 with an interval of 10. Since Figure S1 from Additional File 2 shows that the average scores do not significantly change by set sizes for all the five scores, we concluded that there is no need for normalization of the scores by the size of protein sets. To evaluate the statistical significance of the scores, we compute the p-value for all the coherence scores. The p-value assesses the number of proteins in the set that have a significantly higher coherent score as compared with the random chance (see Methods).

**Figure 6:**
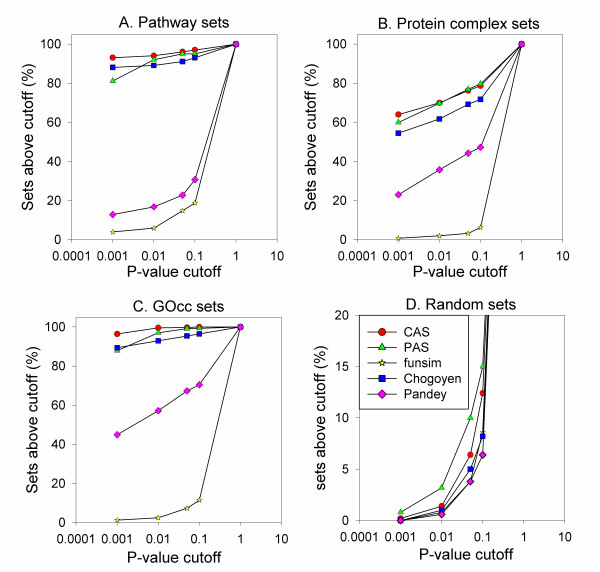
**Percentage of protein sets identified at different p-value cutoffs**. Each protein set is evaluated by p-value of the five coherence scores, CAS, PAS, funsim, Chagoyen, and Pandey, and those which have more significant p-value than the cutoff are counted. **A**, the KEGG pathway dataset; **B**, the protein complex dataset; **C**, the GOcc set; **D**, the random set.

In Figure 6, we computed the p-value of the five coherence scores for protein sets in the three datasets, the KEGG pathway sets (Figure 6A), protein complex sets (Figure 6B), and the GOcc sets (Figure 6C). The cumulative percentage of the protein sets with a p-value cutoff (x-axis) is counted. The same analyses were also performed on the randomly generated protein sets (Figure 6D). The raw score distributions for each of the three datasets are shown in the Additional File 2 (Figures S2, S3, S4). For the KEGG pathway sets (Figure 6A), the coherence scores by the CAS (Eqn. 5), the PAS (Eqn. 6), and the Chagoyen score (Eqn. 18) identified the majority of the sets with a significant p-value, as contrasted with the funsim score (Eqn. 12) and the Pandey score (Eqn. 23). At the p-value of 0.05, the CAS, the PAS, and the Chagoyen score identified 96.03%, 95.04%, and 91.08% of the KEGG pathways, respectively, while the funsim and the Pandey score recognized only 14.85% and 22.77%. Among the CAS, the PAS, and Chagoyen, the CAS showed the highest coverage at p-value of 0.05. The only KEGG pathways that did not have a significant p-value of less than 0.05 by the CAS were *Benzoate degradation via hydroxylation *(2 proteins; p-value: 0.2513), *Pentose and glucuronate interconversions *(7 proteins; p-value: 0.05783), *Ethylbenzene degradation *(3 proteins; p-value: 0.2738), and *alpha-Linolenic acid metabolism *(2 proteins; p-value: 0.3652).

Similar trends were observed for the protein complex sets (Figure 6B) and the GOcc sets (Figure 6C). For both datasets, the three scores (CAS, PAS, and Chagoyen) showed significantly better performance than Pandey and funsim scores. For the protein complex sets (Figure 6B), CAS, PAS, Chagoyen, Pandey, and funsim scores recognized 76.25%, 77.0%, 69.25%, 44.25%, and 3.25% of the protein sets, respectively, at the p-value cutoff of 0.05. In the case of the GOcc sets (Figure 6C), 99.79%, 99.16%, 95.42%, 67.35%, and 7.27% of the sets are recognized by CAS, PAS, Chagoyen, Pandey, and funsim scores, respectively. Figure 6D shows that the five scores do not provide significant p-value (0.05 or lower) to most of the randomly generated protein sets. Overall the CAS and the PAS showed better discriminative performance in identifying the functionally related protein sets than the other three existing scores compared.

**Figure 7:**
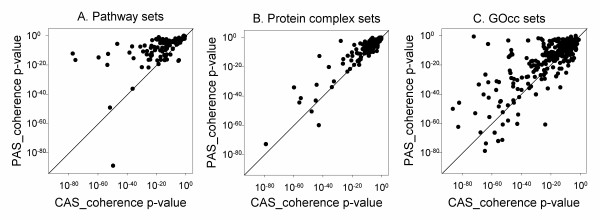
**Comparison of p-value of CAS_coherence and PAS_coherence scores**. **A**, Comparison on the pathway set; **B**, the protein complex dataset; **C**, the GOcc set.

In Figure 7, the p-values of the CAS and the PAS computed for the three datasets are compared. The p-values for the CAS coherence score showed a lower (*i.e*. more significant) value than the PAS p-values for many cases in the KEGG pathway sets (Figure 7A), the protein complex sets (Figure 7B), and the GOcc sets (Figure 7C), indicating that the CAS has higher discriminative power to select coherent groups of proteins than PAS. These differences in the p-value of the CAS and the PAS are also reflected in the previous results in Figure 6, where the CAS captured more protein sets than the PAS at a significant p-value cutoff.

### Coherence scores excluding obvious GO domain

Proteins in the same KEGG pathways are likely to share the similar GO terms in the BP domain (child/parent terms) used to describe the same biological process. Also proteins in the same group in the GOcc dataset have the same CC term by design. Here we reevaluate the CAS and the PAS coherence score for the KEGG pathway dataset and the GOcc dataset by excluding the apparently related GO domain. Note that the other three scores compared in Figure 6 also integrate BP and/or CC terms: The funsim score combines GO terms from all the three domains while the Pandey score uses BP and MF terms. The Chagoyen score only evaluates terms in the BP domain. However, we did not examine the effect of removing BP or CC terms from these three scores because the funsim and the Pandey score performed significantly more poorly than the PAS and the CAS (Figure 6) and removing BP or CC terms would simply further deteriorate the results. As for the Chagoyen score, it cannot be defined without BP terms.

**Figure 8:**
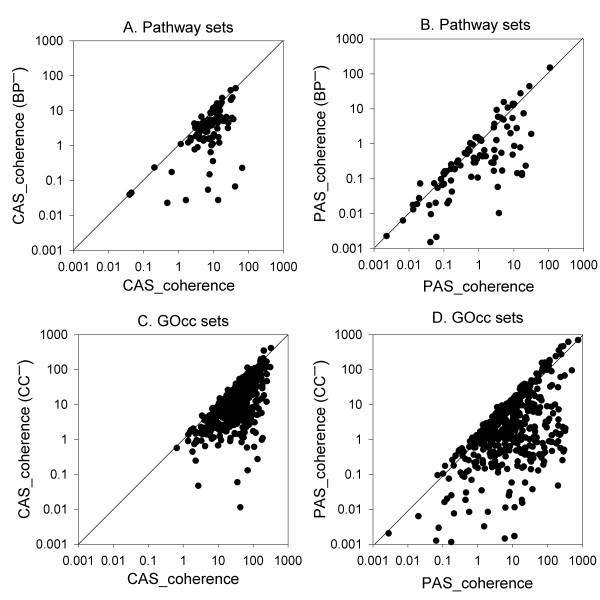
**Coherence score comparisons with/without obviously related GO domain**. For the pathway sets, the coherence scores were compared with and without the BP domain annotations. **A**, The CAS with all the three domains (the x-axis) while CAS_Coherence(BP^-^) (y-axis) is the CAS computed without BP terms. **B**, PAS coherence scores with and without BP terms. For the GOcc sets coherence scores were compared with and without (CC^-^) the use of CC domain annotations. **C**, CAS coherence; **D**, PAS coherence.

Figure 8 compares the CAS and the PAS coherence scores computed with and without BP or CC terms. The CAS and the PAS values drop for the majority of the KEGG pathways (Figures 8A, B) when BP terms were excluded, with an average score drop of 50.98% and 22.03%, respectively. When the CC terms are discarded in computing the CAS and the PAS for the GOcc data set, on average the score decreased by 55.62% (Figure 8C) and 50.46% (Figure 8D), respectively.

**Figure 9:**
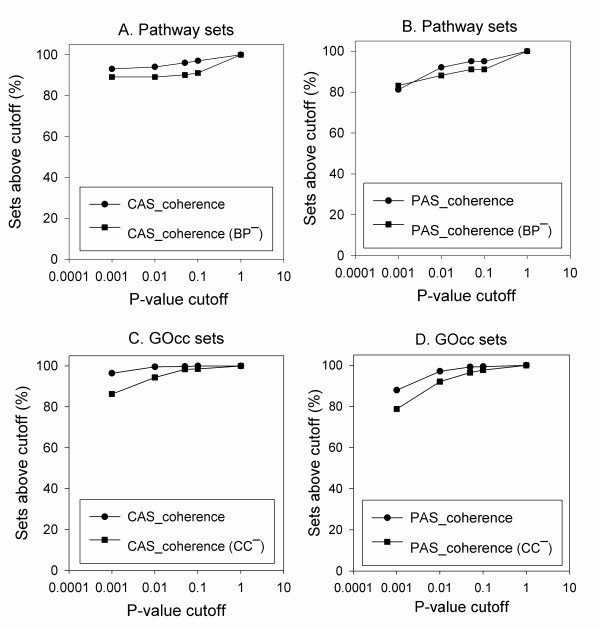
**Percentage of protein sets identified at different p-value cutoffs using partial annotation information**. The p-value of the CAS and the PAS coherence scores were computed with and without (BP^-^) BP domain annotations for the pathway dataset. **A**, CAS coherence; **B**, PAS coherence. The GOcc sets were evaluated with and without (CC^-^) CC domain annotations. **C**, CAS coherence; **D**, PAS coherence.

The drop of the scores (Figure 8) certainly results in the decrease of the fraction of protein sets recognized with a significant p-value. Figure 6 showed that 96.03% and 95.04% of KEGG pathway sets are recognized within the p-value cutoff of 0.05 by the original CAS and PAS, respectively. This fraction dropped to 90.09% and 91.08% when the BP terms were discarded for the CAS and the PAS (Figures 9A, B). These fractions of recognized KEGG pathways are still higher than those recognized by the Pandey and the funsim score (Figure 6A). Similarity, in the case of the GOcc dataset (Figures 9C, D), the fraction of the identified protein sets decreased from 99.79% to 98.33% by CAS and from 99.16% to 96.46% by PAS when CC terms are discarded (at the p-value cutoff of 0.05). These results are still better than the funsim and the Pandey score and comparable with the Chagoyen score (95.42%). Thus, removing the GO domain terms that are obviously related to the sets being analyzed, from both the coherence scores did not make a large reduction in the fraction of the identified protein sets.

### Detecting protein-protein interactions

Next, we test the proposed functional coherence scores on the protein-protein interaction (PPI) networks of yeast and human. We examine if the scores are able to detect the interacting proteins (true positives) as opposed to the non-interacting protein pairs (true negatives). The yeast PPI network contains 72,053 interacting protein pairs while 33,099 interactions are included in the human PPI data (see Methods). The same number of non-interacting protein pairs as the interacting protein pairs are extracted from the proteins included in the PPI networks. The p-value for pairs of proteins is computed for the CAS (Eqn. 3), the PAS (Eqn. 4), the funsim (Eqn. 11), the Chagoyen (Eqn. 17), and the Pandey (Eqn. 21) scores, and they are sorted in ascending order of the p-value. Then we computed the Receiver Operator Characteristic (ROC) curves for each scores on the yeast and the human PPI datasets.

**Figure 10:**
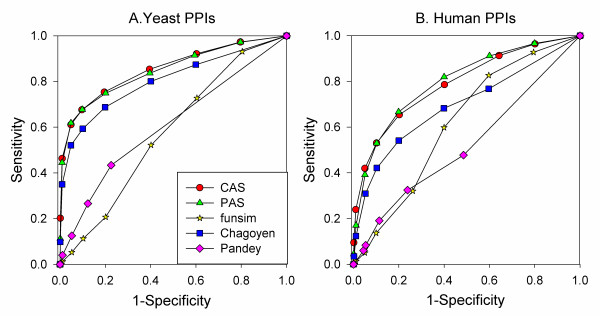
**ROC curves for detection of interacting protein pairs by functional similarity/association scores**. Protein pairs with significant p-value of the functional similarity/association scores (CAS, PAS, funsim, Chagoyen, and Pandey) were predicted to be interacting with each other. **A**, yeast PPI data; **B**, human PPI data.

The results on the yeast PPI dataset (Figure 10A) show that the CAS and the PAS obtained the maximum area under the ROC curves (AUC), 0.855 and 0.849, among the five scores compared. With the p-value cutoff of 0.05, CAS recognized 61.1% of the correct interacting pairs while the PAS identified 61.8% of them. The Chagoyen score came third, while using the funsim and the Pandey scores resulted in a significantly smaller AUC values. The five scores showed consistent results on the human PPI dataset (Figure 10B), although the AUC values decreased as compared to the results on the yeast PPI dataset. The CAS and the PAS showed almost identical AUC values, 0.791, and 0.800, and the Chagoyen score followed with an AUC value of 0.696. These results clearly show that the CAS and the PAS are better at distinguishing the positive interacting pairs from the non-interacting pairs. Indeed the performance of the five scores is also consistent with what was observed on the coherent protein datasets (Figure 6).

### KEGG pathway assignment

Finally, we used the functional coherence scores to predict the most likely KEGG pathway in which the protein plays a role. For a query protein the coherence score is computed against each KEGG pathway and then the pathways are sorted and ranked based on the coherence score. We examined if the correct pathway is scored at the top ranks. For this experiment, the KEGG pathway dataset which contains 101 pathways was used and cumulative percentages of proteins that are assigned correctly to their pathway were computed. Eight scores were compared. In addition to the CAS_coherence (Eqn. 7), PAS_coherence (Eqn. 8), funsim_coherence (Eqn. 14), GOscore_coherence_BP _(Eqn. 15) Chagoyen_coherence (Eqn. 19), and the Pandey_coherence (Eqn. 24), the CAS and the PAS were also computed without the BP annotations, CAS(BP^-^) and PAS(BP^-^). This is to remove the potentially apparent information of pathways encoded in the BP terms (*i.e*. proteins in the same KEGG pathway share the same BP terms in many cases). As for the funsim score, we have also used only BP annotations, which is referred as GOscore_coherence_BP_, because the funsim score did not perform well in the previous experiments in Figures 6 and 10.

**Figure 11:**
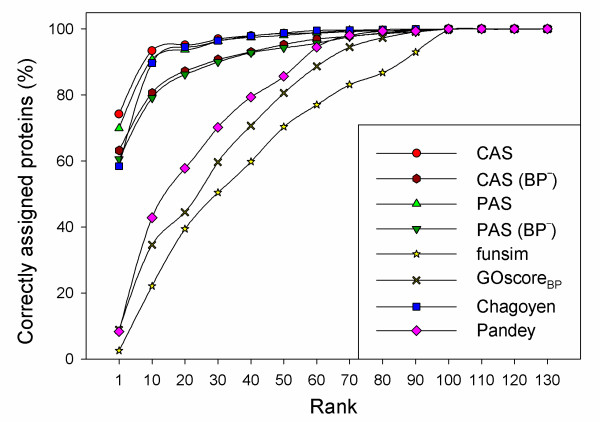
**Pathway assignment for yeast proteins**. Coherence score of the query protein with each KEGG pathway is used for ranking the KEGG pathways to indicate where the query protein is more likely to be assigned. The cumulative percentages of query proteins assigned to their correct pathway within the top X ranks are plotted. CAS, CAS(BP^-^) (without BP annotations), PAS, PAS(BP^-^) (without BP annotations), funsim, GOscore_coherence_BP _(using only BP annotations), Chagoyen, and Pandey were used.

As shown in Figure 11, the CAS and the PAS performed best with a remarkably high accuracy in identifying the KEGG pathway in which the query protein participates. For 74.2% of proteins CAS identified the correct KEGG pathway at the first rank while the PAS made correct assignment for 69.9% of the cases. When the top ten scoring KEGG pathways were considered, the CAS and the PAS assigned 93.38% and 90.76% of the proteins correctly to their KEGG pathways, respectively. Removing BP terms from the CAS and the PAS lowered the assignment accuracy, however, still maintained highly accurate KEGG pathway assignment relative to the other scores. The CAS(BP^-^) assigned 63.22% and 80.60% proteins at the first rank and within the top ten ranks, respectively, whereas the PAS(BP^-^) has similar accuracy with 60.56% (at the first rank) and 79.12% (within top ten ranks). The Chagoyen score made the correct pathway assignment for 58.4% of proteins at the first rank and 89.73% within the top ten ranks. The accuracy using the Chagoyen score within the tenth ranks is very close to those by the CAS and PAS and better than CAS(BP^-^) and PAS(BP^-^). However, note again that the Chagoyen score consists solely of BP terms and it is not defined without the BP domain. The funsim score and the Pandey score performed significantly worse than the CAS and the PAS in this experiment, too.

## Discussion

We have developed and critically analyzed coherence measures for a set of proteins, which can distinguish the biologically relevant sets from the random ones. By moving away from conventional methods, which rely on the hierarchical structure of the GO terms, we have designed a novel technique that can incorporate knowledge about the GO terms to find the strength of their association. The scores are computed based on the observed associations of the GO terms. The first score, Co-occurrence Association Score (CAS), considers the frequency that pairs of GO terms have been annotated to the same proteins. On the other hand, the PubMed Association Score (PAS) quantifies the number of occurrences that GO term pairs appear in literature abstracts as compared to the random chance. While most common form of the relationship defined by the GO is between the terms of the same domain (*is a *relation) where one term is a more specific representation of the other, there are some new relationships which connect MF-BP terms (*part of, regulates *relations). By using the CAS and the PAS we can automatically find the strength of associations between terms from any two domains of GO like MF-BP or BP-CC or CC-MF, and these associations are not restricted to the relationships provided by the GO hierarchy. About 36% of the CAS and the PAS associations are for cross-domain GO term pairs, and their scores are comparable to the same domain terms (Figures 1 &2). The CAS and the PAS capture different aspects of GO associations. While the CAS focuses on molecular level relationships of functional descriptions, the PAS often reveals the background knowledge of biologists.

To investigate the characteristics of the CAS and the PAS, we evaluated the two scores on three biologically coherent datasets, namely, the proteins in the same KEGG pathways, proteins that physically interact, and proteins which co-localize in a cell. The CAS and the PAS identified proteins in the same KEGG pathways, complexes, and co-localization with statistically significant scores (Figure 6) and were able to distinguish proteins which physically interact from those which do not (Figure 10). Moreover, the CAS and the PAS correctly assigned about 80-94% of proteins to the KEGG pathways they belong to within the top ten ranks. To the best of our knowledge, this is the first attempt to assign proteins to the KEGG pathways by evaluating the functional coherence. The performance of the CAS and the PAS was superior to the other related existing scores compared.

Counting associations of data is simple yet very powerful in revealing hidden rules behind the observed phenomena. Advanced techniques on considering data associations have been studied in the data mining and the machine learning area, which are applied, for example, in marketing [77-79]. Instead of the rather straightforward way of counting associations, using advanced methods, such as a measure of interestingness of association rules [80] and relational rule learning [81], would further improve the performance of the coherence scores. Specifically, the PAS may be further polished by applying text mining techniques that analyze the grammatical structure of sentences and relationships between phrases [82,83]. Furthermore, it will also be interesting to apply the same technique for evaluating the GO term co-occurrence in different biological contexts, such as gene expression data, regulatory pathways, and directly from PPI networks.

In this work we showed that the CAS and the PAS can identify biologically coherent proteins by capturing the GO term associations. The PAS and the CAS will also benefit for predicting biological function of un-annotated genes. Indeed there are previous works which use the GO term associations for predicting the gene function. King et al. [84] used co-occurring GO terms for predicting gene function by modeling relationships of GO terms with decision trees and Bayesian networks. Our group has developed a gene function prediction method, named PFP [22,23], which considers the GO term associations observed in a database in a similar way to the CAS. PFP first retrieves similar sequences to a query from a sequence database using PSI-BLAST [85], then, extracts GO terms which directly annotate the retrieved sequences as well as strongly associated GO terms to the GO annotations of the retrieved sequences. GO associations are described as conditional probabilities. The extracted GO terms are finally scored according to the frequency of the occurrence in the retrieved sequences and the E-values of the sequences. PFP achieved significantly higher prediction accuracy as compared with a naive way of using PSI-BLAST and some existing methods. Moreover, we can first predict the GO terms for un-annotated proteins by PFP and then apply PAS/CAS to identify which biological context the proteins play a role in.

An ultimate goal of biological studies is to understand the underlined structures and relationships of the biological entities which realize the observed phenomena. Such systematic understanding is accompanied with constructions of networks of relationships of terms in vocabularies that describe and label the biological entities. We believe that this work provides a pivotal step that brings us forward towards systematic understanding and description of a functions and mechanisms of proteins, organelle, cells, and higher level structures of life.

## Conclusions

Two function coherence scores were developed, one which reflects the co-occurrence of GO terms in protein annotations (CAS) and one which considers co-mentions of terms in the literature (PAS). The CAS and the PAS are shown to have the ability to accurately separate biologically relevant groups of proteins, *i.e*. proteins in the same pathways, protein complexes, and those with the same localization, from random sets. It was also shown that the CAS and the PAS can be used to detect physically interacting protein pairs. The scores were further successfully applied for assigning proteins to the KEGG pathways. The method can be readily applied to mine the functional associations between proteins from various biologically relevant sets.

## Methods

### Gene Ontology database

The hierarchical structure of Gene Ontology (GO) and GO term definitions are obtained from the Gene Ontology Consortium [46,86]http://archive.geneontology.org/ database version 2009-08. The Gene Ontology Annotation (GOA) database [60] version 2009-10 is used for the association between UniProt [87] identifiers and GO terms http://www.ebi.ac.uk/GOA/archive.html. Inferred Electronic Annotations (IEA) were excluded to increase the reliability of functional data. There are 46,686 protein - GO term association pairs for *Saccharomyces cerevisiae *(yeast) and 90,823 associations for *Homo sapiens *(human).

### PubMed Database

We used the NCBI's Entrez ESearch utility for obtaining the count of PubMed abstracts related to the particular GO terms. For example, for computing the PubMed association between terms *GO:0003700 *and *GO:0051169*, we first obtain their respective term definitions as *'transcription factor activity' *and *'nuclear transport' *from the GO database and remove words 'and, or, not' from their definitions. The remaining words in the definition are used to construct URL, *e.g*. http://eutils.ncbi.nlm.nih.gov/entrez/eutils/esearch.fcgi?db=pubmed&retmode=xml&rettype=full&term=transcription+factor+activity, which yields an xml that is then parsed to obtain the count of PubMed abstracts associated with the given term. For retrieving the counts of abstracts with two GO terms we appended the terms in the query URL and obtain the count. The ESearch query interface uses the MeSH indexing to incorporate the synonyms and the term variations. This provides us with a convenient way to retrieve the information that has been represented using different terms for the same concepts. The January 2010 version of the PubMed database was used.

### Biologically coherent sets of proteins

A coherent set of proteins are those which take part in the same biological context in a cell. For example, they can be a set of proteins playing roles in the same pathway, proteins involved in a disease or those responsible in a certain stage of development. Here we have prepared three types of coherent sets of yeast proteins: proteins in the same KEGG pathways, proteins included in the same protein complexes, and those which have the same subcellular localization. Along with these, two datasets of interacting protein pairs from yeast and human were prepared Details are described below. All the datasets are available at http://kiharalab.org/functionSim/.

#### Yeast KEGG pathway dataset

We downloaded yeast pathways from the ftp site of the Kyoto Encyclopedia of Genes and Genomes (KEGG) database [75]. This dataset consists of proteins in 101 pathways. The pathway size (the number of proteins in a pathway) ranges from 2 to 123 proteins with most of the pathways having around 20 proteins (Figure 5A). UniProtKB/Swiss-Prot database [87] (Version 2009-03) has been used for obtaining identifier mapping from KEGG database [75] identifiers and yeast SGD [88] identifiers.

#### Yeast protein complex dataset

For the yeast protein complex dataset, we have used a latest catalogue, YHTP2008 of 400 protein complexes compiled from genome-wide high throughput studies by Pu *et al*. [76]http://wodaklab.org/cyc2008/downloads. The catalogue provides protein complexes with Saccharomyces Genome Database (SGD) [88] identifiers, which are transferred to UniProt identifiers for associating them with the corresponding GOA annotations. The set sizes are shown in Figure 5B. Most of the protein complexes have about five or less component proteins with a few exceptions such as ribosomal complex whose size is 176.

#### Yeast GO cellular component (GOcc) datasets

We have constructed sets of yeast proteins with the same cellular component (CC) GO terms. Yeast proteins with non IEA GO annotations in the CC domain are selected from the GOA database. Then, for each such yeast proteins, CC terms are enriched by using the parental annotation transfers based on the true path in the GO hierarchy. Thus all ancestors of a GO term are incorporated as annotations for a protein. A total of 560 protein sets were obtained with sizes ranging from 2 to 4814. Very large protein sets contain proteins with a too general CC term. Therefore 481 sets with a size up to 100 were selected for analysis (Figure 5C).

### Protein-Protein Interaction (PPI) data

We have used *Saccharomyces cerevisiae *(budding yeast) and *Homo sapiens *(human) interaction data available at the BioGRID database [37] (version BIOGRID-2.0.56). In BioGRID data, only physical interactions and proteins with a UniProt identifier and with at least one GO annotation are used. The interactions are binary and thus no weight is associated with the edges in the PPI networks. For yeast and human, we have 72,053 and 33,099 interacting protein pairs, respectively. The number of proteins involved in the interactions is 4833 for yeast and 6241 for human.

In addition to the experimentally identified PPI networks, we have generated random protein-protein interactions. This is for two purposes, one for the null distribution of functional similarity scores for interacting proteins, and the other for computing the ROC curve. For both yeast and human proteins, 100,000 pairs each are randomly generated comprising of null distribution. For the ROC curve computation, we generate the same number of random interactions (false positive) as the actual interaction in each of the organisms.

### Co-occurrence Association Score (CAS)

The Co-occurrence Association Score (CAS) quantifies the frequency that two GO terms co-occur in annotation of a single gene relative to random chance. The CAS for two GO terms, *i *and *j*, is computed as follows:

(1) $$CAS\left(i,j\right)=\frac{\frac{C\left(i,j\right)}{\underset{i,j}{\sum }C\left(i,j\right)}}{\left(\frac{C\left(i\right)}{\underset{k}{\sum }C\left(k\right)}\right)\left(\frac{C\left(j\right)}{\underset{k}{\sum }C\left(k\right)}\right)}$$

Here *C(i) *is the number of sequences in the database which have GO term *i*. Similarly, *C(i,j) *is the number of sequences in the database which have a pair of GO terms, *i *and *j*. Thus, the numerator quantifies the fraction of sequences with annotations *i *and *j *relative to the total number of GO term pairs annotating the same proteins. The denominator is the expected number of times the two GO terms, *i *and *j*, co-occur in single proteins. This formulation is essentially similar to the method to compute a knowledge-based statistical amino acid contact potential [61,62].

For the GO terms annotating sequences in the GOA database, those with the evidence code of Inferred electronic annotations (IEA) are discarded. Along with the original annotations, parental GO terms to the original GO term annotations following the true path rule are also considered in computing the CAS. This procedure adds information of the GO hierarchy in the scoring scheme in an implicit fashion. GO pairs which do not co-occur in a gene are assigned with zero for their CAS.

### PubMed Association Score (PAS)

This score is based on the number of times a given pair of GO terms co-occurs in abstracts in the PubMed database at the National Center for Biotechnology Information (NCBI). Text definition of GO terms is obtained from the GO database. The text definition of two GO terms, *i *and *j*, are input in the Entrez ESearch web query interface to obtain the number of PubMed abstracts that have a given pair of terms. Along with a pair of terms, we obtain the number of PubMed abstracts which contain each individual term. Using the same equation (Eqn. 1) used for computing the CAS, the PubMed Association Score (PAS) for two GO terms, *i *and *j*, are defined as

(2) $$PAS\left(i,j\right)=\frac{\frac{Pub\left(i,j\right)}{\underset{i,j}{\sum }Pub\left(i,j\right)}}{\left(\frac{Pub\left(i\right)}{\underset{k}{\sum }Pub\left(k\right)}\right)\left(\frac{Pub\left(j\right)}{\underset{k}{\sum }Pub\left(k\right)}\right)}=\frac{Pub\left(i,j\right)}{Pub\left(i\right)Pub\left(j\right)}∙\frac{{\left(\underset{k}{\sum }Pub\left(k\right)\right)}^{2}}{\underset{k,l}{\sum }Pub\left(k,l\right)}$$

where *Pub(i, j) *is the number of PubMed abstracts which have two GO terms *i *and *j*, and *Pub(i) *is the number of abstracts which have a GO term *i*. Because PubMed includes nearly 19 million references, it is computationally challenging to obtain the exact total number of abstracts for all the co-occurring pairs in the database, *Σ_k, l_Pub(k, l)*. Thus, for the second term, which can be considered as a scaling factor for *PAS(i, j)*, the corresponding value computed for the CAS in Eqn. 1 is used.

### Protein pair association measure

A protein is usually annotated with multiple GO terms. Using the CAS and the PAS, we evaluate how well two sets of annotations from two proteins are associated. For two proteins, *P_x _*and *P_y _*with *A_x _*and *A_y _*number of annotations, respectively, the score is defined as follows:

(3) $$CAS\text{\_}prot\left(Px,Py\right)=\max\left(\frac{1}{Ax}\overset{Ax}{\underset{i=1}{\sum }}\begin{array}{c} \max\\ j=1..Ay \end{array}\left(CAS\left(Pxi,Pyj\right)\right),\;\frac{1}{Ay}\overset{Ay}{\underset{i=1}{\sum }}\begin{array}{c} \max\\ j=1..Ax \end{array}\left(CAS\left(Pxj,Pyi\right)\right)\right)$$

(4) $$PAS\text{\_}prot\left(Px,Py\right)=\max\left(\frac{1}{Ax}\overset{Ax}{\underset{i=1}{\sum }}\begin{array}{c} \max\\ j=1..Ay \end{array}\left(PAS\left(Pxi,Pyj\right)\right),\;\frac{1}{Ay}\overset{Ay}{\underset{i=1}{\sum }}\begin{array}{c} \max\\ j=1..Ax \end{array}\left(PAS\left(Pxj,Pyi\right)\right)\right)$$

*P_xi _*refers to the *i^th ^*annotation of protein *P_x_*. Thus, each annotation for *P_x _*is compared with all GO terms from *P_y_*, and the one which gives the maximum score is chosen. Then the best matching score for each *P_xi _*is averaged by *1/A_x_*. The same procedure is performed for *P_y_*, and a larger value is taken as the CAS or the PAS association between the two proteins, *P_x _*and *P_y_*. This matrix based comparison is proposed by Schlicker *et al*. [52].

### Protein set coherence score

Now we compute the functional homogeneity of a set *S *of *n *proteins, termed as a coherence score of a set, using either CAS_prot or PAS_prot score. Note that these scores are commutative, *i.e*. CAS_prot (A, B) *= *CAS_prot(B, A) and PAS_prot(A, B) *= *PAS_prot(B, A).

(5) $$CAS\_coherence\left(S\right)=\frac{1}{n\cdot \left(n-1\right)/2}\sum_{i=1}^{n}\sum_{j=i+1}^{n}CAS\_prot\left(Si,Sj\right)$$

(6) $$PAS\_coherence\left(S\right)=\frac{1}{n\cdot \left(n-1\right)/2}\sum_{i=1}^{n}\sum_{j=i+1}^{n}PAS\_prot\left(Si,Sj\right)$$

Specifically, the coherence of a protein *P *to a set of proteins, *S*, is defined as

(7) $$CAS\text{\_}coherence\left(S,P\right)=\frac{1}{n}\overset{n}{\underset{i=1}{\sum }}CAS\text{\_}prot\left(Si,P\right)$$

(8) $$PAS\text{\_}coherence\left(S,P\right)=\frac{1}{n}\overset{n}{\underset{i=1}{\sum }}PAS\text{\_}prot\left(Si,P\right)$$

### Semantic Similarity based coherence score

We compare the CAS and the PAS coherence score with three existing related scores, the semantic similarity score [52], a score designed by Chagoyen *et al*. [56] and another one by Pandey *et al*. [57,58]. The latter two scores will be explained in the subsequent sections.

The Semantic similarity measure was proposed to obtain the functional similarity between a pair of proteins [52]. The similarity between a pair of GO terms, *c1 *and *c2*, is quantified using the information content of the common ancestors of the two terms:

(9) $$sim\left(c1,c2\right)=\begin{array}{c} \max\\ c\in Ancestor\left(c1,c2\right) \end{array}\left(\frac{2\log\left(p\left(c\right)\right)}{\log p\left(c1\right)+\log p\left(c2\right)}\cdot \left(1-p\left(c\right)\right)\right)\;$$

*p(c) *is the fraction of proteins in the GOA database that are annotated with the GO term *c*, which is common ancestor of terms *c1 *and *c2*. Similarity between annotations of two proteins *P_x _*and *P_y_*, is defined in the same way as Eqns. 3 & 4:

(10) $$\begin{array}{c} GOscore_{GOcategory}\left(P_{x},P_{y}\right)=\\ \max\left\{\left(\left(\frac{1}{A_{x}}\overset{Ax}{\underset{i=1}{\sum }}\begin{array}{c} \max\\ 1\leq j\leq A_{y} \end{array}sim\left(P_{xi},P_{yj}\right)\right)\;,\left(\frac{1}{A_{y}}\overset{Ay}{\underset{j=1}{\sum }}\begin{array}{c} \max\\ 1\leq i\leq A_{x} \end{array}sim\left(P_{yi},P_{xj}\right)\right)\;\right\}\right)\; \end{array}$$

where the GO domain is either BP, MF, or CC. Note that GOscore is only computed for sets of GO terms in the same domain, since the semantic similarity score (Eqn. 9) uses the GO hierarchy structure. sim(P_xi_, P_yj_) is the semantic similarity score for two GO terms *P_xi _*and *P_yj_, A_x _*and *A_y _*are the number of terms in the two sets. A comprehensive score, funsim, combines the scores for the three domains [23]:

(11) $$funsim\left(Px,Py\right)=\frac{1}{3}\left(\begin{array}{c} {\left(\text{GOscor}{\text{e}}_{\text{BP}}\text{ (Px, Py)}\right)}^{2}\\ +{\left(\text{GOscor}{\text{e}}_{\text{MF}}\text{(Px, Py)}\right)}^{2}\\ +{\left(\text{GOscor}{\text{e}}_{\text{CC}}\text{(Px, Py)}\right)}^{2} \end{array}\right)\;$$

Each GOscore is squared following to the original funsim score proposed by Schlicker *et al*.

Parallel to Eqns. 5 and 6, the coherence score for a set of proteins, *S*, is defined as the average score between pairs of proteins in the set.

(12) $$funsim\_coherence\left(S\right)=\frac{1}{n\cdot \left(n-1\right)/2}\sum_{i=1}^{n}\sum_{j=i+1}^{n}funsim\left(Si,Sj\right)$$

(13) $$GOscore\_coherence_{GOcategory}\left(S\right)=\frac{1}{n\cdot \left(n-1\right)/2}\sum_{i=1}^{n}\sum_{j=i+1}^{n}GOscore_{GOcategory}\left(Si,Sj\right)$$

where *n *is the number of proteins in the set. Eqn. 12 quantifies coherence using the funsim score while Eqn. 13 is for GO terms of individual domain, BP, MF, or CC. The coherence of a protein *P *to a set of proteins, *S*, with *n *proteins, is defined as

(14) $$funsim\text{\_}conherence\left(S,P\right)=\frac{1}{n}\overset{n}{\underset{i=1}{\sum }}funsim\left(Si,P\right)$$

(15) $$GOscore\text{\_}coherence_{GOcategory}\left(S,P\right)=\frac{1}{n}\overset{n}{\underset{i=1}{\sum }}GOscore_{GOCategory}\left(Si,P\right)$$

### Chagoyen coherence score

Chagoyen *et al*. have designed a functional coherence score using solely BP annotations [56]. A protein is represented as a vector of weights for each of its BP annotations, where the weight of each term *i *is computed as the information content based on the number of proteins annotated with *i*, and *C(i) *is normalized by the total number of protein-GO term associations in the reference database (Eqn. 16).

(16) $$w\left(i\right)=-\ln\left(C\left(i\right)∕\underset{s}{\sum }\underset{t}{\sum }C\left(s_{t}\right)\right)$$

Here *s *denotes a sequence in the database and *s_t _*denotes a GO term in the sequence *s*. The functional similarity of two proteins, *P_i _*and *P_j_*, is defined as the dot product between vectors of *w(t) *for all GO terms (Eqn. 17). Parental terms for original GO annotations are also considered to incorporate the GO hierarchy.

(17) $$Chagoyen\text{\_}sim\left(Pi,Pj\right)=\frac{Pi\cdot Pj}{\left|Pi\right|\left|Pj\right|}$$

The functional coherence of a set of proteins and the coherence between a protein *P *and a set *S *are defined as the average of the score:

(18) $$Chagoyen\text{\_}coherence\left(S\right)=\frac{1}{n\cdot \left(n-1\right)∕2}\overset{n}{\underset{i=1}{\sum }}\overset{n}{\underset{j=i+1}{\sum }}Chagoyen\text{\_}sim\left(Pi,Pj\right)$$

(19) $$Chagoyen\text{\_}coherence\left(S,P\right)=\frac{1}{n}\overset{n}{\underset{i=1}{\sum }}Chagoyen\text{\_}sim\left(Si,P\right)$$

### Pandey coherence score

This functional similarity score for a protein pair uses a set of common ancestors of annotations of two proteins instead of aggregating pairwise similarity between annotations of both proteins [57,58]. Only the BP and the MF terms are used. The similarity of two GO terms, *c_i _*and *c_j_*, is defined as

(20) $$\lambda \left(ci,cj\right)=\begin{array}{c} \arg\max\\ c\in Ancestor_{Ci}\cap Ancestor_{Cj} \end{array}\left(-\log_{2}\frac{\left|G_{c}\right|}{\left|G_{r}\right|}\right)$$

*Ancestor*_*ci *_is the set of ancestors of the terms *c_i _*in the GO hierarchy. *G_c _*is the set of proteins associated with the term *c *and *G_r _*is the total set of proteins in the database. The functional similarity between a pair of proteins, *P_i _*and *P_j_*, with annotation sets *S_i _*and *S_j_*, respectively, is given by

(21) $$Pandey\text{\_}sim\text{(Pi,Pj)}=-\log_{2}\left(\frac{\left|G_{\Lambda \left(pi,pj\right)}\right|}{\left|Gr\right|}\right)$$

*G*_Λ(*pi, pj*) _is the set of proteins that are annotated by all the terms from the set Λ(*Pi, Pj*), the non redundant set of common ancestors between *S_i _*and *S_j_*. It is defined as

(22) $$\Lambda \left(P_{i},P_{j}\right)=\gamma \left(S_{i}\cup S_{j}\right)=\left\{c_{k}\in S_{i}\cup S_{j}:\exists \;no\;c_{l}\in S_{i}\cup S_{j}\;s.t.\;c_{l}\leq c_{k}\right\}$$

where *c_l _≤ c_k _*indicates *c_k _*is ancestor of *c_l_*.

The coherence score of a set of proteins *S *and that of between a protein *P *and set *S *are referred as Pandey_coherence. It is defined in the same way as aforementioned scores:

(23) $$Pandey\text{\_}coherence\left(S\right)=\frac{1}{n\cdot \left(n-1\right)∕2}\overset{n}{\underset{i=1}{\sum }}\overset{n}{\underset{j=i+1}{\sum }}Pandey\text{\_}sim\left(Pi,Pj\right)$$

(24) $$Pandey\text{\_}coherence\left(S,P\right)=\frac{1}{n}\overset{n}{\underset{i=1}{\sum }}Pandey\text{\_}sim\left(S,Pi\right)$$

### Statistical significance of coherence score of a protein set

We use the method proposed by Chagoyen *et al*. [56] to compute the statistical significance of the coherence score of a set of proteins with reference to the entire yeast (or human) genome. They have designed three methods for evaluating statistical significance, which showed similar performance in identifying functionally coherent sets in their work. Here we have used one of their methods. First we compute the coherence score for set *S *given by coherence(S), then number of proteins *P *in set *S *which satisfy the criteria sim(P, S) ≥ coherence(S) are obtained as *s*. Similarly for each protein *P *in the reference set *R *(whole genome), we find *r *proteins that satisfy the same criteria sim (P, S) ≥ coherence(S). R is the entire set of proteins in an organism. Now we can compute the p-value of coherence score of set *S *using hyper-geometric distribution for the number of proteins satisfying the given criteria. P-value is given by Eqn. 25 as the probability of observing *s *or more proteins satisfying the criteria, given that *r *proteins from reference set *R *satisfy the criteria.

(25) $$P-value=\overset{\left|S\right|}{\underset{i=s}{\sum }}\frac{\left(\begin{array}{c} r\\ i \end{array}\right)\left(\begin{array}{c} |R|-r\\ |S|-i \end{array}\right)}{\left(\begin{array}{c} \left|R\right|\\ \left|S\right| \end{array}\right)}$$

## Authors' contributions

MC participated in design, implemented the algorithms for analysis, and comparison of different techniques, and drafted the paper. DK conceived of the study, participated in its design, and finalized the manuscript. SP implemented the Chagoyen score. All authors read and approved the final manuscript.

## Supplementary Material

Additional file 1 **Analysis of GO Biological Process (BP) annotations of proteins in the KEGG yeast pathways**. For 101 KEGG pathways in yeast, the Biological Process (BP) GO annotations assigned to proteins in each pathway are counted. The pathway name, the number of proteins in the pathway, and the number of unique GO BP annotations have been provided in this file. Click here for file

Additional file 2 **Supplementary Figures S1-S4**. Coherence score distribution for different datasets. Click here for file
