# Supplementary material for: Quantification of protein group coherence and pathway assignment using functional association
Source: BMC Bioinformatics. 2011 Sep 19;12:373. doi: 10.1186/1471-2105-12-373 (PMC3189934; doi:10.1186/1471-2105-12-373)
Supplement: Additional file 1 — Analysis of GO Biological Process (BP) annotations of proteins in the KEGG yeast pathways. For 101 KEGG pathways in yeast, the Biological Process (BP) GO annotations assigned to proteins in each pathway are counted. The pathway name, the number of proteins in the pathway, and the number of unique GO BP annotations have been provided in this file. [file 1471-2105-12-373-S1.PDF]

Supplemental Material of
"Quantification of Protein Group Coherence and Pathway Assignment Using Functional Association"
by Meghana Chitale, Shriphani Palakodety, Daisuke Kihara

Contact: {mchitale, dkihara}@purdue.edu

For 101 KEGG pathways for yeast, Biological Process (BP) GO annotations assigned to proteins in each pathway are counted.
The pathway name, the number of proteins in the pathway, the number of unique GO BP annotations are shown in the following format:

Pathway Id| Pathway name| # of proteins| # of unique GO annotations

Then, the individual BP GO terms in the pathway are listed below with the number of proteins in the following format:

GO term ID Term Definition # of proteins in the pathway with the term

------
00190|Oxidative phosphorylation|76|46
GO:0000372 Group I intron splicing 2
GO:0006099 tricarboxylic acid cycle 4
GO:0015680 intracellular copper ion transport 1
GO:0009105 lipoic acid biosynthetic process 1
GO:0006897 endocytosis 1
GO:0045333 cellular respiration 5
GO:0034551 mitochondrial respiratory chain complex III assembly 1
GO:0006797 polyphosphate metabolic process 1
GO:0006796 phosphate metabolic process 1
GO:0005977 glycogen metabolic process 1
GO:0006123 mitochondrial electron transport, cytochrome c to oxygen 10
GO:0006122 mitochondrial electron transport, ubiquinol to cytochrome c 9
GO:0006121 mitochondrial electron transport, succinate to ubiquinone 4
GO:0006120 mitochondrial electron transport, NADH to ubiquinone 1
GO:0006316 movement of group I intron 1
GO:0009060 aerobic respiration 16
GO:0006314 intron homing 2
GO:0019538 protein metabolic process 1
GO:0015992 proton transport 4
GO:0016049 cell growth 1
GO:0015991 ATP hydrolysis coupled proton transport 1
GO:0007035 vacuolar acidification 14
GO:0007034 vacuolar transport 1
GO:0007033 vacuole organization 1
GO:0006885 regulation of pH 2
GO:0006119 oxidative phosphorylation 1
GO:0006116 NADH oxidation 3
GO:0006784 heme a biosynthetic process 2
GO:0055085 transmembrane transport 2
GO:0006461 protein complex assembly 4
GO:0019655 glucose catabolic process to ethanol 2
GO:0001300 chronological cell aging 2
GO:0015986 ATP synthesis coupled proton transport 16
GO:0034605 cellular response to heat 1
GO:0006623 protein targeting to vacuole 1
GO:0051259 protein oligomerization 1
GO:0043065 positive regulation of apoptosis 1

GO:0006879 cellular iron ion homeostasis 1
GO:0006878 cellular copper ion homeostasis 1
GO:0042407 cristae formation 1
GO:0006874 cellular calcium ion homeostasis 1
GO:0016226 iron-sulfur cluster assembly 1
GO:0018347 protein amino acid farnesylation 1
GO:0042493 response to drug 1
GO:0033617 mitochondrial respiratory chain complex IV assembly 2
GO:0008380 RNA splicing 2

00051|Fructose and mannose metabolism|27|30
GO:0006094 gluconeogenesis 2
GO:0008361 regulation of cell size 1
GO:0006000 fructose metabolic process 2
GO:0000032 cell wall mannoprotein biosynthetic process 2
GO:0006979 response to oxidative stress 3
GO:0006493 protein amino acid O-linked glycosylation 1
GO:0006635 fatty acid beta-oxidation 1
GO:0019388 galactose catabolic process 1
GO:0006013 mannose metabolic process 3
GO:0006800 oxygen and reactive oxygen species metabolic process 1
GO:0009651 response to salt stress 1
GO:0009298 GDP-mannose biosynthetic process 2
GO:0015992 proton transport 2
GO:0045047 protein targeting to ER 1
GO:0042843 D-xylose catabolic process 3
GO:0009636 response to toxin 5
GO:0006950 response to stress 1
GO:0046015 regulation of transcription by glucose 1
GO:0019568 arabinose catabolic process 3
GO:0032445 fructose import 2
GO:0046323 glucose import 3
GO:0034605 cellular response to heat 3
GO:0006486 protein amino acid glycosylation 2
GO:0001302 replicative cell aging 1
GO:0006006 glucose metabolic process 4
GO:0006110 regulation of glycolysis 1
GO:0019318 hexose metabolic process 1
GO:0006066 alcohol metabolic process 1
GO:0006003 fructose 2,6-bisphosphate metabolic process 1
GO:0006096 glycolysis 7

00052|Galactose metabolism|24|31
GO:0008361 regulation of cell size 1
GO:0006000 fructose metabolic process 2
GO:0005978 glycogen biosynthetic process 3
GO:0006979 response to oxidative stress 1
GO:0006078 1,6-beta-glucan biosynthetic process 1
GO:0006013 mannose metabolic process 3
GO:0019388 galactose catabolic process 3
GO:0006012 galactose metabolic process 1
GO:0006011 UDP-glucose metabolic process 3
GO:0005987 sucrose catabolic process 1
GO:0015992 proton transport 2
GO:0033499 galactose catabolic process via UDP-galactose 3
GO:0042843 D-xylose catabolic process 1
GO:0000411 positive regulation of transcription by galactose 2
GO:0006950 response to stress 1
GO:0046015 regulation of transcription by glucose 1
GO:0019568 arabinose catabolic process 1
GO:0032445 fructose import 2

GO:0000023 maltose metabolic process   1
GO:0006874 cellular calcium ion homeostasis   1
GO:0046323 glucose import   3
GO:0034605 cellular response to heat   3
GO:0006486 protein amino acid glycosylation   1
GO:0046835 carbohydrate phosphorylation 1
GO:0006008 glucose 1-phosphate utilization   2
GO:0005992 trehalose biosynthetic process   3
GO:0001302 replicative cell aging 1
GO:0006006 glucose metabolic process   3
GO:0015757 galactose transport   1
GO:0030003 cellular cation homeostasis   1
GO:0006096 glycolysis 5

03440|Homologous recombination|19|62
GO:0031292 gene conversion at mating-type locus, DNA double-strand break processing 1
GO:0000729 DNA double-strand break processing 1
GO:0051276 chromosome organization 1
GO:0000727 double-strand break repair via break-induced replication 7
GO:0045144 meiotic sister chromatid segregation   1
GO:0010520 regulation of reciprocal meiotic recombination 1
GO:0000724 double-strand break repair via homologous recombination 3
GO:0000723 telomere maintenance   2
GO:0051726 regulation of cell cycle   1
GO:0000722 telomere maintenance via recombination   9
GO:0043248 proteasome assembly   1
GO:0006289 nucleotide-excision repair   5
GO:0042138 meiotic DNA double-strand break formation 3
GO:0006511 ubiquitin-dependent protein catabolic process   1
GO:0006284 base-excision repair   3
GO:0016567 protein ubiquitination 2
GO:0000018 regulation of DNA recombination   1
GO:0006281 DNA repair 4
GO:0032079 positive regulation of endodeoxyribonuclease activity 1
GO:0032078 negative regulation of endodeoxyribonuclease activity 1
GO:0030437 ascospore formation   1
GO:0030435 sporulation resulting in formation of a cellular spore 1
GO:0032392 DNA geometric change   2
GO:0031573 intra-S DNA damage checkpoint 1
GO:0000070 mitotic sister chromatid segregation   1
GO:0007131 reciprocal meiotic recombination   6
GO:0030491 heteroduplex formation 7
GO:0006312 mitotic recombination   2
GO:0007004 telomere maintenance via telomerase 3
GO:0000712 resolution of meiotic joint molecules as recombinants 1
GO:0045132 meiotic chromosome segregation   1
GO:0006887 exocytosis 1
GO:0006278 RNA-dependent DNA replication 3
GO:0007064 mitotic sister chromatid cohesion   1
GO:0045005 maintenance of fidelity during DNA-dependent DNA replication 1
GO:0006273 lagging strand elongation   3
GO:0006272 leading strand elongation   3
GO:0045003 double-strand break repair via synthesis-dependent strand annealing   2
GO:0045002 double-strand break repair via single-strand annealing 6

GO:0006406 mRNA export from nucleus 1
GO:0043137 DNA replication, removal of RNA primer 3
GO:0051037 regulation of transcription, meiotic 1
GO:0007126 meiosis 1
GO:0001302 replicative cell aging 1
GO:0006338 chromatin remodeling 1
GO:0043161 proteasomal ubiquitin-dependent protein catabolic process 1
GO:0006303 double-strand break repair via nonhomologous end joining 3
GO:0006974 response to DNA damage stimulus 2
GO:0000709 meiotic joint molecule formation 2
GO:0006301 postreplication repair 4
GO:0000707 meiotic DNA recombinase assembly 3
GO:0000706 meiotic DNA double-strand break processing 2
GO:0045184 establishment of protein localization 2
GO:0006268 DNA unwinding during replication 3
GO:0006298 mismatch repair 3
GO:0000730 DNA recombinase assembly 3
GO:0006265 DNA topological change 1
GO:0042148 strand invasion 1
GO:0006260 DNA replication 3
GO:0010947 negative regulation of meiotic joint molecule formation 1
GO:0032508 DNA duplex unwinding 1
GO:0030447 filamentous growth 1

00100|Biosynthesis of steroids|23|8
GO:0008204 ergosterol metabolic process 1
GO:0008299 isoprenoid biosynthetic process 2
GO:0006696 ergosterol biosynthetic process 5
GO:0016114 terpenoid biosynthetic process 1
GO:0016126 sterol biosynthetic process 1
GO:0006897 endocytosis 1
GO:0016125 sterol metabolic process 1
GO:0045337 farnesyl diphosphate biosynthetic process 1

00920|Sulfur metabolism|13|13
GO:0019346 transsulfuration 2
GO:0006555 methionine metabolic process 4
GO:0009086 methionine biosynthetic process 3
GO:0006312 mitotic recombination 1
GO:0009636 response to toxin 1
GO:0019344 cysteine biosynthetic process 2
GO:0042493 response to drug 5
GO:0042538 hyperosmotic salinity response 1
GO:0006790 sulfur metabolic process 1
GO:0000103 sulfate assimilation 5
GO:0009117 nucleotide metabolic process 1
GO:0006878 cellular copper ion homeostasis 1
GO:0009092 homoserine metabolic process 1

00300|Lysine biosynthesis|15|12
GO:0009088 threonine biosynthetic process 1
GO:0018065 protein-cofactor linkage 1
GO:0019878 lysine biosynthetic process via aminoadipic acid 7
GO:0006555 methionine metabolic process 3
GO:0070154 mitochondrial lysyl-tRNA aminoacylation 1
GO:0042493 response to drug 3
GO:0032543 mitochondrial translation 1
GO:0009072 aromatic amino acid family metabolic process 1

GO:0006566 threonine metabolic process   2
GO:0016031 cytoplasmic tRNA import into mitochondrion   1
GO:0006430 lysyl-tRNA aminoacylation   1
GO:0034276 kynurenic acid biosynthetic process 1

00514|O-Mannosyl glycan biosynthesis|13|5
GO:0006487 protein amino acid N-linked glycosylation 1
GO:0007047 cell wall organization 1
GO:0006493 protein amino acid O-linked glycosylation 12
GO:0000032 cell wall mannoprotein biosynthetic process   2
GO:0006491 N-glycan processing   3

00480|Glutathione metabolism|19|22
GO:0042542 response to hydrogen peroxide 2
GO:0042493 response to drug 1
GO:0009051 pentose-phosphate shunt, oxidative branch 3
GO:0006979 response to oxidative stress 5
GO:0006740 NADPH regeneration   2
GO:0006635 fatty acid beta-oxidation   1
GO:0046689 response to mercury ion 1
GO:0008295 spermidine biosynthetic process   1
GO:0046686 response to cadmium ion 1
GO:0009409 response to cold 1
GO:0009408 response to heat 2
GO:0006102 isocitrate metabolic process 3
GO:0009263 deoxyribonucleotide biosynthetic process 3
GO:0006751 glutathione catabolic process 1
GO:0006750 glutathione biosynthetic process   2
GO:0045013 negative regulation of transcription by carbon catabolites
      1
GO:0006537 glutamate biosynthetic process   2
GO:0006597 spermine biosynthetic process 1
GO:0042908 xenobiotic transport   1
GO:0034605 cellular response to heat   2
GO:0015940 pantothenate biosynthetic process   3
GO:0009446 putrescine biosynthetic process   1

03030|DNA replication|30|51
GO:0000084 S phase of mitotic cell cycle 6
GO:0000278 mitotic cell cycle   2
GO:0006357 regulation of transcription from RNA polymerase II promoter
      1
GO:0000727 double-strand break repair via break-induced replication
      1
GO:0031939 negative regulation of chromatin silencing at telomere
      1
GO:0007047 cell wall organization 1
GO:0000724 double-strand break repair via homologous recombination
      2
GO:0000723 telomere maintenance   1
GO:0000722 telomere maintenance via recombination   2
GO:0006289 nucleotide-excision repair   10
GO:0006286 base-excision repair, base-free sugar-phosphate removal
      1
GO:0006284 base-excision repair   5
GO:0016567 protein ubiquitination 2
GO:0006281 DNA repair 4
GO:0000075 cell cycle checkpoint   1
GO:0030466 chromatin silencing at silent mating-type cassette   3
GO:0006348 chromatin silencing at telomere   7
GO:0030174 regulation of DNA replication initiation 1

GO:0007131 reciprocal meiotic recombination   2
GO:0030491 heteroduplex formation 2
GO:0006312 mitotic recombination   2
GO:0006343 establishment of chromatin silencing   1
GO:0006310 DNA recombination 1
GO:0007004 telomere maintenance via telomerase 2
GO:0006279 premeiotic DNA synthesis   1
GO:0006278 RNA-dependent DNA replication 4
GO:0000710 meiotic mismatch repair 1
GO:0007064 mitotic sister chromatid cohesion   2
GO:0007062 sister chromatid cohesion   4
GO:0045005 maintenance of fidelity during DNA-dependent DNA replication
      1
GO:0006273 lagging strand elongation   14
GO:0006272 leading strand elongation   13
GO:0006271 DNA strand elongation during DNA replication   5
GO:0016233 telomere capping 1
GO:0006270 DNA replication initiation   9
GO:0043137 DNA replication, removal of RNA primer   5
GO:0006401 RNA catabolic process   1
GO:0001302 replicative cell aging 2
GO:0006303 double-strand break repair via nonhomologous end joining
      2
GO:0006301 postreplication repair 4
GO:0000734 gene conversion at mating-type locus, DNA repair synthesis
      1
GO:0006269 DNA replication, synthesis of RNA primer 2
GO:0045184 establishment of protein localization   2
GO:0006268 DNA unwinding during replication   5
GO:0006267 pre-replicative complex assembly   6
GO:0000731 DNA synthesis during DNA repair   3
GO:0006266 DNA ligation   1
GO:0006298 mismatch repair   12
GO:0033260 DNA replication during S phase   1
GO:0006261 DNA-dependent DNA replication 1
GO:0006260 DNA replication   6

00410|beta-Alanine metabolism|8|14
GO:0006741 NADP biosynthetic process   1
GO:0006740 NADPH regeneration   1
GO:0006979 response to oxidative stress 1
GO:0019740 nitrogen utilization   1
GO:0009450 gamma-aminobutyric acid catabolic process 1
GO:0034605 cellular response to heat   3
GO:0019413 acetate biosynthetic process 3
GO:0008295 spermidine biosynthetic process   1
GO:0006067 ethanol metabolic process   1
GO:0006597 spermine biosynthetic process 1
GO:0006538 glutamate catabolic process   1
GO:0009651 response to salt stress 1
GO:0015940 pantothenate biosynthetic process   3
GO:0006090 pyruvate metabolic process   1

04011|MAPK signaling pathway - yeast|55|107
GO:0006970 response to osmotic stress   7
GO:0000767 cell morphogenesis during conjugation   1
GO:0030437 ascospore formation   1
GO:0007126 meiosis   1
GO:0007124 pseudohyphal growth   10
GO:0043193 positive regulation of gene-specific transcription   2
GO:0007121 bipolar cellular bud site selection 1

GO:0000011 vacuole inheritance   1
GO:0001403 invasive growth in response to glucose limitation   13
GO:0046685 response to arsenic   1
GO:0001402 signal transduction during filamentous growth   3
GO:0000208 nuclear translocation of MAPK involved in osmosensory signaling pathway 1
GO:0006470 protein amino acid dephosphorylation   1
GO:0000302 response to reactive oxygen species 1
GO:0030036 actin cytoskeleton organization   4
GO:0001302 replicative cell aging 3
GO:0043618 regulation of transcription from RNA polymerase II promoter in response to stress   2
GO:0006606 protein import into nucleus   1
GO:0032880 regulation of protein localization 1
GO:0007119 budding cell isotropic bud growth   2
GO:0030031 cell projection assembly   1
GO:0000755 cytogamy   2
GO:0000169 activation of MAPK activity involved in osmosensory signaling pathway 1
GO:0000754 adaptation to pheromone during conjugation with cellular fusion   2
GO:0007118 budding cell apical bud growth   2
GO:0031684 heterotrimeric G-protein complex cycle   3
GO:0000168 activation of MAPKK activity involved in osmosensory signaling pathway 2
GO:0000753 cellular morphogenesis during conjugation with cellular fusion   2
GO:0007117 budding cell bud growth 2
GO:0006270 DNA replication initiation   1
GO:0000751 cell cycle arrest in response to pheromone   1
GO:0000750 pheromone-dependent signal transduction involved in conjugation with cellular fusion   19
GO:0000161 MAPKKK cascade involved in osmosensory signaling pathway 1
GO:0001101 response to acid 4
GO:0006075 1,3-beta-glucan biosynthetic process   2
GO:0007015 actin filament organization   4
GO:0006406 mRNA export from nucleus   1
GO:0006468 protein amino acid phosphorylation 11
GO:0031385 regulation of termination of mating projection growth 2
GO:0051260 protein homooligomerization   1
GO:0031384 regulation of initiation of mating projection growth 2
GO:0009409 response to cold 1
GO:0006950 response to stress   3
GO:0009408 response to heat 3
GO:0046020 negative regulation of transcription from RNA polymerase II promoter by pheromones 1
GO:0000747 conjugation with cellular fusion   2
GO:0000743 nuclear migration during conjugation with cellular fusion 1
GO:0007266 Rho protein signal transduction   5
GO:0000742 karyogamy during conjugation with cellular fusion   1
GO:0007265 Ras protein signal transduction   1
GO:0007264 small GTPase mediated signal transduction 2
GO:0030969 UFP-specific transcription factor mRNA processing during unfolded protein response   3
GO:0030968 endoplasmic reticulum unfolded protein response 2
GO:0008361 regulation of cell size 3
GO:0030476 ascospore wall assembly 1
GO:0007165 signal transduction   4
GO:0043409 negative regulation of MAPKKK cascade   2

GO:0006357 regulation of transcription from RNA polymerase II promoter 1
GO:0045010 actin nucleation 1
GO:0001934 positive regulation of protein amino acid phosphorylation 1
GO:0006749 glutathione metabolic process 1
GO:0006350 transcription 1
GO:0048017 inositol lipid-mediated signaling 1
GO:0030010 establishment of cell polarity 7
GO:0010526 negative regulation of transposition, RNA-mediated 3
GO:0019243 methylglyoxal catabolic process to D-lactate 1
GO:0043065 positive regulation of apoptosis 1
GO:0034605 cellular response to heat 4
GO:0007050 cell cycle arrest 1
GO:0006897 endocytosis 3
GO:0010286 heat acclimation 2
GO:0051599 response to hydrostatic pressure 2
GO:0045944 positive regulation of transcription from RNA polymerase II promoter 3
GO:0050826 response to freezing 2
GO:0007243 protein kinase cascade 2
GO:0017196 N-terminal peptidyl-methionine acetylation 1
GO:0000132 establishment of mitotic spindle orientation 1
GO:0042542 response to hydrogen peroxide 2
GO:0051237 maintenance of RNA location 1
GO:0007047 cell wall organization 8
GO:0000917 barrier septum formation 1
GO:0007533 mating type switching 1
GO:0045449 regulation of transcription 1
GO:0006887 exocytosis 1
GO:0042149 cellular response to glucose starvation 2
GO:0001324 age-dependent response to oxidative stress during chronological cell aging 2
GO:0007234 osmosensory signaling pathway via two-component system 3
GO:0042631 cellular response to water deprivation 1
GO:0030447 filamentous growth 1
GO:0007232 osmosensory signaling pathway via Sho1 osmosensor 3
GO:0000282 cellular bud site selection 1
GO:0007231 osmosensory signaling pathway 4
GO:0000186 activation of MAPKK activity 1
GO:0000185 activation of MAPKKK activity 1
GO:0008298 intracellular mRNA localization 1
GO:0006033 chitin localization 1
GO:0000083 regulation of transcription of G1/S-phase of mitotic cell cycle 1
GO:0000082 G1/S transition of mitotic cell cycle 1
GO:0006979 response to oxidative stress 2
GO:0007096 regulation of exit from mitosis 3
GO:0007190 activation of adenylate cyclase activity 1
GO:0043433 negative regulation of transcription factor activity 1
GO:0042493 response to drug 1
GO:0007584 response to nutrient 1
GO:0000411 positive regulation of transcription by galactose 1
GO:0007329 positive regulation of transcription from RNA polymerase II promoter by pheromones 1
GO:0006972 hyperosmotic response 2

00252|Alanine and aspartate metabolism|26|34
GO:0006207 'de novo' pyrimidine base biosynthetic process 1
GO:0006094 gluconeogenesis 2

GO:0006189 'de novo' IMP biosynthetic process 1
GO:0006541 glutamine metabolic process   1
GO:0006979 response to oxidative stress 1
GO:0070146 mitochondrial aspartyl-tRNA aminoacylation   1
GO:0070145 mitochondrial asparaginyl-tRNA aminoacylation   1
GO:0006529 asparagine biosynthetic process   2
GO:0070143 mitochondrial alanyl-tRNA aminoacylation 1
GO:0019740 nitrogen utilization   1
GO:0032543 mitochondrial translation   1
GO:0006526 arginine biosynthetic process 2
GO:0006419 alanyl-tRNA aminoacylation   1
GO:0006524 alanine catabolic process   1
GO:0009450 gamma-aminobutyric acid catabolic process 1
GO:0006523 alanine biosynthetic process 1
GO:0070681 glutaminyl-tRNAGln biosynthesis via transamidation   2
GO:0009060 aerobic respiration   1
GO:0006164 purine nucleotide biosynthetic process   2
GO:0009437 carnitine metabolic process   2
GO:0006538 glutamate catabolic process   1
GO:0007029 endoplasmic reticulum organization 1
GO:0000053 argininosuccinate metabolic process 1
GO:0006532 aspartate biosynthetic process   1
GO:0000052 citrulline metabolic process 1
GO:0034605 cellular response to heat   4
GO:0006530 asparagine catabolic process 1
GO:0006422 aspartyl-tRNA aminoacylation 2
GO:0001302 replicative cell aging 1
GO:0006421 asparaginyl-tRNA aminoacylation   2
GO:0001300 chronological cell aging   1
GO:0006066 alcohol metabolic process   1
GO:0006995 cellular response to nitrogen starvation 1
GO:0045984 negative regulation of pyrimidine base metabolic process
      1

00380|Tryptophan metabolism|16|25
GO:0019655 glucose catabolic process to ethanol   1
GO:0042744 hydrogen peroxide catabolic process 1
GO:0006090 pyruvate metabolic process   2
GO:0006436 tryptophanyl-tRNA aminoacylation   1
GO:0001315 age-dependent response to reactive oxygen species   1
GO:0042631 cellular response to water deprivation   1
GO:0006741 NADP biosynthetic process   1
GO:0006740 NADPH regeneration   1
GO:0000949 aromatic amino acid family catabolic process to alcohol via
Ehrlich pathway   1
GO:0006559 L-phenylalanine catabolic process   1
GO:0030476 ascospore wall assembly 1
GO:0006800 oxygen and reactive oxygen species metabolic process 1
GO:0009651 response to salt stress 1
GO:0009409 response to cold 1
GO:0006103 2-oxoglutarate metabolic process   1
GO:0019413 acetate biosynthetic process 3
GO:0006569 tryptophan catabolic process 1
GO:0070183 mitochondrial tryptophanyl-tRNA aminoacylation 1
GO:0006950 response to stress   1
GO:0016233 telomere capping 1
GO:0000302 response to reactive oxygen species 2
GO:0034605 cellular response to heat   2
GO:0034354 de novo NAD biosynthetic process from tryptophan   4
GO:0006099 tricarboxylic acid cycle   1
GO:0006067 ethanol metabolic process   1

00251|Glutamate metabolism|25|31
GO:0006207 'de novo' pyrimidine base biosynthetic process 1
GO:0006189 'de novo' IMP biosynthetic process 1
GO:0006541 glutamine metabolic process 1
GO:0006540 glutamate decarboxylation to succinate 1
GO:0070149 mitochondrial glutamyl-tRNA aminoacylation 1
GO:0006979 response to oxidative stress 2
GO:0010133 proline catabolic process to glutamate 1
GO:0006807 nitrogen compound metabolic process 2
GO:0019740 nitrogen utilization 1
GO:0006526 arginine biosynthetic process 2
GO:0032543 mitochondrial translation 2
GO:0046037 GMP metabolic process 1
GO:0006524 alanine catabolic process 1
GO:0009450 gamma-aminobutyric acid catabolic process 2
GO:0006523 alanine biosynthetic process 1
GO:0070681 glutaminyl-tRNAGln biosynthesis via transamidation 2
GO:0009060 aerobic respiration 1
GO:0009435 NAD biosynthetic process 1
GO:0006538 glutamate catabolic process 1
GO:0006537 glutamate biosynthetic process 4
GO:0019676 ammonia assimilation cycle 3
GO:0007029 endoplasmic reticulum organization 1
GO:0006532 aspartate biosynthetic process 1
GO:0006562 proline catabolic process 1
GO:0034605 cellular response to heat 4
GO:0006425 glutaminyl-tRNA aminoacylation 1
GO:0006424 glutamyl-tRNA aminoacylation 1
GO:0006038 cell wall chitin biosynthetic process 1
GO:0001302 replicative cell aging 1
GO:0001300 chronological cell aging 1
GO:0045984 negative regulation of pyrimidine base metabolic process 1

00790|Folate biosynthesis|27|38
GO:0009396 folic acid and derivative biosynthetic process 3
GO:0009651 response to salt stress 1
GO:0033962 cytoplasmic mRNA processing body assembly 1
GO:0009268 response to pH 1
GO:0008153 para-aminobenzoic acid biosynthetic process 1
GO:0000753 cellular morphogenesis during conjugation with cellular fusion 1
GO:0000462 maturation of SSU-rRNA from tricistronic rRNA transcript (SSU-rRNA, 5.8S rRNA, LSU-rRNA) 1
GO:0006417 regulation of translation 1
GO:0006281 DNA repair 1
GO:0006415 translational termination 1
GO:0006730 one-carbon metabolic process 1
GO:0006760 folic acid and derivative metabolic process 1
GO:0006470 protein amino acid dephosphorylation 1
GO:0046855 inositol phosphate dephosphorylation 1
GO:0015961 diadenosine polyphosphate catabolic process 1
GO:0034063 stress granule assembly 1
GO:0000290 deadenylation-dependent decapping of nuclear-transcribed mRNA 1
GO:0000480 endonucleolytic cleavage in 5'-ETS of tricistronic rRNA transcript (SSU-rRNA, 5.8S rRNA, LSU-rRNA) 1
GO:0006406 mRNA export from nucleus 1
GO:0000388 spliceosome conformational change to release U4 (or U4atac) and U1 (or U11) 1

GO:0007129 synapsis 1
GO:0055085 transmembrane transport 1
GO:0007126 meiosis 1
GO:0006303 double-strand break repair via nonhomologous end joining 1
GO:0006302 double-strand break repair 1
GO:0006301 postreplication repair 1
GO:0006364 rRNA processing 6
GO:0006972 hyperosmotic response 1
GO:0000447 endonucleolytic cleavage in ITS1 to separate SSU-rRNA from 5.8S rRNA and LSU-rRNA from tricistronic rRNA transcript (SSU-rRNA, 5.8S rRNA, LSU-rRNA) 1
GO:0006814 sodium ion transport 1
GO:0006813 potassium ion transport 1
GO:0006268 DNA unwinding during replication 1
GO:0042149 cellular response to glucose starvation 1
GO:0000472 endonucleolytic cleavage to generate mature 5'-end of SSU-rRNA from (SSU-rRNA, 5.8S rRNA, LSU-rRNA) 1
GO:0000184 nuclear-transcribed mRNA catabolic process, nonsense-mediated decay 1
GO:0000348 nuclear mRNA branch site recognition 1
GO:0000027 ribosomal large subunit assembly 3
GO:0032508 DNA duplex unwinding 1

00513|High-mannose type N-glycan biosynthesis|12|10
GO:0007047 cell wall organization 1
GO:0006080 substituted mannan metabolic process 2
GO:0007114 cell budding 1
GO:0000032 cell wall mannoprotein biosynthetic process 6
GO:0006493 protein amino acid O-linked glycosylation 1
GO:0006491 N-glycan processing 2
GO:0006487 protein amino acid N-linked glycosylation 6
GO:0006486 protein amino acid glycosylation 3
GO:0000917 barrier septum formation 2
GO:0030433 ER-associated protein catabolic process 1

00510|N-Glycan biosynthesis|28|16
GO:0007047 cell wall organization 1
GO:0009272 fungal-type cell wall biogenesis 1
GO:0009060 aerobic respiration 2
GO:0034605 cellular response to heat 2
GO:0008610 lipid biosynthetic process 1
GO:0034203 glycolipid translocation 1
GO:0006493 protein amino acid O-linked glycosylation 1
GO:0006488 dolichol-linked oligosaccharide biosynthetic process 5
GO:0006490 oligosaccharide-lipid intermediate assembly 6
GO:0006461 protein complex assembly 2
GO:0006487 protein amino acid N-linked glycosylation 17
GO:0006506 GPI anchor biosynthetic process 1
GO:0006486 protein amino acid glycosylation 7
GO:0007049 cell cycle 1
GO:0030433 ER-associated protein catabolic process 1
GO:0018279 protein amino acid N-linked glycosylation via asparagine 2

00910|Nitrogen metabolism|13|19
GO:0006312 mitotic recombination 1
GO:0019346 transsulfuration 2
GO:0006546 glycine catabolic process 1
GO:0019344 cysteine biosynthetic process 1
GO:0019343 cysteine biosynthetic process via cystathionine 1

GO:0006544 glycine metabolic process   1
GO:0009086 methionine biosynthetic process   1
GO:0000096 sulfur amino acid metabolic process 1
GO:0042493 response to drug 1
GO:0006529 asparagine biosynthetic process   2
GO:0006807 nitrogen compound metabolic process 2
GO:0006878 cellular copper ion homeostasis   1
GO:0006537 glutamate biosynthetic process   3
GO:0019676 ammonia assimilation cycle   3
GO:0006534 cysteine metabolic process   1
GO:0034605 cellular response to heat   1
GO:0006530 asparagine catabolic process 1
GO:0006730 one-carbon metabolic process 1
GO:0006995 cellular response to nitrogen starvation 1

00061|Fatty acid biosynthesis|4|3
GO:0006998 nuclear envelope organization 1
GO:0006633 fatty acid biosynthetic process   2
GO:0006606 protein import into nucleus   1

03450|Non-homologous end-joining|10|24
GO:0006342 chromatin silencing   2
GO:0030437 ascospore formation   1
GO:0030435 sporulation resulting in formation of a cellular spore 1
GO:0030466 chromatin silencing at silent mating-type cassette   1
GO:0051037 regulation of transcription, meiotic   1
GO:0007126 meiosis   1
GO:0042138 meiotic DNA double-strand break formation 3
GO:0034502 protein localization to chromosome 1
GO:0043137 DNA replication, removal of RNA primer   1
GO:0032078 negative regulation of endodeoxyribonuclease activity 1
GO:0000727 double-strand break repair via break-induced replication 4
GO:0000724 double-strand break repair via homologous recombination 2
GO:0006303 double-strand break repair via nonhomologous end joining 9
GO:0000723 telomere maintenance   4
GO:0006302 double-strand break repair   2
GO:0006333 chromatin assembly or disassembly   2
GO:0000722 telomere maintenance via recombination   1
GO:0006287 base-excision repair, gap-filling   1
GO:0006286 base-excision repair, base-free sugar-phosphate removal 1
GO:0006281 DNA repair 2
GO:0007535 donor selection   1
GO:0000706 meiotic DNA double-strand break processing   2
GO:0001302 replicative cell aging 2
GO:0000734 gene conversion at mating-type locus, DNA repair synthesis 1

03022|Basal transcription factors|23|16
GO:0006338 chromatin remodeling   1
GO:0051123 transcriptional preinitiation complex assembly 10
GO:0000114 regulation of transcription during G1 phase of mitotic cell cycle 2
GO:0006289 nucleotide-excision repair   4
GO:0006367 transcription initiation from RNA polymerase II promoter 13
GO:0007070 negative regulation of transcription from RNA polymerase II

promoter, mitotic 4
GO:0006473 protein amino acid acetylation 5
GO:0032569 specific transcription from RNA polymerase II promoter 4
GO:0032568 general transcription from RNA polymerase II promoter 10
GO:0016573 histone acetylation 6
GO:0000717 nucleotide-excision repair, DNA duplex unwinding 3
GO:0006360 transcription from RNA polymerase I promoter 1
GO:0016568 chromatin modification 5
GO:0006383 transcription from RNA polymerase III promoter 1
GO:0006325 chromatin organization 5
GO:0000019 regulation of mitotic recombination 1

04020|Calcium signaling pathway|13|32
GO:0007114 cell budding 1
GO:0051300 spindle pole body organization 1
GO:0006468 protein amino acid phosphorylation 4
GO:0006607 NLS-bearing substrate import into nucleus 1
GO:0007265 Ras protein signal transduction 3
GO:0006665 sphingolipid metabolic process 2
GO:0006839 mitochondrial transport 1
GO:0000742 karyogamy during conjugation with cellular fusion 1
GO:0006898 receptor-mediated endocytosis 1
GO:0006661 phosphatidylinositol biosynthetic process 1
GO:0007047 cell wall organization 3
GO:0007015 actin filament organization 1
GO:0001403 invasive growth in response to glucose limitation 1
GO:0042026 protein refolding 1
GO:0009061 anaerobic respiration 2
GO:0009408 response to heat 1
GO:0009060 aerobic respiration 1
GO:0007010 cytoskeleton organization 1
GO:0006783 heme biosynthetic process 2
GO:0055085 transmembrane transport 1
GO:0007243 protein kinase cascade 1
GO:0019722 calcium-mediated signaling 2
GO:0000754 adaptation to pheromone during conjugation with cellular fusion 3
GO:0016237 microautophagy 1
GO:0007165 signal transduction 1
GO:0042144 vacuole fusion, non-autophagic 1
GO:0006873 cellular ion homeostasis 3
GO:0006457 protein folding 1
GO:0034605 cellular response to heat 2
GO:0015867 ATP transport 1
GO:0015866 ADP transport 1
GO:0042991 transcription factor import into nucleus 1

00720|Reductive carboxylate cycle (CO2 fixation)|11|21
GO:0006094 gluconeogenesis 1
GO:0019654 acetate fermentation 1
GO:0000002 mitochondrial genome maintenance 1
GO:0042493 response to drug 1
GO:0019541 propionate metabolic process 1
GO:0006740 NADPH regeneration 1
GO:0006635 fatty acid beta-oxidation 2
GO:0006108 malate metabolic process 2
GO:0006106 fumarate metabolic process 1
GO:0009060 aerobic respiration 1
GO:0006102 isocitrate metabolic process 3
GO:0006101 citrate metabolic process 1

GO:0006085 acetyl-CoA biosynthetic process 2
GO:0006537 glutamate biosynthetic process 3
GO:0016573 histone acetylation 2
GO:0006735 NADH regeneration 1
GO:0001302 replicative cell aging 1
GO:0006099 tricarboxylic acid cycle 3
GO:0006097 glyoxylate cycle 1
GO:0001300 chronological cell aging 1
GO:0016558 protein import into peroxisome matrix 1

00120|Bile acid biosynthesis|22|23
GO:0006090 pyruvate metabolic process 1
GO:0006979 response to oxidative stress 3
GO:0006741 NADP biosynthetic process 1
GO:0006635 fatty acid beta-oxidation 1
GO:0006740 NADPH regeneration 1
GO:0019388 galactose catabolic process 1
GO:0000947 amino acid catabolic process to alcohol via Ehrlich pathway 6
GO:0008204 ergosterol metabolic process 1
GO:0033859 furaldehyde metabolic process 1
GO:0009651 response to salt stress 2
GO:0046294 formaldehyde catabolic process 1
GO:0019413 acetate biosynthetic process 3
GO:0042843 D-xylose catabolic process 3
GO:0009636 response to toxin 5
GO:0006950 response to stress 1
GO:0019568 arabinose catabolic process 3
GO:0016125 sterol metabolic process 1
GO:0006116 NADH oxidation 4
GO:0034605 cellular response to heat 2
GO:0043458 ethanol biosynthetic process during fermentation 2
GO:0006113 fermentation 1
GO:0006067 ethanol metabolic process 2
GO:0006066 alcohol metabolic process 1

03020|RNA polymerase|29|13
GO:0034402 recruitment of 3'-end processing factors to RNA polymerase II holoenzyme complex 1
GO:0042790 transcription of nuclear rRNA large RNA polymerase I transcript 3
GO:0006368 RNA elongation from RNA polymerase II promoter 1
GO:0042493 response to drug 1
GO:0006366 transcription from RNA polymerase II promoter 12
GO:0006974 response to DNA damage stimulus 1
GO:0006283 transcription-coupled nucleotide-excision repair 1
GO:0006406 mRNA export from nucleus 1
GO:0006384 transcription initiation from RNA polymerase III promoter 1
GO:0000291 nuclear-transcribed mRNA catabolic process, exonucleolytic 1
GO:0000288 nuclear-transcribed mRNA catabolic process, deadenylation-dependent decay 1
GO:0060213 positive regulation of nuclear-transcribed mRNA poly(A) tail shortening 1
GO:0008361 regulation of cell size 2

00624|1- and 2-Methylnaphthalene degradation|9|9
GO:0006635 fatty acid beta-oxidation 1
GO:0033859 furaldehyde metabolic process 1
GO:0043458 ethanol biosynthetic process during fermentation 2

GO:0006474 N-terminal protein amino acid acetylation 1
GO:0006116 NADH oxidation 4
GO:0006067 ethanol metabolic process 1
GO:0006113 fermentation 1
GO:0046294 formaldehyde catabolic process 1
GO:0000947 amino acid catabolic process to alcohol via Ehrlich pathway 6

00260|Glycine, serine and threonine metabolism|42|62
GO:0009651 response to salt stress 1
GO:0019388 galactose catabolic process 1
GO:0006646 phosphatidylethanolamine biosynthetic process 1
GO:0009071 serine family amino acid catabolic process 1
GO:0009070 serine family amino acid biosynthetic process 4
GO:0006066 alcohol metabolic process 1
GO:0006353 transcription termination 1
GO:0042843 D-xylose catabolic process 3
GO:0006549 isoleucine metabolic process 1
GO:0045144 meiotic sister chromatid segregation 1
GO:0006546 glycine catabolic process 2
GO:0006090 pyruvate metabolic process 1
GO:0006897 endocytosis 1
GO:0006545 glycine biosynthetic process 1
GO:0006544 glycine metabolic process 1
GO:0006574 valine catabolic process 1
GO:0042743 hydrogen peroxide metabolic process 1
GO:0006412 translation 1
GO:0006730 one-carbon metabolic process 4
GO:0009097 isoleucine biosynthetic process 1
GO:0034453 microtubule anchoring 1
GO:0019346 transsulfuration 2
GO:0019344 cysteine biosynthetic process 1
GO:0006635 fatty acid beta-oxidation 1
GO:0009060 aerobic respiration 1
GO:0009092 homoserine metabolic process 1
GO:0019343 cysteine biosynthetic process via cystathionine 2
GO:0006312 mitotic recombination 1
GO:0006950 response to stress 1
GO:0070814 hydrogen sulfide biosynthetic process 1
GO:0019568 arabinose catabolic process 3
GO:0006535 cysteine biosynthetic process from serine 1
GO:0006567 threonine catabolic process 2
GO:0006534 cysteine metabolic process 1
GO:0006566 threonine metabolic process 3
GO:0006564 L-serine biosynthetic process 3
GO:0001403 invasive growth in response to glucose limitation 1
GO:0045931 positive regulation of mitotic cell cycle 1
GO:0009636 response to toxin 5
GO:0019464 glycine decarboxylation via glycine cleavage system 1
GO:0006435 threonyl-tRNA aminoacylation 1
GO:0006434 seryl-tRNA aminoacylation 1
GO:0000096 sulfur amino acid metabolic process 1
GO:0009088 threonine biosynthetic process 1
GO:0006783 heme biosynthetic process 1
GO:0006659 phosphatidylserine biosynthetic process 1
GO:0006979 response to oxidative stress 3
GO:0007124 pseudohyphal growth 1
GO:0034605 cellular response to heat 1
GO:0006656 phosphatidylcholine biosynthetic process 4
GO:0006555 methionine metabolic process 4
GO:0009113 purine base biosynthetic process 1

GO:0007020 microtubule nucleation 1
GO:0006552 leucine catabolic process 1
GO:0070159 mitochondrial threonyl-tRNA aminoacylation 1
GO:0006550 isoleucine catabolic process 1
GO:0070158 mitochondrial seryl-tRNA aminoacylation 1
GO:0006426 glycyl-tRNA aminoacylation 1
GO:0042493 response to drug 5
GO:0006103 2-oxoglutarate metabolic process 1
GO:0051315 attachment of spindle microtubules to kinetochore during mitosis 1
GO:0070150 mitochondrial glycyl-tRNA aminoacylation 1

01040|Biosynthesis of unsaturated fatty acids|10|15
GO:0006635 fatty acid beta-oxidation 1
GO:0006633 fatty acid biosynthetic process 1
GO:0000038 very-long-chain fatty acid metabolic process 1
GO:0016579 protein deubiquitination 1
GO:0034605 cellular response to heat 1
GO:0019368 fatty acid elongation, unsaturated fatty acid 1
GO:0019395 fatty acid oxidation 1
GO:0007034 vacuolar transport 1
GO:0006892 post-Golgi vesicle-mediated transport 1
GO:0000001 mitochondrion inheritance 1
GO:0030497 fatty acid elongation 3
GO:0030148 sphingolipid biosynthetic process 3
GO:0016192 vesicle-mediated transport 1
GO:0045449 regulation of transcription 1
GO:0006636 unsaturated fatty acid biosynthetic process 1

00620|Pyruvate metabolism|33|48
GO:0006740 NADPH regeneration 1
GO:0009651 response to salt stress 1
GO:0019388 galactose catabolic process 1
GO:0006998 nuclear envelope organization 1
GO:0006067 ethanol metabolic process 1
GO:0006099 tricarboxylic acid cycle 1
GO:0006097 glyoxylate cycle 1
GO:0006096 glycolysis 1
GO:0042843 D-xylose catabolic process 1
GO:0006094 gluconeogenesis 4
GO:0045144 meiotic sister chromatid segregation 1
GO:0006546 glycine catabolic process 1
GO:0006090 pyruvate metabolic process 8
GO:0006574 valine catabolic process 1
GO:0042743 hydrogen peroxide metabolic process 1
GO:0019413 acetate biosynthetic process 3
GO:0006735 NADH regeneration 1
GO:0005975 carbohydrate metabolic process 3
GO:0034453 microtubule anchoring 1
GO:0006606 protein import into nucleus 1
GO:0009060 aerobic respiration 2
GO:0006635 fatty acid beta-oxidation 1
GO:0006089 lactate metabolic process 1
GO:0006312 mitotic recombination 1
GO:0006950 response to stress 1
GO:0019568 arabinose catabolic process 1
GO:0006085 acetyl-CoA biosynthetic process 2
GO:0006083 acetate metabolic process 1
GO:0019243 methylglyoxal catabolic process to D-lactate 3
GO:0006564 L-serine biosynthetic process 1
GO:0016558 protein import into peroxisome matrix 1

GO:0045931 positive regulation of mitotic cell cycle 1
GO:0001302 replicative cell aging 1
GO:0006979 response to oxidative stress 1
GO:0019654 acetate fermentation 1
GO:0001300 chronological cell aging 1
GO:0034605 cellular response to heat 6
GO:0019878 lysine biosynthetic process via aminoadipic acid 2
GO:0007020 microtubule nucleation 1
GO:0006552 leucine catabolic process 1
GO:0006520 cellular amino acid metabolic process 1
GO:0006550 isoleucine catabolic process 1
GO:0006749 glutathione metabolic process 1
GO:0006108 malate metabolic process 2
GO:0016573 histone acetylation 2
GO:0051315 attachment of spindle microtubules to kinetochore during mitosis 1
GO:0006103 2-oxoglutarate metabolic process 1
GO:0006741 NADP biosynthetic process 1

00520|Nucleotide sugars metabolism|4|6
GO:0005992 trehalose biosynthetic process 1
GO:0006486 protein amino acid glycosylation 1
GO:0005978 glycogen biosynthetic process 1
GO:0006078 1,6-beta-glucan biosynthetic process 1
GO:0033499 galactose catabolic process via UDP-galactose 2
GO:0006011 UDP-glucose metabolic process 1

03420|Nucleotide excision repair|34|55
GO:0000082 G1/S transition of mitotic cell cycle 1
GO:0000278 mitotic cell cycle 2
GO:0010525 regulation of transposition, RNA-mediated 2
GO:0000727 double-strand break repair via break-induced replication 1
GO:0000724 double-strand break repair via homologous recombination 2
GO:0000722 telomere maintenance via recombination 2
GO:0006289 nucleotide-excision repair 18
GO:0045944 positive regulation of transcription from RNA polymerase II promoter 1
GO:0006284 base-excision repair 5
GO:0016567 protein ubiquitination 2
GO:0000019 regulation of mitotic recombination 3
GO:0006283 transcription-coupled nucleotide-excision repair 2
GO:0007070 negative regulation of transcription from RNA polymerase II promoter, mitotic 6
GO:0006281 DNA repair 3
GO:0000075 cell cycle checkpoint 1
GO:0030466 chromatin silencing at silent mating-type cassette 1
GO:0030433 ER-associated protein catabolic process 1
GO:0006348 chromatin silencing at telomere 4
GO:0007131 reciprocal meiotic recombination 2
GO:0030491 heteroduplex formation 2
GO:0006312 mitotic recombination 4
GO:0000717 nucleotide-excision repair, DNA duplex unwinding 4
GO:0006310 DNA recombination 1
GO:0000715 nucleotide-excision repair, DNA damage recognition 3
GO:0016973 poly(A)+ mRNA export from nucleus 1
GO:0007004 telomere maintenance via telomerase 2
GO:0006278 RNA-dependent DNA replication 3
GO:0000710 meiotic mismatch repair 3
GO:0007064 mitotic sister chromatid cohesion 2

GO:0007062 sister chromatid cohesion   4
GO:0045005 maintenance of fidelity during DNA-dependent DNA replication
      1
GO:0006273 lagging strand elongation   9
GO:0006272 leading strand elongation   13
GO:0006468 protein amino acid phosphorylation 1
GO:0043137 DNA replication, removal of RNA primer   3
GO:0033683 nucleotide-excision repair, DNA incision 2
GO:0006303 double-strand break repair via nonhomologous end joining
      1
GO:0006367 transcription initiation from RNA polymerase II promoter
      9
GO:0006366 transcription from RNA polymerase II promoter   2
GO:0031146 SCF-dependent proteasomal ubiquitin-dependent protein catabolic process 1
GO:0006301 postreplication repair 4
GO:0000736 double-strand break repair via single-strand annealing, removal of nonhomologous ends 2
GO:0000735 removal of nonhomologous ends 2
GO:0045184 establishment of protein localization   2
GO:0006268 DNA unwinding during replication   2
GO:0000731 DNA synthesis during DNA repair   1
GO:0006266 DNA ligation   1
GO:0006298 mismatch repair   12
GO:0033260 DNA replication during S phase   1
GO:0042787 protein ubiquitination during ubiquitin-dependent protein catabolic process 1
GO:0006296 nucleotide-excision repair, DNA incision, 5'-to lesion
      2
GO:0006295 nucleotide-excision repair, DNA incision, 3'-to lesion
      1
GO:0042177 negative regulation of protein catabolic process   1
GO:0006260 DNA replication   3
GO:0000086 G2/M transition of mitotic cell cycle   1

00030|Pentose phosphate pathway|28|24
GO:0042542 response to hydrogen peroxide 1
GO:0006094 gluconeogenesis   3
GO:0008361 regulation of cell size 1
GO:0006409 tRNA export from nucleus   2
GO:0005978 glycogen biosynthetic process 2
GO:0009051 pentose-phosphate shunt, oxidative branch 5
GO:0006979 response to oxidative stress 1
GO:0006740 NADPH regeneration   1
GO:0006015 5-phosphoribose 1-diphosphate biosynthetic process   5
GO:0019388 galactose catabolic process   2
GO:0006011 UDP-glucose metabolic process 2
GO:0006800 oxygen and reactive oxygen species metabolic process 1
GO:0015992 proton transport 2
GO:0019521 D-gluconate metabolic process 1
GO:0045013 negative regulation of transcription by carbon catabolites
      1
GO:0006874 cellular calcium ion homeostasis   1
GO:0031505 fungal-type cell wall organization 5
GO:0034605 cellular response to heat   2
GO:0006008 glucose 1-phosphate utilization   2
GO:0005992 trehalose biosynthetic process   2
GO:0008615 pyridoxine biosynthetic process   1
GO:0006098 pentose-phosphate shunt 6
GO:0030003 cellular cation homeostasis   1
GO:0006096 glycolysis 4

00730|Thiamine metabolism|5|7
GO:0006879 cellular iron ion homeostasis 1
GO:0016226 iron-sulfur cluster assembly 1
GO:0009230 thiamin catabolic process   1
GO:0009229 thiamin diphosphate biosynthetic process 1
GO:0002098 tRNA wobble uridine modification   1
GO:0009228 thiamin biosynthetic process 3
GO:0034227 tRNA thio-modification 1

00130|Ubiquinone and menaquinone biosynthesis|6|4
GO:0034605 cellular response to heat   1
GO:0006744 ubiquinone biosynthetic process   5
GO:0019439 aromatic compound catabolic process 1
GO:0009060 aerobic respiration   1

03050|Proteasome|35|11
GO:0043248 proteasome assembly   7
GO:0006950 response to stress   3
GO:0030163 protein catabolic process   1
GO:0006974 response to DNA damage stimulus   1
GO:0051726 regulation of cell cycle   1
GO:0030447 filamentous growth   2
GO:0043161 proteasomal ubiquitin-dependent protein catabolic process
      1
GO:0006406 mRNA export from nucleus   1
GO:0006887 exocytosis 1
GO:0006511 ubiquitin-dependent protein catabolic process   34
GO:0030437 ascospore formation   3

00670|One carbon pool by folate|14|19
GO:0006546 glycine catabolic process   1
GO:0030437 ascospore formation   1
GO:0006544 glycine metabolic process   1
GO:0009396 folic acid and derivative biosynthetic process 2
GO:0009086 methionine biosynthetic process   1
GO:0006189 'de novo' IMP biosynthetic process 2
GO:0046084 adenine biosynthetic process 1
GO:0006431 methionyl-tRNA aminoacylation 1
GO:0009113 purine base biosynthetic process   2
GO:0001718 conversion of met-tRNAf to fmet-tRNA   1
GO:0046656 folic acid biosynthetic process   1
GO:0006139 nucleobase, nucleoside, nucleotide and nucleic acid
metabolic process 1
GO:0009060 aerobic respiration   1
GO:0006413 translational initiation   1
GO:0006164 purine nucleotide biosynthetic process   2
GO:0034605 cellular response to heat   1
GO:0009070 serine family amino acid biosynthetic process   1
GO:0006730 one-carbon metabolic process 4
GO:0006760 folic acid and derivative metabolic process   1

00362|Benzoate degradation via hydroxylation|2|1
GO:0006635 fatty acid beta-oxidation   1

00361|gamma-Hexachlorocyclohexane degradation|10|8
GO:0007124 pseudohyphal growth   1
GO:0008361 regulation of cell size 1
GO:0030476 ascospore wall assembly 1
GO:0006796 phosphate metabolic process   3
GO:0016036 cellular response to phosphate starvation 1

GO:0001403 invasive growth in response to glucose limitation   1
GO:0006470 protein amino acid dephosphorylation   3
GO:0042723 thiamin and derivative metabolic process 1

00360|Phenylalanine metabolism|8|8
GO:0006474 N-terminal protein amino acid acetylation 1
GO:0019483 beta-alanine biosynthetic process   2
GO:0006532 aspartate biosynthetic process   1
GO:0006950 response to stress   1
GO:0000105 histidine biosynthetic process   1
GO:0006598 polyamine catabolic process   2
GO:0001302 replicative cell aging 1
GO:0001300 chronological cell aging   1

00530|Aminosugars metabolism|15|20
GO:0007114 cell budding   1
GO:0008361 regulation of cell size 1
GO:0006032 chitin catabolic process   1
GO:0006000 fructose metabolic process   2
GO:0006979 response to oxidative stress 1
GO:0006048 UDP-N-acetylglucosamine biosynthetic process   2
GO:0006696 ergosterol biosynthetic process   1
GO:0006013 mannose metabolic process   3
GO:0000916 contractile ring contraction involved in cytokinesis 1
GO:0030476 ascospore wall assembly 3
GO:0000910 cytokinesis 2
GO:0007109 cytokinesis, completion of separation   2
GO:0046015 regulation of transcription by glucose   1
GO:0032445 fructose import   2
GO:0046323 glucose import   3
GO:0034605 cellular response to heat   4
GO:0006038 cell wall chitin biosynthetic process   3
GO:0001302 replicative cell aging 1
GO:0006006 glucose metabolic process   3
GO:0006096 glycolysis 3

00230|Purine metabolism|89|72
GO:0006384 transcription initiation from RNA polymerase III promoter
      1
GO:0006289 nucleotide-excision repair   6
GO:0043137 DNA replication, removal of RNA primer   3
GO:0006284 base-excision repair   3
GO:0006189 'de novo' IMP biosynthetic process 5
GO:0006283 transcription-coupled nucleotide-excision repair   1
GO:0030437 ascospore formation   1
GO:0007188 G-protein signaling, coupled to cAMP nucleotide second
messenger   1
GO:0006183 GTP biosynthetic process   2
GO:0046037 GMP metabolic process   2
GO:0006279 premeiotic DNA synthesis   1
GO:0006278 RNA-dependent DNA replication 4
GO:0006273 lagging strand elongation   11
GO:0006272 leading strand elongation   7
GO:0000103 sulfate assimilation   2
GO:0006270 DNA replication initiation   4
GO:0006015 5-phosphoribose 1-diphosphate biosynthetic process   5
GO:0006172 ADP biosynthetic process   1
GO:0006406 mRNA export from nucleus   1
GO:0009117 nucleotide metabolic process 4
GO:0006303 double-strand break repair via nonhomologous end joining
      1

GO:0006368 RNA elongation from RNA polymerase II promoter 1
GO:0006301 postreplication repair 3
GO:0006366 transcription from RNA polymerase II promoter   12
GO:0006269 DNA replication, synthesis of RNA primer 2
GO:0046084 adenine biosynthetic process 1
GO:0000256 allantoin catabolic process   2
GO:0019358 nicotinate nucleotide salvage 1
GO:0006168 adenine salvage   1
GO:0006167 AMP biosynthetic process   1
GO:0006260 DNA replication   4
GO:0007265 Ras protein signal transduction   1
GO:0006165 nucleoside diphosphate phosphorylation   1
GO:0006164 purine nucleotide biosynthetic process   6
GO:0008361 regulation of cell size 3
GO:0006163 purine nucleotide metabolic process 2
GO:0006555 methionine metabolic process 2
GO:0046115 guanosine catabolic process   1
GO:0009263 deoxyribonucleotide biosynthetic process 4
GO:0007064 mitotic sister chromatid cohesion   1
GO:0009060 aerobic respiration   1
GO:0019933 cAMP-mediated signaling 2
GO:0043103 hypoxanthine salvage   1
GO:0031505 fungal-type cell wall organization 5
GO:0000731 DNA synthesis during DNA repair   3
GO:0043101 purine salvage   1
GO:0034605 cellular response to heat   2
GO:0045005 maintenance of fidelity during DNA-dependent DNA replication 1
GO:0006348 chromatin silencing at telomere   3
GO:0019693 ribose phosphate metabolic process 1
GO:0034402 recruitment of 3'-end processing factors to RNA polymerase II holoenzyme complex   1
GO:0016233 telomere capping 1
GO:0000727 double-strand break repair via break-induced replication 1
GO:0006148 inosine catabolic process   1
GO:0006798 polyphosphate catabolic process   1
GO:0006146 adenine catabolic process   1
GO:0009442 allantoin assimilation pathway   1
GO:0006144 purine base metabolic process 3
GO:0000291 nuclear-transcribed mRNA catabolic process, exonucleolytic 1
GO:0033260 DNA replication during S phase   1
GO:0009142 nucleoside triphosphate biosynthetic process   1
GO:0006298 mismatch repair   6
GO:0000288 nuclear-transcribed mRNA catabolic process, deadenylation-dependent decay   1
GO:0034356 NAD biosynthesis via nicotinamide riboside salvage pathway 1
GO:0042790 transcription of nuclear rRNA large RNA polymerase I transcript 3
GO:0060213 positive regulation of nuclear-transcribed mRNA poly(A) tail shortening 1
GO:0006096 glycolysis 1
GO:0042493 response to drug 3
GO:0006090 pyruvate metabolic process   2
GO:0006486 protein amino acid glycosylation   1
GO:0051188 cofactor biosynthetic process 1
GO:0006974 response to DNA damage stimulus   2

03430|Mismatch repair|18|35

GO:0006312 mitotic recombination   4
GO:0006311 meiotic gene conversion 1
GO:0006268 DNA unwinding during replication   2
GO:0006298 mismatch repair   14
GO:0006310 DNA recombination 3
GO:0006266 DNA ligation   1
GO:0007004 telomere maintenance via telomerase 2
GO:0030466 chromatin silencing at silent mating-type cassette   2
GO:0007064 mitotic sister chromatid cohesion   1
GO:0007062 sister chromatid cohesion   4
GO:0006260 DNA replication   3
GO:0045005 maintenance of fidelity during DNA-dependent DNA replication
1
GO:0030491 heteroduplex formation 2
GO:0031292 gene conversion at mating-type locus, DNA double-strand
break processing 1
GO:0016567 protein ubiquitination 2
GO:0006278 RNA-dependent DNA replication 3
GO:0000710 meiotic mismatch repair 4
GO:0000075 cell cycle checkpoint   1
GO:0006273 lagging strand elongation   5
GO:0000278 mitotic cell cycle   2
GO:0043137 DNA replication, removal of RNA primer   3
GO:0006272 leading strand elongation   9
GO:0000729 DNA double-strand break processing 1
GO:0000727 double-strand break repair via break-induced replication
1
GO:0045184 establishment of protein localization   2
GO:0000724 double-strand break repair via homologous recombination
2
GO:0000723 telomere maintenance   1
GO:0000722 telomere maintenance via recombination   2
GO:0006301 postreplication repair 4
GO:0006289 nucleotide-excision repair   7
GO:0007131 reciprocal meiotic recombination   3
GO:0006284 base-excision repair   5
GO:0006281 DNA repair 3
GO:0006348 chromatin silencing at telomere   1
GO:0000735 removal of nonhomologous ends 2

00040|Pentose and glucuronate interconversions|7|16
GO:0007124 pseudohyphal growth   1
GO:0006979 response to oxidative stress 1
GO:0006078 1,6-beta-glucan biosynthetic process   1
GO:0006950 response to stress   1
GO:0034605 cellular response to heat   1
GO:0005978 glycogen biosynthetic process 1
GO:0006098 pentose-phosphate shunt 1
GO:0006011 UDP-glucose metabolic process 1
GO:0045490 pectin catabolic process   1
GO:0019388 galactose catabolic process   1
GO:0005999 xylulose biosynthetic process 1
GO:0005998 xylulose catabolic process   1
GO:0019568 arabinose catabolic process   1
GO:0006486 protein amino acid glycosylation   1
GO:0042843 D-xylose catabolic process   1
GO:0005992 trehalose biosynthetic process   1

04070|Phosphatidylinositol signaling system|15|24
GO:0007114 cell budding   1
GO:0051300 spindle pole body organization   1

GO:0009395 phospholipid catabolic process 1
GO:0006468 protein amino acid phosphorylation 2
GO:0006607 NLS-bearing substrate import into nucleus 1
GO:0000742 karyogamy during conjugation with cellular fusion 1
GO:0034501 protein localization to kinetochore 1
GO:0007049 cell cycle 1
GO:0006898 receptor-mediated endocytosis 1
GO:0006661 phosphatidylinositol biosynthetic process 2
GO:0007047 cell wall organization 1
GO:0007015 actin filament organization 1
GO:0031321 ascospore-type prospore formation 1
GO:0001402 signal transduction during filamentous growth 1
GO:0046856 phosphoinositide dephosphorylation 3
GO:0046855 inositol phosphate dephosphorylation 2
GO:0007010 cytoskeleton organization 1
GO:0046854 phosphoinositide phosphorylation 5
GO:0007243 protein kinase cascade 1
GO:0016237 microautophagy 1
GO:0007165 signal transduction 1
GO:0042144 vacuole fusion, non-autophagic 1
GO:0032958 inositol phosphate biosynthetic process 1
GO:0042991 transcription factor import into nucleus 1

00740|Riboflavin metabolism|13|12
GO:0001403 invasive growth in response to glucose limitation 1
GO:0007124 pseudohyphal growth 1
GO:0009060 aerobic respiration 1
GO:0006796 phosphate metabolic process 3
GO:0042493 response to drug 1
GO:0009398 FMN biosynthetic process 1
GO:0042723 thiamin and derivative metabolic process 1
GO:0006470 protein amino acid dephosphorylation 1
GO:0009231 riboflavin biosynthetic process 5
GO:0016036 cellular response to phosphate starvation 1
GO:0006747 FAD biosynthetic process 1
GO:0008361 regulation of cell size 1

00400|Phenylalanine, tyrosine and tryptophan biosynthesis|20|11
GO:0070156 mitochondrial phenylalanyl-tRNA aminoacylation 1
GO:0006437 tyrosyl-tRNA aminoacylation 2
GO:0000105 histidine biosynthetic process 1
GO:0000162 tryptophan biosynthetic process 5
GO:0006570 tyrosine metabolic process 1
GO:0009073 aromatic amino acid family biosynthetic process 1
GO:0006432 phenylalanyl-tRNA aminoacylation 3
GO:0001302 replicative cell aging 1
GO:0009094 L-phenylalanine biosynthetic process 1
GO:0001300 chronological cell aging 1
GO:0006532 aspartate biosynthetic process 1

00680|Methane metabolism|8|16
GO:0033859 furaldehyde metabolic process 1
GO:0000302 response to reactive oxygen species 2
GO:0009086 methionine biosynthetic process 1
GO:0006950 response to stress 1
GO:0006735 NADH regeneration 1
GO:0034605 cellular response to heat 1
GO:0001315 age-dependent response to reactive oxygen species 1
GO:0006730 one-carbon metabolic process 2
GO:0042744 hydrogen peroxide catabolic process 1
GO:0009409 response to cold 1

GO:0006800 oxygen and reactive oxygen species metabolic process 1
GO:0009070 serine family amino acid biosynthetic process 1
GO:0046294 formaldehyde catabolic process 1
GO:0000947 amino acid catabolic process to alcohol via Ehrlich pathway 1
GO:0042631 cellular response to water deprivation 1
GO:0042183 formate catabolic process 2

00350|Tyrosine metabolism|18|23
GO:0006540 glutamate decarboxylation to succinate 1
GO:0006979 response to oxidative stress 1
GO:0006635 fatty acid beta-oxidation 1
GO:0000466 maturation of 5.8S rRNA from tricistronic rRNA transcript (SSU-rRNA, 5.8S rRNA, LSU-rRNA) 1
GO:0000154 rRNA modification 1
GO:0000463 maturation of LSU-rRNA from tricistronic rRNA transcript (SSU-rRNA, 5.8S rRNA, LSU-rRNA) 1
GO:0000947 amino acid catabolic process to alcohol via Ehrlich pathway 6
GO:0033859 furaldehyde metabolic process 1
GO:0009450 gamma-aminobutyric acid catabolic process 1
GO:0006474 N-terminal protein amino acid acetylation 1
GO:0046294 formaldehyde catabolic process 1
GO:0031167 rRNA methylation 1
GO:0000105 histidine biosynthetic process 1
GO:0006598 polyamine catabolic process 2
GO:0006950 response to stress 1
GO:0006532 aspartate biosynthetic process 1
GO:0006116 NADH oxidation 4
GO:0043458 ethanol biosynthetic process during fermentation 2
GO:0006113 fermentation 1
GO:0001302 replicative cell aging 1
GO:0006067 ethanol metabolic process 1
GO:0001300 chronological cell aging 1
GO:0019483 beta-alanine biosynthetic process 2

00600|Sphingolipid metabolism|13|10
GO:0009267 cellular response to starvation 1
GO:0046513 ceramide biosynthetic process 2
GO:0034605 cellular response to heat 1
GO:0009408 response to heat 2
GO:0006672 ceramide metabolic process 2
GO:0030148 sphingolipid biosynthetic process 5
GO:0001302 replicative cell aging 2
GO:0019722 calcium-mediated signaling 4
GO:0006666 3-keto-sphinganine metabolic process 1
GO:0006665 sphingolipid metabolic process 4

00240|Pyrimidine metabolism|69|69
GO:0019358 nicotinate nucleotide salvage 1
GO:0006384 transcription initiation from RNA polymerase III promoter 1
GO:0000727 double-strand break repair via break-induced replication 1
GO:0009263 deoxyribonucleotide biosynthetic process 4
GO:0006227 dUDP biosynthetic process 1
GO:0006289 nucleotide-excision repair 6
GO:0006222 UMP biosynthetic process 1
GO:0006541 glutamine metabolic process 1
GO:0006221 pyrimidine nucleotide biosynthetic process 2
GO:0006284 base-excision repair 3

GO:0006283 transcription-coupled nucleotide-excision repair 1
GO:0006281 DNA repair 1
GO:0046087 cytidine metabolic process 1
GO:0006348 chromatin silencing at telomere 3
GO:0060213 positive regulation of nuclear-transcribed mRNA poly(A) tail shortening 1
GO:0034356 NAD biosynthesis via nicotinamide riboside salvage pathway 1
GO:0019858 cytosine metabolic process 1
GO:0019856 pyrimidine base biosynthetic process 2
GO:0000291 nuclear-transcribed mRNA catabolic process, exonucleolytic 1
GO:0045454 cell redox homeostasis 2
GO:0006217 deoxycytidine catabolic process 1
GO:0006216 cytidine catabolic process 1
GO:0006279 premeiotic DNA synthesis 1
GO:0006278 RNA-dependent DNA replication 4
GO:0046115 guanosine catabolic process 1
GO:0007064 mitotic sister chromatid cohesion 1
GO:0006276 plasmid maintenance 1
GO:0008655 pyrimidine salvage 4
GO:0045005 maintenance of fidelity during DNA-dependent DNA replication 1
GO:0006241 CTP biosynthetic process 2
GO:0008654 phospholipid biosynthetic process 2
GO:0006273 lagging strand elongation 11
GO:0006272 leading strand elongation 7
GO:0006406 mRNA export from nucleus 1
GO:0016233 telomere capping 1
GO:0006270 DNA replication initiation 4
GO:0006148 inosine catabolic process 1
GO:0043137 DNA replication, removal of RNA primer 3
GO:0008361 regulation of cell size 2
GO:0042790 transcription of nuclear rRNA large RNA polymerase I transcript 3
GO:0006979 response to oxidative stress 2
GO:0031119 tRNA pseudouridine synthesis 1
GO:0034605 cellular response to heat 1
GO:0000288 nuclear-transcribed mRNA catabolic process, deadenylation-dependent decay 1
GO:0006368 RNA elongation from RNA polymerase II promoter 1
GO:0006303 double-strand break repair via nonhomologous end joining 1
GO:0006366 transcription from RNA polymerase II promoter 12
GO:0006974 response to DNA damage stimulus 2
GO:0009213 pyrimidine deoxyribonucleoside triphosphate catabolic process 1
GO:0006301 postreplication repair 3
GO:0006207 'de novo' pyrimidine base biosynthetic process 7
GO:0006526 arginine biosynthetic process 2
GO:0006269 DNA replication, synthesis of RNA primer 2
GO:0006235 dTTP biosynthetic process 1
GO:0000731 DNA synthesis during DNA repair 3
GO:0006298 mismatch repair 6
GO:0006233 dTDP biosynthetic process 1
GO:0033260 DNA replication during S phase 1
GO:0051188 cofactor biosynthetic process 1
GO:0009142 nucleoside triphosphate biosynthetic process 1
GO:0046132 pyrimidine ribonucleoside biosynthetic process 2
GO:0045984 negative regulation of pyrimidine base metabolic process 1

GO:0006261 DNA-dependent DNA replication 1
GO:0006139 nucleobase, nucleoside, nucleotide and nucleic acid metabolic process 1
GO:0006260 DNA replication   4
GO:0042493 response to drug 1
GO:0006486 protein amino acid glycosylation   1
GO:0034402 recruitment of 3'-end processing factors to RNA polymerase II holoenzyme complex   1
GO:0006165 nucleoside diphosphate phosphorylation   1

00450|Selenoamino acid metabolism|19|25
GO:0006312 mitotic recombination   1
GO:0019346 transsulfuration 4
GO:0019344 cysteine biosynthetic process 3
GO:0019343 cysteine biosynthetic process via cystathionine 2
GO:0000096 sulfur amino acid metabolic process 1
GO:0009086 methionine biosynthetic process   1
GO:0042493 response to drug 4
GO:0070814 hydrogen sulfide biosynthetic process   1
GO:0006431 methionyl-tRNA aminoacylation 2
GO:0000466 maturation of 5.8S rRNA from tricistronic rRNA transcript (SSU-rRNA, 5.8S rRNA, LSU-rRNA)   1
GO:0016259 selenocysteine metabolic process   1
GO:0000154 rRNA modification 1
GO:0000463 maturation of LSU-rRNA from tricistronic rRNA transcript (SSU-rRNA, 5.8S rRNA, LSU-rRNA)   1
GO:0006556 S-adenosylmethionine biosynthetic process 2
GO:0006555 methionine metabolic process 6
GO:0031167 rRNA methylation 1
GO:0006751 glutathione catabolic process 1
GO:0000103 sulfate assimilation   2
GO:0006878 cellular copper ion homeostasis   1
GO:0009636 response to toxin 1
GO:0006535 cysteine biosynthetic process from serine 1
GO:0042908 xenobiotic transport   1
GO:0006534 cysteine metabolic process   1
GO:0034605 cellular response to heat   1
GO:0006790 sulfur metabolic process   1

03060|Protein export|8|6
GO:0006617 SRP-dependent cotranslational protein targeting to membrane, signal sequence recognition   5
GO:0045047 protein targeting to ER 7
GO:0006614 SRP-dependent cotranslational protein targeting to membrane 2
GO:0032979 protein insertion into mitochondrial membrane from inner side 1
GO:0033615 mitochondrial proton-transporting ATP synthase complex assembly   1
GO:0006465 signal peptide processing   1

00340|Histidine metabolism|16|18
GO:0006090 pyruvate metabolic process   1
GO:0006741 NADP biosynthetic process   1
GO:0006740 NADPH regeneration   1
GO:0000466 maturation of 5.8S rRNA from tricistronic rRNA transcript (SSU-rRNA, 5.8S rRNA, LSU-rRNA)   1
GO:0000154 rRNA modification 1
GO:0000463 maturation of LSU-rRNA from tricistronic rRNA transcript (SSU-rRNA, 5.8S rRNA, LSU-rRNA)   1
GO:0032543 mitochondrial translation   1

GO:0009651 response to salt stress 1
GO:0009127 purine nucleoside monophosphate biosynthetic process 1
GO:0031167 rRNA methylation 1
GO:0000105 histidine biosynthetic process 7
GO:0019413 acetate biosynthetic process 3
GO:0006598 polyamine catabolic process 2
GO:0006950 response to stress 1
GO:0006427 histidyl-tRNA aminoacylation 1
GO:0034605 cellular response to heat 1
GO:0006067 ethanol metabolic process 1
GO:0019483 beta-alanine biosynthetic process 2

00650|Butanoate metabolism|22|21
GO:0006090 pyruvate metabolic process 3
GO:0006540 glutamate decarboxylation to succinate 1
GO:0009082 branched chain family amino acid biosynthetic process 2
GO:0006979 response to oxidative stress 5
GO:0006741 NADP biosynthetic process 1
GO:0006635 fatty acid beta-oxidation 1
GO:0006740 NADPH regeneration 1
GO:0006696 ergosterol biosynthetic process 1
GO:0019388 galactose catabolic process 1
GO:0019740 nitrogen utilization 1
GO:0009450 gamma-aminobutyric acid catabolic process 2
GO:0009651 response to salt stress 2
GO:0019413 acetate biosynthetic process 3
GO:0042843 D-xylose catabolic process 3
GO:0006538 glutamate catabolic process 1
GO:0009636 response to toxin 5
GO:0006950 response to stress 1
GO:0019568 arabinose catabolic process 3
GO:0034605 cellular response to heat 4
GO:0006067 ethanol metabolic process 1
GO:0006066 alcohol metabolic process 1

00750|Vitamin B6 metabolism|8|11
GO:0000282 cellular bud site selection 1
GO:0006631 fatty acid metabolic process 1
GO:0008614 pyridoxine metabolic process 4
GO:0006897 endocytosis 1
GO:0034605 cellular response to heat 1
GO:0006916 anti-apoptosis 1
GO:0042823 pyridoxal phosphate biosynthetic process 1
GO:0009228 thiamin biosynthetic process 4
GO:0009070 serine family amino acid biosynthetic process 1
GO:0006564 L-serine biosynthetic process 1
GO:0009113 purine base biosynthetic process 1

00010|Glycolysis / Gluconeogenesis|47|63
GO:0006740 NADPH regeneration 1
GO:0009651 response to salt stress 1
GO:0032889 regulation of vacuole fusion, non-autophagic 2
GO:0006008 glucose 1-phosphate utilization 2
GO:0019388 galactose catabolic process 2
GO:0006006 glucose metabolic process 3
GO:0006067 ethanol metabolic process 3
GO:0006098 pentose-phosphate shunt 1
GO:0006096 glycolysis 16
GO:0006000 fructose metabolic process 2
GO:0006094 gluconeogenesis 11
GO:0045144 meiotic sister chromatid segregation 1

GO:0006546 glycine catabolic process 1
GO:0006090 pyruvate metabolic process 8
GO:0006800 oxygen and reactive oxygen species metabolic process 3
GO:0006574 valine catabolic process 1
GO:0000949 aromatic amino acid family catabolic process to alcohol via Ehrlich pathway 3
GO:0045944 positive regulation of transcription from RNA polymerase II promoter 1
GO:0042743 hydrogen peroxide metabolic process 1
GO:0000947 amino acid catabolic process to alcohol via Ehrlich pathway 6
GO:0019413 acetate biosynthetic process 3
GO:0005978 glycogen biosynthetic process 2
GO:0034453 microtubule anchoring 1
GO:0009228 thiamin biosynthetic process 1
GO:0033859 furaldehyde metabolic process 1
GO:0006312 mitotic recombination 1
GO:0015992 proton transport 2
GO:0006950 response to stress 1
GO:0006085 acetyl-CoA biosynthetic process 2
GO:0006569 tryptophan catabolic process 3
GO:0006598 polyamine catabolic process 2
GO:0006564 L-serine biosynthetic process 1
GO:0033499 galactose catabolic process via UDP-galactose 1
GO:0045931 positive regulation of mitotic cell cycle 1
GO:0043458 ethanol biosynthetic process during fermentation 2
GO:0006116 NADH oxidation 4
GO:0008361 regulation of cell size 1
GO:0006113 fermentation 1
GO:0046015 regulation of transcription by glucose 1
GO:0006915 apoptosis 2
GO:0019655 glucose catabolic process to ethanol 1
GO:0001302 replicative cell aging 1
GO:0019654 acetate fermentation 1
GO:0005992 trehalose biosynthetic process 2
GO:0034605 cellular response to heat 3
GO:0006013 mannose metabolic process 3
GO:0006011 UDP-glucose metabolic process 2
GO:0030003 cellular cation homeostasis 1
GO:0046294 formaldehyde catabolic process 1
GO:0006559 L-phenylalanine catabolic process 3
GO:0032445 fructose import 2
GO:0006874 cellular calcium ion homeostasis 1
GO:0007020 microtubule nucleation 1
GO:0006552 leucine catabolic process 3
GO:0006550 isoleucine catabolic process 1
GO:0016573 histone acetylation 2
GO:0006103 2-oxoglutarate metabolic process 1
GO:0000951 methionine catabolic process to 3-methylthiopropanol 1
GO:0051315 attachment of spindle microtubules to kinetochore during mitosis 1
GO:0019483 beta-alanine biosynthetic process 2
GO:0000950 branched chain family amino acid catabolic process to alcohol via Ehrlich pathway 2
GO:0006741 NADP biosynthetic process 1
GO:0046323 glucose import 3

04111|Cell cycle - yeast|109|171
GO:0045944 positive regulation of transcription from RNA polymerase II promoter 1
GO:0016558 protein import into peroxisome matrix 1

GO:0030969 UFP-specific transcription factor mRNA processing during unfolded protein response 1
GO:0051726 regulation of cell cycle 4
GO:0045740 positive regulation of DNA replication 2
GO:0030174 regulation of DNA replication initiation 3
GO:0070550 rDNA condensation 3
GO:0042787 protein ubiquitination during ubiquitin-dependent protein catabolic process 4
GO:0043254 regulation of protein complex assembly 1
GO:0007050 cell cycle arrest 1
GO:0008315 meiotic G2/MI transition 1
GO:0045936 negative regulation of phosphate metabolic process 2
GO:0045931 positive regulation of mitotic cell cycle 1
GO:0045930 negative regulation of mitotic cell cycle 1
GO:0006975 DNA damage induced protein phosphorylation 1
GO:0006974 response to DNA damage stimulus 2
GO:0045893 positive regulation of transcription, DNA-dependent 1
GO:0045892 negative regulation of transcription, DNA-dependent 1
GO:0030163 protein catabolic process 1
GO:0007049 cell cycle 1
GO:0050849 negative regulation of calcium-mediated signaling 2
GO:0001403 invasive growth in response to glucose limitation 1
GO:0007047 cell wall organization 1
GO:0051312 chromosome decondensation 1
GO:0008104 protein localization 2
GO:0043433 negative regulation of transcription factor activity 1
GO:0000135 septin checkpoint 2
GO:0031578 mitotic cell cycle spindle orientation checkpoint 3
GO:0031576 G2/M transition checkpoint 1
GO:0031575 G1/S transition checkpoint 1
GO:0031573 intra-S DNA damage checkpoint 2
GO:0032837 distributive segregation 1
GO:0000921 septin ring assembly 2
GO:0031571 G1 DNA damage checkpoint 1
GO:0007035 vacuolar acidification 2
GO:0000725 recombinational repair 2
GO:0000724 double-strand break repair via homologous recombination 2
GO:0007033 vacuole organization 1
GO:0000723 telomere maintenance 1
GO:0000722 telomere maintenance via recombination 2
GO:0043193 positive regulation of gene-specific transcription 3
GO:0045719 negative regulation of glycogen biosynthetic process 1
GO:0000321 re-entry into mitotic cell cycle after pheromone arrest 1
GO:0000320 re-entry into mitotic cell cycle 1
GO:0031569 G2/M transition size control checkpoint 1
GO:0008054 cyclin catabolic process 12
GO:0000122 negative regulation of transcription from RNA polymerase II promoter 2
GO:0016481 negative regulation of transcription 2
GO:0000087 M phase of mitotic cell cycle 1
GO:0000280 nuclear division 1
GO:0000086 G2/M transition of mitotic cell cycle 14
GO:0000910 cytokinesis 2
GO:0000084 S phase of mitotic cell cycle 9
GO:0000083 regulation of transcription of G1/S-phase of mitotic cell cycle 2
GO:0000082 G1/S transition of mitotic cell cycle 12
GO:0006357 regulation of transcription from RNA polymerase II promoter 1

GO:0000712 resolution of meiotic joint molecules as recombinants 1
GO:0045116 protein neddylation 1
GO:0009113 purine base biosynthetic process 1
GO:0000080 G1 phase of mitotic cell cycle 3
GO:0034087 establishment of mitotic sister chromatid cohesion 2
GO:0006350 transcription 3
GO:0000278 mitotic cell cycle 2
GO:0045860 positive regulation of protein kinase activity 1
GO:0000114 regulation of transcription during G1 phase of mitotic cell cycle 2
GO:0000079 regulation of cyclin-dependent protein kinase activity 7
GO:0000902 cell morphogenesis 1
GO:0000078 cell morphogenesis checkpoint 2
GO:0000077 DNA damage checkpoint 6
GO:0000076 DNA replication checkpoint 1
GO:0000075 cell cycle checkpoint 1
GO:0006348 chromatin silencing at telomere 5
GO:0051447 negative regulation of meiotic cell cycle 1
GO:0051446 positive regulation of meiotic cell cycle 1
GO:0000070 mitotic sister chromatid segregation 21
GO:0006343 establishment of chromatin silencing 1
GO:0046686 response to cadmium ion 1
GO:0046685 response to arsenic 1
GO:0042144 vacuole fusion, non-autophagic 1
GO:0043409 negative regulation of MAPKKK cascade 1
GO:0034502 protein localization to chromosome 2
GO:0010032 meiotic chromosome condensation 2
GO:0000105 histidine biosynthetic process 1
GO:0043007 maintenance of rDNA 1
GO:0006338 chromatin remodeling 1
GO:0007000 nucleolus organization 2
GO:0031939 negative regulation of chromatin silencing at telomere 1
GO:0006333 chromatin assembly or disassembly 1
GO:0031146 SCF-dependent proteasomal ubiquitin-dependent protein catabolic process 6
GO:0031938 regulation of chromatin silencing at telomere 4
GO:0006139 nucleobase, nucleoside, nucleotide and nucleic acid metabolic process 2
GO:0031145 anaphase-promoting complex-dependent proteasomal ubiquitin-dependent protein catabolic process 11
GO:0042493 response to drug 2
GO:0009847 spore germination 1
GO:0030472 mitotic spindle organization in nucleus 2
GO:0045840 positive regulation of mitosis 1
GO:0070058 tRNA gene clustering 5
GO:0010571 positive regulation of DNA replication during S phase 1
GO:0010570 regulation of filamentous growth 1
GO:0031134 sister chromatid biorientation 2
GO:0006289 nucleotide-excision repair 2
GO:0051225 spindle assembly 2
GO:0030466 chromatin silencing at silent mating-type cassette 9
GO:0006915 apoptosis 2
GO:0051382 kinetochore assembly 1
GO:0006913 nucleocytoplasmic transport 1
GO:0006281 DNA repair 2
GO:0010569 regulation of double-strand break repair via homologous recombination 1
GO:0010568 regulation of budding cell apical bud growth 1
GO:0034048 negative regulation of protein phosphatase type 2A activity

1
GO:0006473 protein amino acid acetylation 2
GO:0006279 premeiotic DNA synthesis 2
GO:0006310 DNA recombination 1
GO:0006470 protein amino acid dephosphorylation 1
GO:0040020 regulation of meiosis 1
GO:0006271 DNA strand elongation during DNA replication 5
GO:0051177 meiotic sister chromatid cohesion 1
GO:0006270 DNA replication initiation 15
GO:0008361 regulation of cell size 4
GO:0007138 meiotic anaphase II 1
GO:0051568 histone H3-K4 methylation 1
GO:0006468 protein amino acid phosphorylation 13
GO:0016036 cellular response to phosphate starvation 3
GO:0007131 reciprocal meiotic recombination 4
GO:0007130 synaptonemal complex assembly 1
GO:0006302 double-strand break repair 2
GO:0007096 regulation of exit from mitosis 10
GO:0016192 vesicle-mediated transport 1
GO:0006268 DNA unwinding during replication 3
GO:0006461 protein complex assembly 1
GO:0007094 mitotic cell cycle spindle assembly checkpoint 6
GO:0070194 synaptonemal complex disassembly 1
GO:0006267 pre-replicative complex assembly 14
GO:0031110 regulation of microtubule polymerization or depolymerization
1
GO:0034984 cellular response to DNA damage stimulus 1
GO:0007092 activation of anaphase-promoting complex activity during
mitotic cell cycle 2
GO:0007091 mitotic metaphase/anaphase transition 13
GO:0007090 regulation of S phase of mitotic cell cycle 2
GO:0006261 DNA-dependent DNA replication 1
GO:0006260 DNA replication 1
GO:0031505 fungal-type cell wall organization 1
GO:0016584 nucleosome positioning 2
GO:0032297 negative regulation of DNA replication initiation 1
GO:0007127 meiosis I 1
GO:0007126 meiosis 4
GO:0000022 mitotic spindle elongation 13
GO:0000183 chromatin silencing at rDNA 1
GO:0032888 regulation of mitotic spindle elongation 1
GO:0031106 septin ring organization 2
GO:0006259 DNA metabolic process 1
GO:0030437 ascospore formation 1
GO:0032880 regulation of protein localization 2
GO:0010898 positive regulation of triglyceride catabolic process 1
GO:0016572 histone phosphorylation 1
GO:0007117 budding cell bud growth 1
GO:0010697 negative regulation of spindle pole body separation 2
GO:0010696 positive regulation of spindle pole body separation 7
GO:0009202 deoxyribonucleoside triphosphate biosynthetic process 1
GO:0032878 regulation of establishment or maintenance of cell polarity
3
GO:0007076 mitotic chromosome condensation 7
GO:0016567 protein ubiquitination 13
GO:0031647 regulation of protein stability 1
GO:0048478 replication fork protection 1
GO:0006796 phosphate metabolic process 3
GO:0010526 negative regulation of transposition, RNA-mediated 2
GO:0007103 spindle pole body duplication in nuclear envelope 1
GO:0000755 cytogamy 1

GO:0007064 mitotic sister chromatid cohesion   5
GO:0046777 protein amino acid autophosphorylation   2
GO:0000751 cell cycle arrest in response to pheromone   2
GO:0000750 pheromone-dependent signal transduction involved in conjugation with cellular fusion   2

00860|Porphyrin and chlorophyll metabolism|16|10
GO:0009086 methionine biosynthetic process   1
GO:0018063 cytochrome c-heme linkage   2
GO:0042493 response to drug 1
GO:0032543 mitochondrial translation   1
GO:0070149 mitochondrial glutamyl-tRNA aminoacylation   1
GO:0006784 heme a biosynthetic process   2
GO:0006783 heme biosynthetic process   8
GO:0000103 sulfate assimilation   2
GO:0019354 siroheme biosynthetic process 2
GO:0006424 glutamyl-tRNA aminoacylation 1

00290|Valine, leucine and isoleucine biosynthesis|18|15
GO:0009083 branched chain family amino acid catabolic process   2
GO:0070152 mitochondrial isoleucyl-tRNA aminoacylation   1
GO:0009082 branched chain family amino acid biosynthetic process 6
GO:0032543 mitochondrial translation   2
GO:0006412 translation 1
GO:0000002 mitochondrial genome maintenance   1
GO:0006438 valyl-tRNA aminoacylation   1
GO:0000372 Group I intron splicing 1
GO:0006567 threonine catabolic process   1
GO:0009098 leucine biosynthetic process 1
GO:0009071 serine family amino acid catabolic process   1
GO:0009097 isoleucine biosynthetic process   1
GO:0006429 leucyl-tRNA aminoacylation   2
GO:0006428 isoleucyl-tRNA aminoacylation 1
GO:0006090 pyruvate metabolic process   2

00150|Androgen and estrogen metabolism|4|5
GO:0007124 pseudohyphal growth   1
GO:0000466 maturation of 5.8S rRNA from tricistronic rRNA transcript (SSU-rRNA, 5.8S rRNA, LSU-rRNA)   1
GO:0000154 rRNA modification 1
GO:0000463 maturation of LSU-rRNA from tricistronic rRNA transcript (SSU-rRNA, 5.8S rRNA, LSU-rRNA)   1
GO:0031167 rRNA methylation 1

00460|Cyanoamino acid metabolism|6|7
GO:0009070 serine family amino acid biosynthetic process   1
GO:0034605 cellular response to heat   1
GO:0006530 asparagine catabolic process 1
GO:0006995 cellular response to nitrogen starvation 1
GO:0006730 one-carbon metabolic process 2
GO:0006751 glutathione catabolic process 1
GO:0042908 xenobiotic transport   1

00760|Nicotinate and nicotinamide metabolism|7|11
GO:0046115 guanosine catabolic process   1
GO:0034605 cellular response to heat   1
GO:0006148 inosine catabolic process   1
GO:0019358 nicotinate nucleotide salvage 2
GO:0009435 NAD biosynthetic process   1
GO:0019674 NAD metabolic process   2
GO:0001302 replicative cell aging 2

GO:0000183 chromatin silencing at rDNA   2
GO:0006348 chromatin silencing at telomere   2
GO:0034356 NAD biosynthesis via nicotinamide riboside salvage pathway 1
GO:0034354 de novo NAD biosynthetic process from tryptophan   1

00020|Citrate cycle (TCA cycle)|33|38
GO:0006740 NADPH regeneration   1
GO:0006099 tricarboxylic acid cycle   15
GO:0006097 glyoxylate cycle 2
GO:0006094 gluconeogenesis   4
GO:0045144 meiotic sister chromatid segregation   1
GO:0006546 glycine catabolic process   1
GO:0019541 propionate metabolic process 2
GO:0006090 pyruvate metabolic process   4
GO:0006574 valine catabolic process   1
GO:0042743 hydrogen peroxide metabolic process 1
GO:0045333 cellular respiration   5
GO:0006735 NADH regeneration 1
GO:0006121 mitochondrial electron transport, succinate to ubiquinone 4
GO:0034453 microtubule anchoring   1
GO:0009060 aerobic respiration   1
GO:0006635 fatty acid beta-oxidation   2
GO:0006312 mitotic recombination   1
GO:0006537 glutamate biosynthetic process   7
GO:0006564 L-serine biosynthetic process 1
GO:0016558 protein import into peroxisome matrix   1
GO:0045931 positive regulation of mitotic cell cycle 1
GO:0019629 propionate catabolic process, 2-methylcitrate cycle   1
GO:0000002 mitochondrial genome maintenance   1
GO:0001302 replicative cell aging 1
GO:0001300 chronological cell aging   1
GO:0034605 cellular response to heat   2
GO:0007020 microtubule nucleation 1
GO:0006552 leucine catabolic process   1
GO:0006550 isoleucine catabolic process 1
GO:0006108 malate metabolic process   2
GO:0006106 fumarate metabolic process   1
GO:0042493 response to drug 1
GO:0006104 succinyl-CoA metabolic process   2
GO:0051315 attachment of spindle microtubules to kinetochore during mitosis   1
GO:0006103 2-oxoglutarate metabolic process   3
GO:0046356 acetyl-CoA catabolic process 1
GO:0006102 isocitrate metabolic process 5
GO:0006101 citrate metabolic process   4

04120|Ubiquitin mediated proteolysis|43|80
GO:0042787 protein ubiquitination during ubiquitin-dependent protein catabolic process 10
GO:0031146 SCF-dependent proteasomal ubiquitin-dependent protein catabolic process 7
GO:0031145 anaphase-promoting complex-dependent proteasomal ubiquitin-dependent protein catabolic process 11
GO:0000278 mitotic cell cycle   1
GO:0006283 transcription-coupled nucleotide-excision repair   1
GO:0000114 regulation of transcription during G1 phase of mitotic cell cycle 1
GO:0006281 DNA repair 1
GO:0007126 meiosis   1

GO:0016567 protein ubiquitination 17
GO:0030433 ER-associated protein catabolic process   5
GO:0045723 positive regulation of fatty acid biosynthetic process
1
GO:0006513 protein monoubiquitination   9
GO:0045721 negative regulation of gluconeogenesis   1
GO:0051865 protein autoubiquitination   1
GO:0006511 ubiquitin-dependent protein catabolic process   4
GO:0010552 positive regulation of specific transcription from RNA polymerase II promoter 1
GO:0000070 mitotic sister chromatid segregation   13
GO:0006808 regulation of nitrogen utilization 1
GO:0046686 response to cadmium ion 1
GO:0046685 response to arsenic   1
GO:0000209 protein polyubiquitination   11
GO:0032297 negative regulation of DNA replication initiation   1
GO:0032880 regulation of protein localization 1
GO:0000751 cell cycle arrest in response to pheromone   1
GO:0006409 tRNA export from nucleus   1
GO:0006461 protein complex assembly   1
GO:0006301 postreplication repair 2
GO:0006950 response to stress   3
GO:0006364 rRNA processing   1
GO:0045807 positive regulation of endocytosis 1
GO:0010796 regulation of multivesicular body size   1
GO:0010795 regulation of ubiquinone biosynthetic process   1
GO:0010794 regulation of dolichol biosynthetic process   1
GO:0010793 regulation of mRNA export from nucleus   1
GO:0010697 negative regulation of spindle pole body separation   1
GO:0008361 regulation of cell size 2
GO:0045116 protein neddylation   3
GO:0016050 vesicle organization   1
GO:0007005 mitochondrion organization   1
GO:0000055 ribosomal large subunit export from nucleus   1
GO:0032511 late endosome to vacuole transport via multivesicular body sorting pathway   1
GO:0007067 mitosis   1
GO:0034517 ribophagy   1
GO:0030174 regulation of DNA replication initiation 1
GO:0031505 fungal-type cell wall organization 2
GO:0009847 spore germination 1
GO:0043162 ubiquitin-dependent protein catabolic process via the multivesicular body sorting pathway 2
GO:0043161 proteasomal ubiquitin-dependent protein catabolic process
2
GO:0034605 cellular response to heat   1
GO:0016925 protein sumoylation   3
GO:0070086 ubiquitin-dependent endocytosis   1
GO:0032956 regulation of actin cytoskeleton organization   1
GO:0006997 nucleus organization   1
GO:0008054 cyclin catabolic process   12
GO:0000921 septin ring assembly   1
GO:0006897 endocytosis 2
GO:0030163 protein catabolic process   1
GO:0048260 positive regulation of receptor-mediated endocytosis 1
GO:0043254 regulation of protein complex assembly   1
GO:0006986 response to unfolded protein 1
GO:0000910 cytokinesis 1
GO:0006333 chromatin assembly or disassembly   3
GO:0000715 nucleotide-excision repair, DNA damage recognition   1
GO:0016574 histone ubiquitination 1

GO:0019220 regulation of phosphate metabolic process 1
GO:0000022 mitotic spindle elongation 14
GO:0000087 M phase of mitotic cell cycle 1
GO:0000086 G2/M transition of mitotic cell cycle 6
GO:0007035 vacuolar acidification 1
GO:0051382 kinetochore assembly 1
GO:0034644 cellular response to UV 1
GO:0007096 regulation of exit from mitosis 1
GO:0000082 G1/S transition of mitotic cell cycle 7
GO:0006913 nucleocytoplasmic transport 1
GO:0042493 response to drug 1
GO:0032443 regulation of ergosterol biosynthetic process 1
GO:0008033 tRNA processing 1
GO:0007092 activation of anaphase-promoting complex activity during mitotic cell cycle 2
GO:0006974 response to DNA damage stimulus 1
GO:0007091 mitotic metaphase/anaphase transition 13

00562|Inositol phosphate metabolism|15|19
GO:0010526 negative regulation of transposition, RNA-mediated 1
GO:0009395 phospholipid catabolic process 1
GO:0006468 protein amino acid phosphorylation 1
GO:0042493 response to drug 1
GO:0000821 regulation of arginine metabolic process 1
GO:0050821 protein stabilization 1
GO:0000122 negative regulation of transcription from RNA polymerase II promoter 1
GO:0034501 protein localization to kinetochore 1
GO:0007049 cell cycle 1
GO:0006661 phosphatidylinositol biosynthetic process 1
GO:0031321 ascospore-type prospore formation 1
GO:0001402 signal transduction during filamentous growth 1
GO:0046856 phosphoinositide dephosphorylation 3
GO:0046855 inositol phosphate dephosphorylation 2
GO:0046854 phosphoinositide phosphorylation 6
GO:0045944 positive regulation of transcription from RNA polymerase II promoter 1
GO:0006021 inositol biosynthetic process 1
GO:0032958 inositol phosphate biosynthetic process 2
GO:0006096 glycolysis 1

00561|Glycerolipid metabolism|12|20
GO:0046474 glycerophospholipid biosynthetic process 1
GO:0006090 pyruvate metabolic process 1
GO:0042493 response to drug 1
GO:0005975 carbohydrate metabolic process 1
GO:0006979 response to oxidative stress 1
GO:0006741 NADP biosynthetic process 1
GO:0006740 NADPH regeneration 1
GO:0019432 triglyceride biosynthetic process 1
GO:0019388 galactose catabolic process 1
GO:0019915 lipid storage 1
GO:0006071 glycerol metabolic process 1
GO:0006970 response to osmotic stress 2
GO:0009651 response to salt stress 1
GO:0019413 acetate biosynthetic process 3
GO:0042843 D-xylose catabolic process 1
GO:0006950 response to stress 3
GO:0019568 arabinose catabolic process 1
GO:0034605 cellular response to heat 3
GO:0006114 glycerol biosynthetic process 2

GO:0006067 ethanol metabolic process   1

00564|Glycerophospholipid metabolism|23|25
GO:0046475 glycerophospholipid catabolic process   1
GO:0008610 lipid biosynthetic process   1
GO:0046474 glycerophospholipid biosynthetic process 1
GO:0034478 phosphatidylglycerol catabolic process   1
GO:0006887 exocytosis 1
GO:0007006 mitochondrial membrane organization 1
GO:0005975 carbohydrate metabolic process   1
GO:0008654 phospholipid biosynthetic process   1
GO:0006973 intracellular accumulation of glycerol   1
GO:0007049 cell cycle 1
GO:0006661 phosphatidylinositol biosynthetic process 1
GO:0006071 glycerol metabolic process   1
GO:0031321 ascospore-type prospore formation   1
GO:0006474 N-terminal protein amino acid acetylation 1
GO:0006646 phosphatidylethanolamine biosynthetic process   3
GO:0006644 phospholipid metabolic process   1
GO:0000753 cellular morphogenesis during conjugation with cellular fusion   1
GO:0006873 cellular ion homeostasis   1
GO:0034605 cellular response to heat   1
GO:0006116 NADH oxidation   3
GO:0006659 phosphatidylserine biosynthetic process   1
GO:0006657 CDP-choline pathway   1
GO:0001302 replicative cell aging 1
GO:0006656 phosphatidylcholine biosynthetic process 6
GO:0006654 phosphatidic acid biosynthetic process   1

03410|Base excision repair|17|27
GO:0000731 DNA synthesis during DNA repair   1
GO:0006298 mismatch repair   7
GO:0006310 DNA recombination 1
GO:0006266 DNA ligation   1
GO:0030466 chromatin silencing at silent mating-type cassette   1
GO:0007064 mitotic sister chromatid cohesion   2
GO:0006260 DNA replication   1
GO:0045005 maintenance of fidelity during DNA-dependent DNA replication 1
GO:0000710 meiotic mismatch repair 1
GO:0006278 RNA-dependent DNA replication 3
GO:0006273 lagging strand elongation   9
GO:0000278 mitotic cell cycle   1
GO:0006272 leading strand elongation   8
GO:0043137 DNA replication, removal of RNA primer   4
GO:0006307 DNA dealkylation 1
GO:0000727 double-strand break repair via break-induced replication 1
GO:0033260 DNA replication during S phase   1
GO:0006303 double-strand break repair via nonhomologous end joining 2
GO:0006301 postreplication repair 4
GO:0006289 nucleotide-excision repair   8
GO:0006286 base-excision repair, base-free sugar-phosphate removal 1
GO:0006285 base-excision repair, AP site formation   3
GO:0006284 base-excision repair   9
GO:0006281 DNA repair 3
GO:0006348 chromatin silencing at telomere   4
GO:0001302 replicative cell aging 1

GO:0000734 gene conversion at mating-type locus, DNA repair synthesis 1

00563|Glycosylphosphatidylinositol(GPI)-anchor biosynthesis|22|13
GO:0016050 vesicle organization 1
GO:0034605 cellular response to heat 1
GO:0016255 attachment of GPI anchor to protein 4
GO:0015867 ATP transport 1
GO:0006621 protein retention in ER lumen 1
GO:0006888 ER to Golgi vesicle-mediated transport 1
GO:0006276 plasmid maintenance 1
GO:0016485 protein processing 1
GO:0006506 GPI anchor biosynthetic process 15
GO:0006505 GPI anchor metabolic process 1
GO:0007265 Ras protein signal transduction 1
GO:0031505 fungal-type cell wall organization 1
GO:0030433 ER-associated protein catabolic process 2

00565|Ether lipid metabolism|5|6
GO:0006654 phosphatidic acid biosynthetic process 1
GO:0046474 glycerophospholipid biosynthetic process 1
GO:0000753 cellular morphogenesis during conjugation with cellular fusion 1
GO:0006887 exocytosis 1
GO:0006644 phospholipid metabolic process 1
GO:0031321 ascospore-type prospore formation 1

00330|Arginine and proline metabolism|17|21
GO:0019547 arginine catabolic process to ornithine 1
GO:0010133 proline catabolic process to glutamate 2
GO:0070144 mitochondrial arginyl-tRNA aminoacylation 1
GO:0006807 nitrogen compound metabolic process 1
GO:0006527 arginine catabolic process 1
GO:0006526 arginine biosynthetic process 3
GO:0032543 mitochondrial translation 1
GO:0006412 translation 1
GO:0006537 glutamate biosynthetic process 4
GO:0019676 ammonia assimilation cycle 2
GO:0051276 chromosome organization 1
GO:0000053 argininosuccinate metabolic process 1
GO:0006532 aspartate biosynthetic process 1
GO:0006562 proline catabolic process 1
GO:0000052 citrulline metabolic process 1
GO:0034605 cellular response to heat 1
GO:0006561 proline biosynthetic process 1
GO:0006591 ornithine metabolic process 1
GO:0001302 replicative cell aging 1
GO:0006420 arginyl-tRNA aminoacylation 1
GO:0001300 chronological cell aging 1

00630|Glyoxylate and dicarboxylate metabolism|16|23
GO:0006094 gluconeogenesis 1
GO:0000002 mitochondrial genome maintenance 1
GO:0042183 formate catabolic process 2
GO:0042493 response to drug 1
GO:0019541 propionate metabolic process 3
GO:0006635 fatty acid beta-oxidation 1
GO:0009113 purine base biosynthetic process 1
GO:0001718 conversion of met-tRNAf to fmet-tRNA 1
GO:0046656 folic acid biosynthetic process 1
GO:0006108 malate metabolic process 2

GO:0006139 nucleobase, nucleoside, nucleotide and nucleic acid metabolic process 1
GO:0009060 aerobic respiration   1
GO:0006101 citrate metabolic process   4
GO:0006537 glutamate biosynthetic process   3
GO:0006567 threonine catabolic process   1
GO:0046356 acetyl-CoA catabolic process 1
GO:0006735 NADH regeneration 2
GO:0001302 replicative cell aging 1
GO:0006099 tricarboxylic acid cycle   4
GO:0019629 propionate catabolic process, 2-methylcitrate cycle   1
GO:0016558 protein import into peroxisome matrix   1
GO:0001300 chronological cell aging   1
GO:0006097 glyoxylate cycle 3

00983|Drug metabolism - other enzymes|8|7
GO:0006207 'de novo' pyrimidine base biosynthetic process 2
GO:0008655 pyrimidine salvage   2
GO:0006183 GTP biosynthetic process   2
GO:0006217 deoxycytidine catabolic process   1
GO:0006216 cytidine catabolic process   1
GO:0046037 GMP metabolic process   1
GO:0046132 pyrimidine ribonucleoside biosynthetic process 2

00982|Drug metabolism - cytochrome P450|8|10
GO:0019483 beta-alanine biosynthetic process   2
GO:0033859 furaldehyde metabolic process 1
GO:0043458 ethanol biosynthetic process during fermentation   2
GO:0006950 response to stress   1
GO:0006598 polyamine catabolic process   2
GO:0006116 NADH oxidation   4
GO:0006067 ethanol metabolic process   1
GO:0006113 fermentation   1
GO:0046294 formaldehyde catabolic process   1
GO:0000947 amino acid catabolic process to alcohol via Ehrlich pathway 6

00980|Metabolism of xenobiotics by cytochrome P450|8|10
GO:0019483 beta-alanine biosynthetic process   2
GO:0033859 furaldehyde metabolic process 1
GO:0043458 ethanol biosynthetic process during fermentation   2
GO:0006950 response to stress   1
GO:0006598 polyamine catabolic process   2
GO:0006116 NADH oxidation   4
GO:0006067 ethanol metabolic process   1
GO:0006113 fermentation   1
GO:0046294 formaldehyde catabolic process   1
GO:0000947 amino acid catabolic process to alcohol via Ehrlich pathway 6

00632|Benzoate degradation via CoA ligation|6|4
GO:0006474 N-terminal protein amino acid acetylation 1
GO:0006635 fatty acid beta-oxidation   1
GO:0045449 regulation of transcription   1
GO:0016579 protein deubiquitination   1

04130|SNARE interactions in vesicular transport|23|19
GO:0034727 piecemeal microautophagy of nucleus 2
GO:0006888 ER to Golgi vesicle-mediated transport   5
GO:0030437 ascospore formation   1
GO:0042493 response to drug 1

GO:0006944 membrane fusion   1
GO:0006897 endocytosis 2
GO:0006896 Golgi to vacuole transport   5
GO:0031321 ascospore-type prospore formation   3
GO:0006893 Golgi to plasma membrane transport 5
GO:0006891 intra-Golgi vesicle-mediated transport   6
GO:0006890 retrograde vesicle-mediated transport, Golgi to ER   6
GO:0032527 protein exit from endoplasmic reticulum   1
GO:0045324 late endosome to vacuole transport 1
GO:0006675 mannose inositol phosphoceramide metabolic process   1
GO:0006674 inositol phosphorylceramide metabolic process   1
GO:0042144 vacuole fusion, non-autophagic   1
GO:0032581 ER-dependent peroxisome biogenesis 1
GO:0006810 transport   1
GO:0006906 vesicle fusion   15

00770|Pantothenate and CoA biosynthesis|10|5
GO:0009083 branched chain family amino acid catabolic process   2
GO:0000002 mitochondrial genome maintenance   1
GO:0009082 branched chain family amino acid biosynthetic process 6
GO:0015937 coenzyme A biosynthetic process   1
GO:0015940 pantothenate biosynthetic process   2

00220|Urea cycle and metabolism of amino groups|19|24
GO:0019547 arginine catabolic process to ornithine   1
GO:0006090 pyruvate metabolic process   1
GO:0006741 NADP biosynthetic process   1
GO:0006740 NADPH regeneration   1
GO:0006526 arginine biosynthetic process 6
GO:0008295 spermidine biosynthetic process   2
GO:0007047 cell wall organization 1
GO:0043419 urea catabolic process 1
GO:0009651 response to salt stress 1
GO:0019413 acetate biosynthetic process 3
GO:0019509 methionine salvage   1
GO:0006537 glutamate biosynthetic process   1
GO:0006597 spermine biosynthetic process 2
GO:0045449 regulation of transcription   1
GO:0000053 argininosuccinate metabolic process 1
GO:0000052 citrulline metabolic process 1
GO:0034605 cellular response to heat   1
GO:0000256 allantoin catabolic process   1
GO:0006561 proline biosynthetic process 2
GO:0006592 ornithine biosynthetic process   3
GO:0006591 ornithine metabolic process   1
GO:0015940 pantothenate biosynthetic process   4
GO:0009446 putrescine biosynthetic process   1
GO:0006067 ethanol metabolic process   1

00271|Methionine metabolism|19|24
GO:0006312 mitotic recombination   1
GO:0019346 transsulfuration 4
GO:0019344 cysteine biosynthetic process 2
GO:0019343 cysteine biosynthetic process via cystathionine 2
GO:0009086 methionine biosynthetic process   3
GO:0000096 sulfur amino acid metabolic process 1
GO:0042493 response to drug 3
GO:0070814 hydrogen sulfide biosynthetic process   1
GO:0006431 methionyl-tRNA aminoacylation 3
GO:0016259 selenocysteine metabolic process   1
GO:0006556 S-adenosylmethionine biosynthetic process 2

GO:0008295 spermidine biosynthetic process 2
GO:0006555 methionine metabolic process 4
GO:0009092 homoserine metabolic process 1
GO:0006413 translational initiation 1
GO:0006878 cellular copper ion homeostasis 1
GO:0019509 methionine salvage 2
GO:0006537 glutamate biosynthetic process 1
GO:0009636 response to toxin 1
GO:0006535 cysteine biosynthetic process from serine 1
GO:0006597 spermine biosynthetic process 1
GO:0006534 cysteine metabolic process 1
GO:0015940 pantothenate biosynthetic process 2
GO:0006790 sulfur metabolic process 1

00272|Cysteine metabolism|11|18
GO:0006312 mitotic recombination 1
GO:0019346 transsulfuration 3
GO:0019344 cysteine biosynthetic process 3
GO:0019343 cysteine biosynthetic process via cystathionine 1
GO:0009086 methionine biosynthetic process 1
GO:0000096 sulfur amino acid metabolic process 1
GO:0042493 response to drug 2
GO:0006555 methionine metabolic process 1
GO:0006878 cellular copper ion homeostasis 1
GO:0006567 threonine catabolic process 1
GO:0009636 response to toxin 1
GO:0006534 cysteine metabolic process 1
GO:0006532 aspartate biosynthetic process 1
GO:0009071 serine family amino acid catabolic process 1
GO:0006423 cysteinyl-tRNA aminoacylation 1
GO:0001302 replicative cell aging 1
GO:0001300 chronological cell aging 1
GO:0006790 sulfur metabolic process 1

00072|Synthesis and degradation of ketone bodies|2|1
GO:0006696 ergosterol biosynthetic process 1

00430|Taurine and hypotaurine metabolism|2|5
GO:0034605 cellular response to heat 2
GO:0006538 glutamate catabolic process 1
GO:0006979 response to oxidative stress 1
GO:0006751 glutathione catabolic process 1
GO:0042908 xenobiotic transport 1

00071|Fatty acid metabolism|17|17
GO:0006741 NADP biosynthetic process 1
GO:0006740 NADPH regeneration 1
GO:0033859 furaldehyde metabolic process 1
GO:0043458 ethanol biosynthetic process during fermentation 2
GO:0006629 lipid metabolic process 4
GO:0006869 lipid transport 2
GO:0034605 cellular response to heat 3
GO:0006499 N-terminal protein myristoylation 4
GO:0030476 ascospore wall assembly 1
GO:0019413 acetate biosynthetic process 3
GO:0006116 NADH oxidation 4
GO:0006067 ethanol metabolic process 2
GO:0009651 response to salt stress 1
GO:0006113 fermentation 1
GO:0046294 formaldehyde catabolic process 1
GO:0000947 amino acid catabolic process to alcohol via Ehrlich pathway

6
GO:0006090 pyruvate metabolic process 1

03010|Ribosome|122|24
GO:0006407 rRNA export from nucleus 15
GO:0042493 response to drug 2
GO:0030490 maturation of SSU-rRNA 1
GO:0045903 positive regulation of translational fidelity 2
GO:0000465 exonucleolytic trimming to generate mature 5'-end of 5.8S rRNA from tricistronic rRNA transcript (SSU-rRNA, 5.8S rRNA, LSU-rRNA) 1
GO:0016567 protein ubiquitination 1
GO:0042274 ribosomal small subunit biogenesis 2
GO:0042273 ribosomal large subunit biogenesis 2
GO:0000463 maturation of LSU-rRNA from tricistronic rRNA transcript (SSU-rRNA, 5.8S rRNA, LSU-rRNA) 1
GO:0032543 mitochondrial translation 3
GO:0000462 maturation of SSU-rRNA from tricistronic rRNA transcript (SSU-rRNA, 5.8S rRNA, LSU-rRNA) 16
GO:0000461 endonucleolytic cleavage to generate mature 3'-end of SSU-rRNA from (SSU-rRNA, 5.8S rRNA, LSU-rRNA) 4
GO:0048025 negative regulation of nuclear mRNA splicing, via spliceosome 1
GO:0006415 translational termination 1
GO:0000479 endonucleolytic cleavage of tricistronic rRNA transcript (SSU-rRNA, 5.8S rRNA, LSU-rRNA) 1
GO:0017148 negative regulation of translation 1
GO:0006412 translation 113
GO:0000447 endonucleolytic cleavage in ITS1 to separate SSU-rRNA from 5.8S rRNA and LSU-rRNA from tricistronic rRNA transcript (SSU-rRNA, 5.8S rRNA, LSU-rRNA) 5
GO:0042254 ribosome biogenesis 4
GO:0000028 ribosomal small subunit assembly 10
GO:0006364 rRNA processing 1
GO:0000027 ribosomal large subunit assembly 10
GO:0002109 maturation of SSU-rRNA from tricistronic rRNA transcript (SSU-rRNA, LSU-rRNA,5S) 1
GO:0006450 regulation of translational fidelity 3

00640|Propanoate metabolism|11|17
GO:0006606 protein import into nucleus 1
GO:0006104 succinyl-CoA metabolic process 2
GO:0006741 NADP biosynthetic process 1
GO:0006740 NADPH regeneration 1
GO:0019740 nitrogen utilization 1
GO:0009450 gamma-aminobutyric acid catabolic process 1
GO:0034605 cellular response to heat 2
GO:0016573 histone acetylation 2
GO:0006998 nuclear envelope organization 1
GO:0006099 tricarboxylic acid cycle 2
GO:0019654 acetate fermentation 1
GO:0019413 acetate biosynthetic process 3
GO:0006067 ethanol metabolic process 1
GO:0009651 response to salt stress 1
GO:0019541 propionate metabolic process 1
GO:0006090 pyruvate metabolic process 1
GO:0006085 acetyl-CoA biosynthetic process 2

00500|Starch and sucrose metabolism|41|40
GO:0030476 ascospore wall assembly 2
GO:0006008 glucose 1-phosphate utilization 2

GO:0019388 galactose catabolic process 2
GO:0006006 glucose metabolic process 3
GO:0005980 glycogen catabolic process 3
GO:0006098 pentose-phosphate shunt 1
GO:0006096 glycolysis 4
GO:0006000 fructose metabolic process 2
GO:0006094 gluconeogenesis 1
GO:0007047 cell wall organization 2
GO:0006897 endocytosis 1
GO:0045490 pectin catabolic process 1
GO:0042710 biofilm formation 1
GO:0005978 glycogen biosynthetic process 6
GO:0030437 ascospore formation 2
GO:0005975 carbohydrate metabolic process 1
GO:0006950 response to stress 6
GO:0001403 invasive growth in response to glucose limitation 1
GO:0008361 regulation of cell size 2
GO:0046015 regulation of transcription by glucose 1
GO:0001302 replicative cell aging 1
GO:0005993 trehalose catabolic process 3
GO:0005992 trehalose biosynthetic process 7
GO:0007124 pseudohyphal growth 2
GO:0034605 cellular response to heat 7
GO:0006078 1,6-beta-glucan biosynthetic process 1
GO:0006013 mannose metabolic process 3
GO:0006011 UDP-glucose metabolic process 3
GO:0006075 1,3-beta-glucan biosynthetic process 2
GO:0030003 cellular cation homeostasis 1
GO:0006073 cellular glucan metabolic process 1
GO:0000128 flocculation 1
GO:0032445 fructose import 2
GO:0006874 cellular calcium ion homeostasis 1
GO:0042493 response to drug 1
GO:0000023 maltose metabolic process 1
GO:0006486 protein amino acid glycosylation 1
GO:0005987 sucrose catabolic process 1
GO:0046323 glucose import 3
GO:0030447 filamentous growth 1

00641|3-Chloroacrylic acid degradation|9|13
GO:0006741 NADP biosynthetic process 1
GO:0006740 NADPH regeneration 1
GO:0033859 furaldehyde metabolic process 1
GO:0043458 ethanol biosynthetic process during fermentation 2
GO:0034605 cellular response to heat 1
GO:0019413 acetate biosynthetic process 3
GO:0006116 NADH oxidation 4
GO:0006067 ethanol metabolic process 2
GO:0009651 response to salt stress 1
GO:0006113 fermentation 1
GO:0046294 formaldehyde catabolic process 1
GO:0000947 amino acid catabolic process to alcohol via Ehrlich pathway 6
GO:0006090 pyruvate metabolic process 1

00642|Ethylbenzene degradation|3|1
GO:0006474 N-terminal protein amino acid acetylation 1

00970|Aminoacyl-tRNA biosynthesis|39|43
GO:0006420 arginyl-tRNA aminoacylation 1
GO:0000372 Group I intron splicing 1

GO:0006353 transcription termination   1
GO:0051276 chromosome organization 1
GO:0070149 mitochondrial glutamyl-tRNA aminoacylation   1
GO:0006419 alanyl-tRNA aminoacylation   1
GO:0070146 mitochondrial aspartyl-tRNA aminoacylation   1
GO:0070145 mitochondrial asparaginyl-tRNA aminoacylation   1
GO:0070144 mitochondrial arginyl-tRNA aminoacylation 1
GO:0070143 mitochondrial alanyl-tRNA aminoacylation 1
GO:0006413 translational initiation   1
GO:0006412 translation 3
GO:0009060 aerobic respiration   2
GO:0070681 glutaminyl-tRNAGln biosynthesis via transamidation   2
GO:0001403 invasive growth in response to glucose limitation   1
GO:0006438 valyl-tRNA aminoacylation   1
GO:0006437 tyrosyl-tRNA aminoacylation   2
GO:0006436 tryptophanyl-tRNA aminoacylation   1
GO:0006435 threonyl-tRNA aminoacylation 1
GO:0006434 seryl-tRNA aminoacylation   1
GO:0006432 phenylalanyl-tRNA aminoacylation   3
GO:0006431 methionyl-tRNA aminoacylation 3
GO:0006430 lysyl-tRNA aminoacylation   1
GO:0007124 pseudohyphal growth   1
GO:0032543 mitochondrial translation   7
GO:0007029 endoplasmic reticulum organization 1
GO:0016031 cytoplasmic tRNA import into mitochondrion   1
GO:0070159 mitochondrial threonyl-tRNA aminoacylation   1
GO:0006429 leucyl-tRNA aminoacylation   2
GO:0070158 mitochondrial seryl-tRNA aminoacylation   1
GO:0006428 isoleucyl-tRNA aminoacylation 1
GO:0006427 histidyl-tRNA aminoacylation 1
GO:0070156 mitochondrial phenylalanyl-tRNA aminoacylation 1
GO:0006426 glycyl-tRNA aminoacylation   1
GO:0006425 glutaminyl-tRNA aminoacylation   1
GO:0070154 mitochondrial lysyl-tRNA aminoacylation   1
GO:0006424 glutamyl-tRNA aminoacylation 1
GO:0070152 mitochondrial isoleucyl-tRNA aminoacylation   1
GO:0006423 cysteinyl-tRNA aminoacylation 1
GO:0006422 aspartyl-tRNA aminoacylation 2
GO:0070183 mitochondrial tryptophanyl-tRNA aminoacylation 1
GO:0006421 asparaginyl-tRNA aminoacylation   2
GO:0070150 mitochondrial glycyl-tRNA aminoacylation 1

00785|Lipoic acid metabolism|3|2
GO:0009105 lipoic acid biosynthetic process   1
GO:0009249 protein lipoylation   3

00590|Arachidonic acid metabolism|5|6
GO:0006629 lipid metabolic process 1
GO:0034605 cellular response to heat   1
GO:0030163 protein catabolic process   1
GO:0006979 response to oxidative stress 3
GO:0006751 glutathione catabolic process 1
GO:0042908 xenobiotic transport   1

04140|Regulation of autophagy|17|29
GO:0034727 piecemeal microautophagy of nucleus 15
GO:0048017 inositol lipid-mediated signaling   1
GO:0045053 protein retention in Golgi apparatus   1
GO:0006888 ER to Golgi vesicle-mediated transport   1
GO:0032147 activation of protein kinase activity   2
GO:0006468 protein amino acid phosphorylation 3

GO:0030433 ER-associated protein catabolic process   2
GO:0006464 protein modification process 1
GO:0006914 autophagy   4
GO:0006944 membrane fusion   1
GO:0006661 phosphatidylinositol biosynthetic process 1
GO:0000045 autophagic vacuole formation 5
GO:0030242 peroxisome degradation 2
GO:0000011 vacuole inheritance   1
GO:0046854 phosphoinositide phosphorylation   1
GO:0051605 protein maturation by peptide bond cleavage   1
GO:0045324 late endosome to vacuole transport 2
GO:0016237 microautophagy   1
GO:0006508 proteolysis 1
GO:0006612 protein targeting to membrane 2
GO:0016236 macroautophagy   9
GO:0042147 retrograde transport, endosome to Golgi   1
GO:0034497 protein localization to pre-autophagosomal structure 1
GO:0032446 protein modification by small protein conjugation   1
GO:0042144 vacuole fusion, non-autophagic   1
GO:0006501 C-terminal protein lipidation 4
GO:0034605 cellular response to heat   2
GO:0032258 CVT pathway 9
GO:0006623 protein targeting to vacuole 2

00592|alpha-Linolenic acid metabolism|2|0

00780|Biotin metabolism|7|4
GO:0045047 protein targeting to ER 2
GO:0009102 biotin biosynthetic process   3
GO:0009305 protein amino acid biotinylation   1
GO:0006465 signal peptide processing   2

00280|Valine, leucine and isoleucine degradation|10|23
GO:0006312 mitotic recombination   1
GO:0006546 glycine catabolic process   1
GO:0042743 hydrogen peroxide metabolic process 1
GO:0006090 pyruvate metabolic process   2
GO:0006574 valine catabolic process   1
GO:0009083 branched chain family amino acid catabolic process   2
GO:0009082 branched chain family amino acid biosynthetic process 2
GO:0045144 meiotic sister chromatid segregation   1
GO:0006741 NADP biosynthetic process   1
GO:0006740 NADPH regeneration   1
GO:0051315 attachment of spindle microtubules to kinetochore during mitosis   1
GO:0006696 ergosterol biosynthetic process   1
GO:0045931 positive regulation of mitotic cell cycle 1
GO:0034453 microtubule anchoring   1
GO:0006552 leucine catabolic process   1
GO:0009651 response to salt stress 1
GO:0006550 isoleucine catabolic process 1
GO:0006103 2-oxoglutarate metabolic process   1
GO:0019413 acetate biosynthetic process 3
GO:0006564 L-serine biosynthetic process 1
GO:0034605 cellular response to heat   1
GO:0007020 microtubule nucleation 1
GO:0006067 ethanol metabolic process   1

00900|Terpenoid biosynthesis|5|3
GO:0008299 isoprenoid biosynthetic process   1
GO:0016114 terpenoid biosynthetic process   1

GO:0045337 farnesyl diphosphate biosynthetic process 1

00440|Aminophosphonate metabolism|7|7
GO:0000466 maturation of 5.8S rRNA from tricistronic rRNA transcript (SSU-rRNA, 5.8S rRNA, LSU-rRNA) 1
GO:0000154 rRNA modification 1
GO:0000463 maturation of LSU-rRNA from tricistronic rRNA transcript (SSU-rRNA, 5.8S rRNA, LSU-rRNA) 1
GO:0006646 phosphatidylethanolamine biosynthetic process 1
GO:0006657 CDP-choline pathway 1
GO:0031167 rRNA methylation 1
GO:0006656 phosphatidylcholine biosynthetic process 1

00903|Limonene and pinene degradation|7|11
GO:0006635 fatty acid beta-oxidation 1
GO:0006741 NADP biosynthetic process 1
GO:0006740 NADPH regeneration 1
GO:0016579 protein deubiquitination 1
GO:0006474 N-terminal protein amino acid acetylation 1
GO:0034605 cellular response to heat 1
GO:0019413 acetate biosynthetic process 3
GO:0006067 ethanol metabolic process 1
GO:0009651 response to salt stress 1
GO:0006090 pyruvate metabolic process 1
GO:0045449 regulation of transcription 1

00310|Lysine degradation|16|30
GO:0051568 histone H3-K4 methylation 1
GO:0051598 meiotic recombination checkpoint 1
GO:0030437 ascospore formation 2
GO:0006090 pyruvate metabolic process 1
GO:0030466 chromatin silencing at silent mating-type cassette 1
GO:0006465 signal peptide processing 2
GO:0006741 NADP biosynthetic process 1
GO:0006740 NADPH regeneration 1
GO:0006355 regulation of transcription, DNA-dependent 1
GO:0006354 RNA elongation 1
GO:0000183 chromatin silencing at rDNA 1
GO:0000077 DNA damage checkpoint 1
GO:0009651 response to salt stress 1
GO:0018027 peptidyl-lysine dimethylation 1
GO:0006103 2-oxoglutarate metabolic process 2
GO:0043618 regulation of transcription from RNA polymerase II promoter in response to stress 1
GO:0045047 protein targeting to ER 2
GO:0019413 acetate biosynthetic process 3
GO:0000725 recombinational repair 1
GO:0000723 telomere maintenance 1
GO:0006301 postreplication repair 1
GO:0006289 nucleotide-excision repair 1
GO:0019878 lysine biosynthetic process via aminoadipic acid 4
GO:0016571 histone methylation 1
GO:0034605 cellular response to heat 1
GO:0018065 protein-cofactor linkage 1
GO:0006348 chromatin silencing at telomere 2
GO:0006099 tricarboxylic acid cycle 2
GO:0006067 ethanol metabolic process 1
GO:0034729 histone H3-K79 methylation 1
