# Supplementary material for: Quantification of protein group coherence and pathway assignment using functional association
Source: BMC Bioinformatics. 2011 Sep 19;12:373. doi: 10.1186/1471-2105-12-373 (PMC3189934; doi:10.1186/1471-2105-12-373)
Supplement: Additional file 2 — Supplementary Figures S1-S4. Coherence score distribution for different datasets. [file 1471-2105-12-373-S2.PDF]

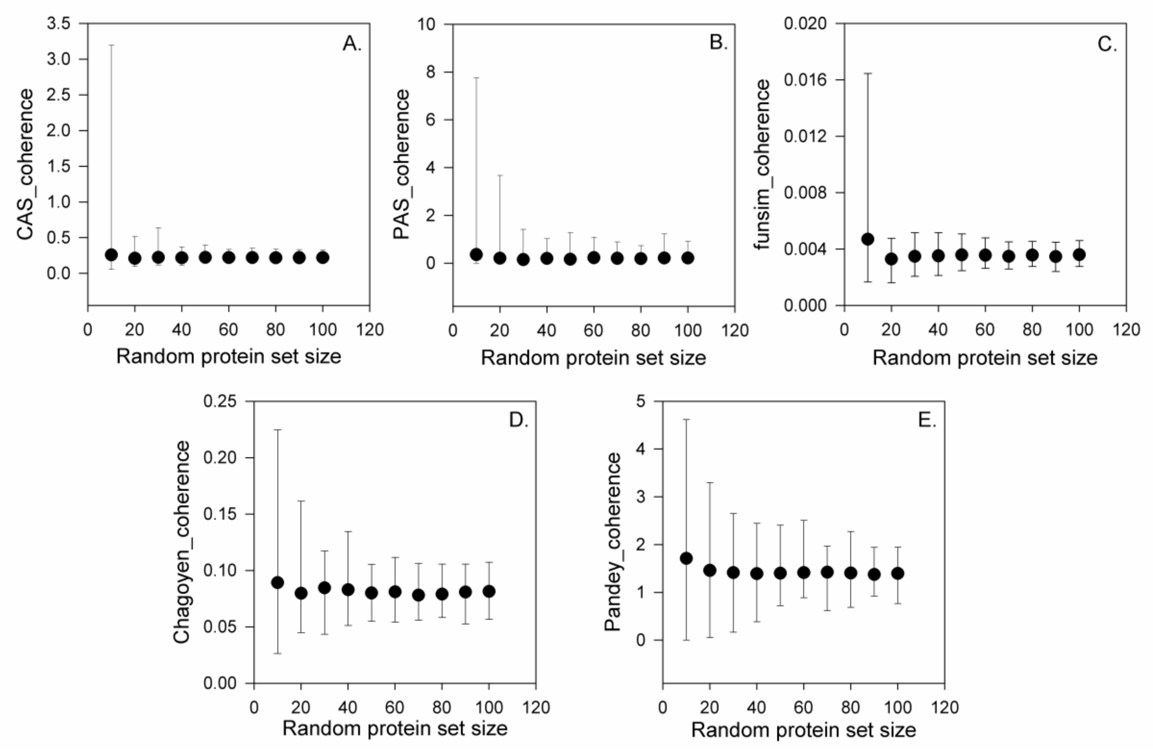

Figure S1: Coherence score distributions for Random sets

A. CAS_coherence score distribution for Random sets, B. PAS_coherence score distribution for Random sets, C. funsim_coherence score distribution for Random sets, D. Chagoyen_coherence score distribution for Random sets, E. Pandey_coherence score distribution for Random sets

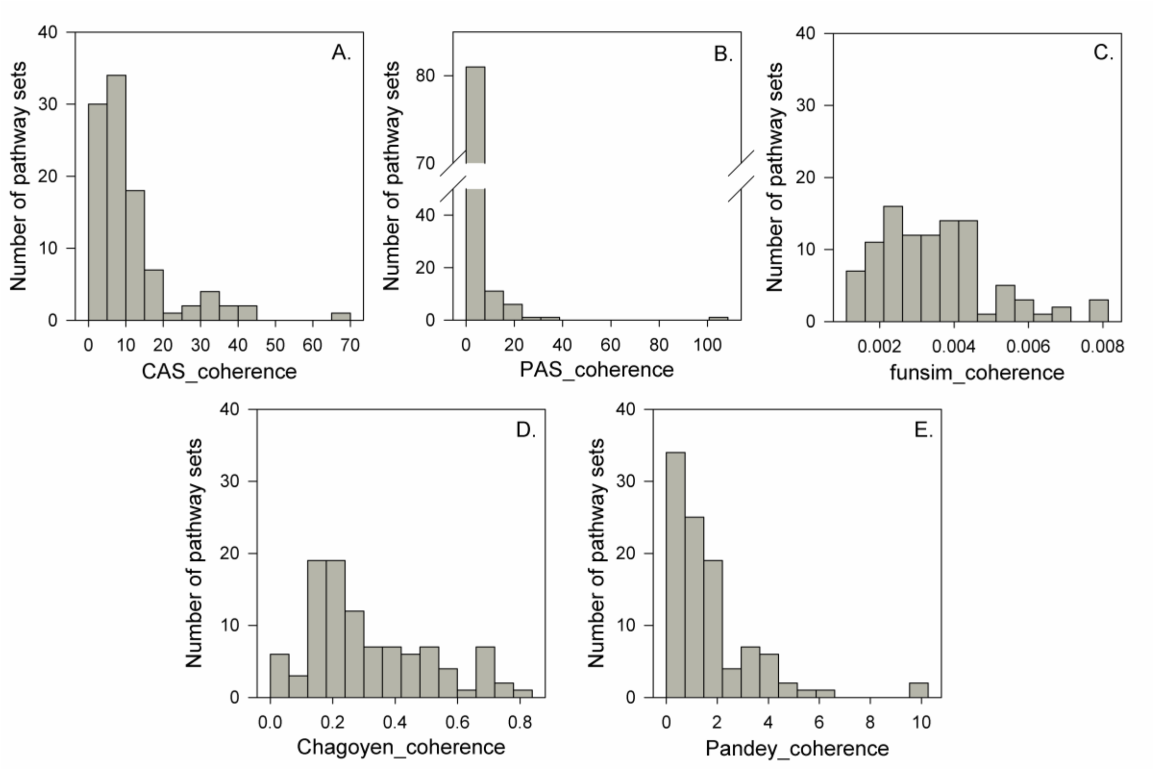

Figure S2: Coherence score distributions for Pathway sets

A. CAS_coherence score distribution for Pathway sets, B. PAS_coherence score distribution for Pathway sets, C. funsim_coherence score distribution for Pathway sets, D. Chagoyen_coherence score distribution for Pathway sets, E. Pandey_coherence score distribution for Pathway sets

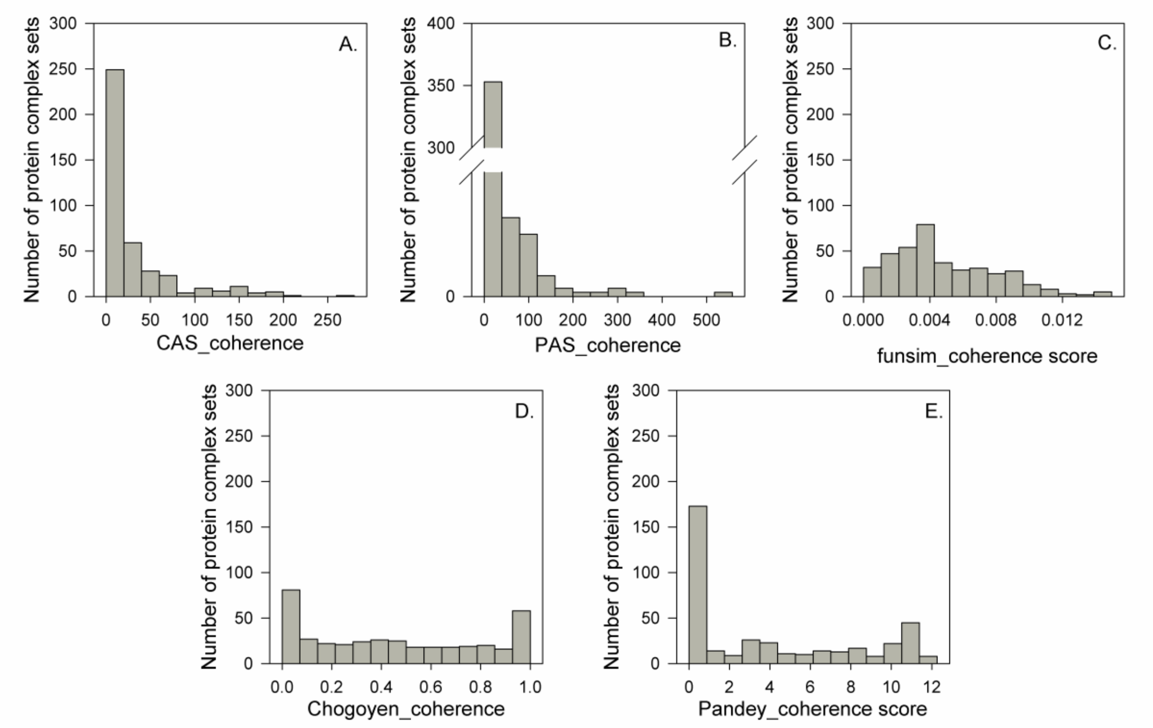

Figure S3: Coherence score distributions for Protein complex sets

A. CAS_coherence score distribution for Protein complex sets, B. PAS_coherence score distribution for Protein complex sets, C. funsim_coherence score distribution for Protein complex sets, D. Chagoyen_coherence score distribution for Protein complex sets, E. Pandey_coherence score distribution for Protein complex sets

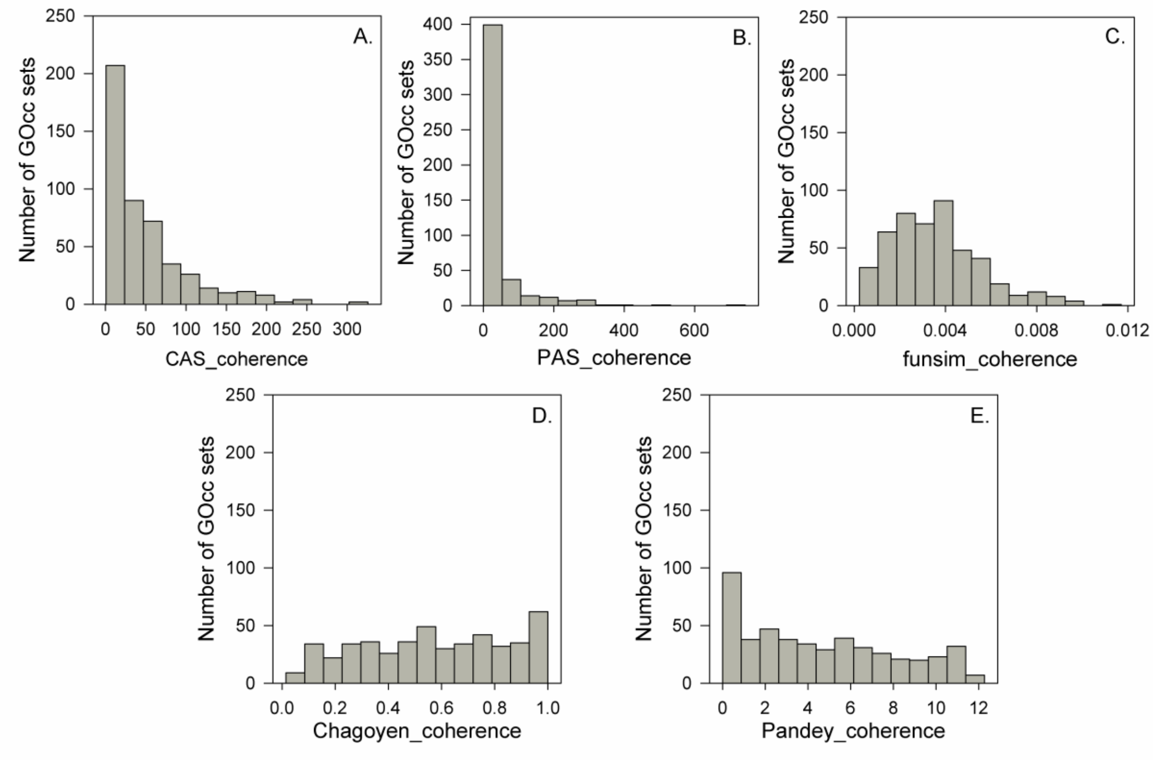

Figure S4: Coherence score distributions for GOcc sets

A. CAS_coherence score distribution for GOcc sets, B. PAS_coherence score distribution for GOcc sets, C. funsim_coherence score distribution for GOcc sets, D. Chagoyen_coherence score distribution for GOcc sets, E. Pandey_coherence score distribution for GOcc sets
